# Electrochemical Cycloaddition Reactions of Alkene Radical Cations: A Route toward Cyclopropanes and Cyclobutanes

**DOI:** 10.1021/acs.orglett.3c00121

**Published:** 2023-02-14

**Authors:** Katarzyna Rybicka-Jasińska, Zuzanna Szeptuch, Hubert Kubiszewski, Agnieszka Kowaluk

**Affiliations:** † Institute of Organic Chemistry, Polish Academy of Sciences, Kasprzaka 44/52, 01-224 Warsaw, Poland; ‡ Faculty of Chemistry, Warsaw University of Technology, Noakowskiego 3, 00-664 Warsaw, Poland

## Abstract

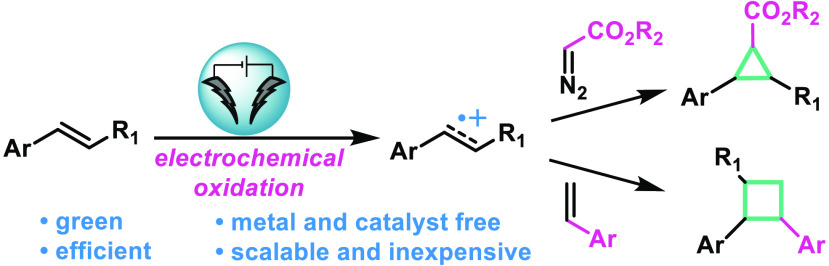

Herein, we describe a mild and efficient electrochemical method for cycloaddition reactions of alkene radical cations. Anodic oxidation of olefins produces electrophilic alkene radical cations, which further react with either diazo compounds in a [2 + 1] cycloaddition toward cyclopropane synthesis, or styrene derivatives in a [2 + 2] cycloaddition producing cyclobutanes. Both processes are green, metal- and catalyst-free, and scalable and tolerate a broad range of electron-rich olefins.

New methods to promote single-electron transfer reactions play an increasingly important role in driving modern organic chemistry. Significant advances have been made in the field of photoredox catalysis^1−4^ and synthetic organic electrochemistry^5−9^ to expand the utility of radicals in organic synthesis.^10,11^

While the initial developments in synthetic organic electrochemistry date as far back as the early 19th century, only recently has it undergone an explosive revival, and it is now an important part of synthetic organic chemistry.^5−11^ Undeniably, it provides a green alternative to known and new reactive intermediates by the promotion of organic reactions under mild conditions without the necessity for excessive amounts of hazardous and wasteful oxidants and reductants, metals, or catalysts, making chemical synthesis highly atom economical.^12^

Olefin-derived radical cations are versatile reactive intermediates and have been widely used in the construction of complex and cyclic systems.^13^ In the case of three-membered ring construction,^14−20^ an electron-rich alkene can be oxidized to an electrophilic alkene radical cation and subsequently can further react with nucleophilic diazo compounds. Such cationic radical cycloaddition reactions have been already accomplished through stoichiometric oxidation by Bauld,^21−23^ photoredox catalysis by Ferreira,^24^ and Fe-catalysis by Kang (Scheme 1a).^25^ Although the presented reports prove undeniably that cyclopropane ring formation can be performed in an efficient manner, all of the above methods rely on the use of either metal catalysts or stoichiometric oxidants; an additional challenge is potential limits: the potential window for oxidation for both catalysts^24,25^ used by Ferreira is about +1.11 to +1.88 V vs SCE, which means that reaction is not applicable for substrates that are either very easy or difficult to oxidize.

**Scheme 1:**
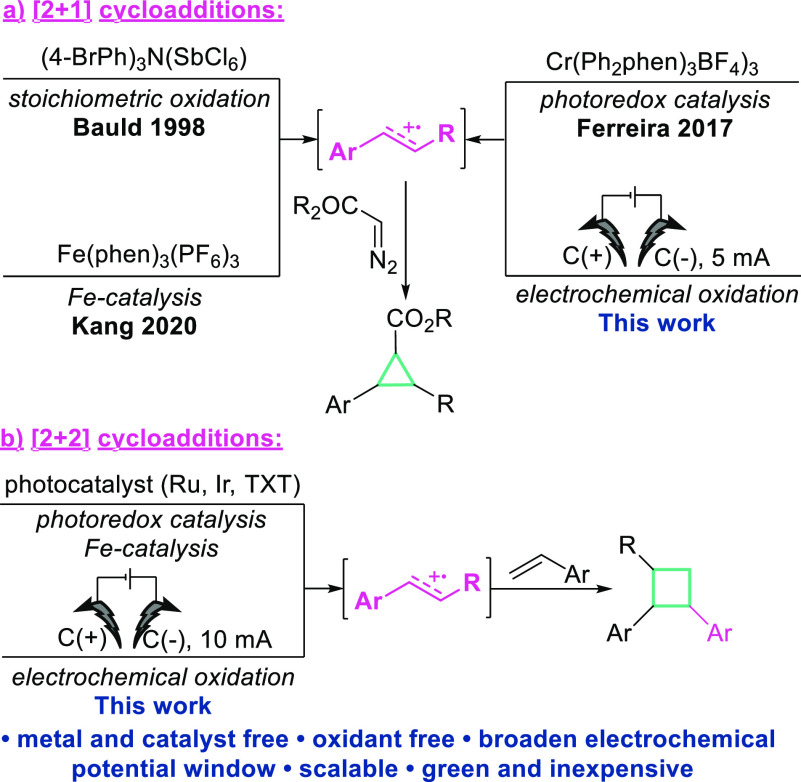
Strategies toward Radical (a) [2 + 1] and (b) [2 + 2] Cycloadditions via Olefin Electro-oxidation to Radical Cations

In addition to being captured by diverse nucleophiles, olefin radical cations can also react with double bonds to build cyclic structural units, mainly four-membered and six-membered ring derivatives.^13,26−29^

Photochemical [2 + 2] cycloaddition reactions are one method of direct access to substituted cyclobutanes.^30−36^ Among the known photocatalytic [2 + 2] cycloadditions, oxidative cross-intermolecular [2 + 2] cycloadditions are attractive reactions that can facilitate the regio- and stereoselective synthesis of cyclobutanes (Scheme 1b).^30−36^ Despite the various advantages of photoredox catalysis in cross-intermolecular [2 + 2] cycloadditions, the utilization of electrochemistry could further improve such an approach by employing metal- and catalyst-free conditions, enhancing scalability, as well as cost-effectiveness. As early as 2001, Chiba reported that electron-rich enol ethers could be used as radical cation precursors under electrochemical conditions to trigger intermolecular [2 + 2] cyclization for the construction of four-membered rings.^37,38^ However, for this electrochemical transformation, it is necessary to use an enol ether that bears a methoxyphenyl group. *Generally, the electrochemical approach for the application of alkene radical cations for the formation of three-membered and four-membered rings is relatively underexplored and to the best of our knowledge it could be beneficial in terms of atom economy (by applying metal-, catalyst-, and external-oxidant-free conditions), green chemistry, scalability, cost-effectiveness, and broadening the scope of the methods by shifting the electrochemical potential window for various substrates*.

Given the aforementioned consideration, we have developed a strategy for the electrochemical cycloaddition of olefin radical cations with diazo compounds and styrene derivatives toward the synthesis three and four-membered rings (Scheme 1). Our novel procedure opens doors for the synthesis of cyclopropanes and cyclobutanes in an efficient, metal- and catalyst-free, environmentally friendly, and inexpensive way.

In order to establish the [2 + 1] electrochemical cycloaddition of alkene radical cations, we initiated our studies by exploring the reactivity of ethyl diazoacetate (EDA, **2**) with *trans*-stilbene (**1**, *E*_1/2_ = 1.48 V vs Ag/AgCl) in the presence of the commonly used electrolyte (NBu_4_PF_4_), under a constant current (10 mA). The reaction produced the desired cyclopropane **3** in 41% yield as the main product (Table 1, entry 3). Subsequently, several reaction parameters (solvent, substrate ratio, concentration, type of electrolyte, time, and electrical power; for details see SI) were optimized. *It is worth mentioning that the conditions of the reaction were optimized exclusively with the use of graphite electrodes for environmentally friendly, inexpensive, and metal-free conditions.* Overall, anodic oxidation with constant electrical power (*I* = 5 mA) of olefin **1a** and diazo compound **2a** (molar ratio 1:1.2) with graphite electrodes and NBu_4_PF_6_ (*c* = 0.125 M) at room temperature for 12 h gives the cyclopropane derivative as product **3** in 80% yield. Background experiments confirmed that the desired transformation cannot take place without electricity (entry 2); however the reaction can proceed without electrolyte, although the yield drops to 40% (entry 9).^39^

**Table 1:**
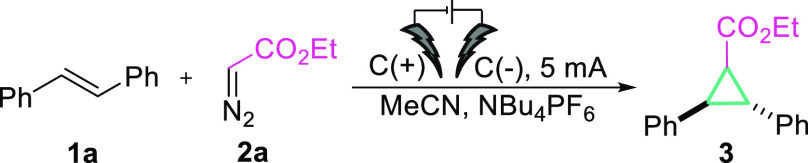
Optimization of Reaction Conditions^a^

entry	variation from standard conditions	yield [%]^b^
**1**	**none**	**80**
2	without electricity	0
3	10 mA instead of 5 mA, 4 h	48
4	DCE instead of MeCN	77
5	MeOH, DMF, THF instead of MeCN	0–40
6	LiCF_3_SO_2_ instead of NBu_4_PF_6_	75
7	LiBF_4_ instead of NBu_4_PF_6_	47
8	NBu_4_ClO_4_ instead of NBu_4_PF_6_	38
9	no electrolyte	40

a Reaction conditions: *trans*-stilbene **1a** (0.6 mmol), ethyl diazoacetate **2a** (1.2 equiv), dry MeCN (*c* = 0.15 M), NBu_4_PF_6_ (0.125 mmol), *I* = 5 mA, graphite electrodes (C(+)|(C(−)), rt, 12 h, under Ar atmosphere.

b Isolated yield.

With the optimized conditions in hand, we examined a set of electron-rich olefins and various diazo carbonyl compounds in a scope and limitations study (Scheme 2). The electrochemical cyclopropanation was applicable to symmetrical and unsymmetrical stilbenes with various functional groups as substituents (OMe, Cl, Br, CO_2_Et, NO_2_) giving the desired products **3**–**12** in good yields. As the dinitro substituted stilbene derivative is not soluble in the examined solvents (MeCN, DCE, DCM), it remains unproductive in the studied transformation (**7**). Aryl alkyl alkenes were also participatory in this cycloaddition yielding products in good yields (40–72%). For unsymmetrical olefins, the yields were generally good, but little diastereoselectivity was achieved (approximately 1:1 dr). Tetrasubstituted olefin unfortunately proved unreactive (**16**). It is worth mentioning that in our method stilbenes even with an extremely withdrawing group such as NO_2_ remain reactive (**8**, 58%) because the electrochemical method can overcome the electrochemical window required for both the photoredox approach^24^ and iron(III)-phenanthroline complex oxidation.^25^ Moreover, since electrochemical methods are known for their perfect scalability, as expected, the reaction proved to be easily scalable (see 1.2 mmol scale for product **3**, 74%, and for product **14**, 70%).

**Scheme 2:**
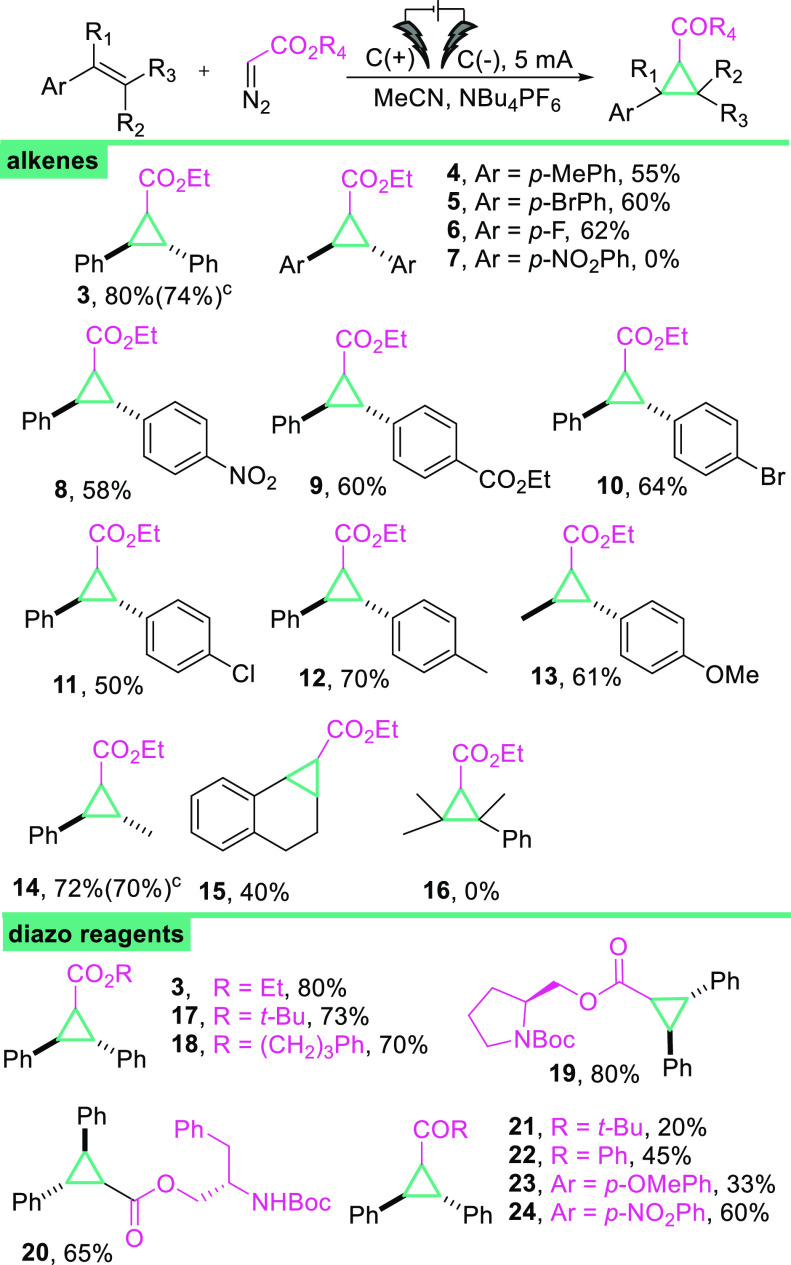
Scope and Limitations Study for Electrochemical Cyclopropanation, Reaction conditions: alkene (0.6 mmol), diazo compound (1.2 equiv), dry MeCN (*c* = 0.15 M), NBu_4_PF_6_ (*c* = 0.125 M), *I* = 5 mA, graphite electrodes (C(+)|(C(−)), rt, specific time see SI, under Ar atmosphere. Yields reported are isolated yields. 1.2 mmol scale.

Next, we investigated the scope of diazo reagents with respect to the carbonyl functional group and α-substituents. Different diazo esters produced cyclopropanes (**3**, **17**–**20**) with good to very good yields. Furthermore, it is noteworthy that the reaction with diazoacetate **2d** consisting of an l-proline moiety afforded the desired compound (**19**) in 80% yield. Diazo ketones successfully participated in the electrochemical reaction, providing cyclopropanes (**21**–**24**), although with lower yields (20–60%) perhaps due to the fact that diazo ketones are known to be less nucleophilic than diazo esters.^40^ Interestingly, olefins with an *E* or *Z* configuration gave only *trans*-cyclopropanes in all cases, suggesting that the reaction proceeds via a radical-cation intermediate (see SI).^36^

After successful application of electron-rich olefins and diazo compounds in an electrochemical formal [2 + 1] cycloaddition, we turned our attention toward the synthesis of four-membered rings. Inspired by the Chiba’s work^37,38^ and our results, we have developed an electrochemical strategy for the construction of cyclobutanes. Since, the first step in such processes is the same as in the case of electrochemical cyclopropanation (oxidation of olefin to radical cation), we were curious whether this transformation could be also achieved using our electrochemical system. To our delight, the reaction between *trans*-anathole (**1k**, *E*_1/2_ = 1.23 V vs Ag/AgCl) and styrene (**25**, *E*_1/2_ = 1.97 V vs Ag/AgCl) under a constant current (5 mA) yields the cyclobutane product (**26**) in 39% yield (Table 2). Subsequently, several reaction parameters (substrate ratio, concentration, electrolyte, time, and electrical power; for details see SI) were optimized. Overall, anodic oxidation of **1k** with **25a** (molar ratio 1:7.5) and NBu_4_PF_6_ (*c* = 0.125M) under galvanostatic conditions (*I* = 10 mA) at room temperature for 5 h gives the cyclobutane derivative as product **26** in 70% yield. Background experiments confirmed that the application of the electrical power is crucial for the reaction to proceed (see SI).

**Table 2:**
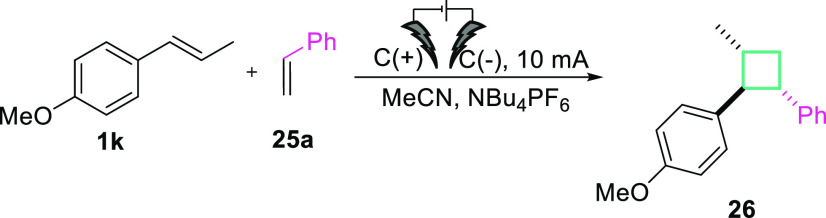
Optimization Studies for Electrochemical [2 + 2] Cycloaddition of Styrenes^a^

entry	variation from standard conditions	yield [%]^b^
**1**	**none**	**70**
2	without electricity	0
3	5 mA instead of 10 mA, 10 h	39
6	LiCF_3_SO_2_ instead of NBu_4_PF_6_	39
7	NBu_4_ClO_4_ instead of NBu_4_PF_6_	18
8	no electrolyte	20

a Reaction conditions: *trans*-anathole **1k** (0.6 mmol), styrene **25** (7.5 equiv), MeCN (*c* = 0.15 M), NBu_4_PF_6_ (*c* = 0.125 M), *I* = 10 mA, graphite electrodes (C(+)|(C(−)), rt, 5 h, under Ar atmosphere.

b Isolated yields.

With the optimized conditions in hand, we examined a small scope and limitations study. Formation of cyclobutanes proceeds very well for styrenes with various functional groups upon the phenyl substituent (Me, Cl, Br, F) giving the desired products **26**–**30** in good to very good yields (Figure 1). However, the reaction proved ineffective when a different alkene radical cation precursor was used (**31**).

**Figure 1:**
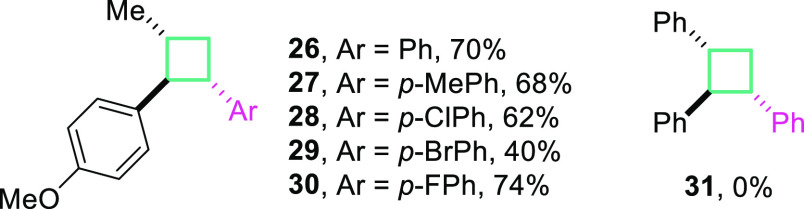
Cycloaddition reactions of alkene radical cations with styrene derivatives. Reaction conditions: *trans*-anathole **1k** (0.6 mmol), styrene **25** (7.5 equiv), MeCN (*c* = 0.15 M), NBu_4_PF_6_ (*c* = 0.125 M), *I* = 10 mA, graphite electrodes (C(+)|(C(−)), rt, 5 h, under Ar atmosphere. Yields reported are isolated yields.

Based on the aforementioned results and the literature data,^21−25,29−38^ we propose a plausible radical reaction pathway for the electrochemical [2 + 1] and [2 + 2] cycloadditions of alkene cation radicals (Scheme 3). The first step is the same for both transformations, which is anodic oxidation of the olefin **A** that generates an olefin electrophilic radical cation **B**. In the case of [2 + 1] cycloaddition, radical cation **B** undergoes a reaction with nucleophilic diazo compound **C** to generate radical cation **D**, which after spontaneous nitrogen extrusion forms radical cation **E**. Subsequent reduction at the cathode generates cyclopropane **F**. In the case of [2 + 2] cycloaddition, radical cation **B** reacts with styrene derivative **G** to form cyclobutanyl radical cation **H**, which after reduction on the cathode forms cyclobutane **I**. Of course, we cannot exclude the possibility of a radical chain reaction and subsequent reduction of radical cation **E** and **H** by olefin **A**.^41^ In each case, the addition of TEMPO stops the reaction, thus confirming the radical nature of both transformations (see SI).

**Scheme 3:**
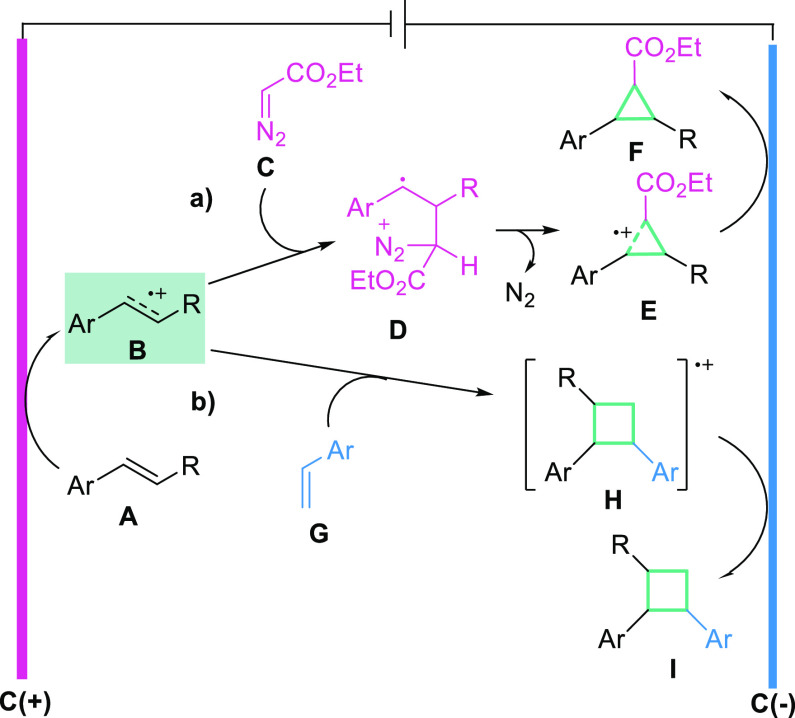
Mechanistic Proposal for the Electrochemical [2 + 1] (path a) and [2 + 2] (path b) Cycloadditions of Alkene Radical Cations

In conclusion, this work describes mild and efficient electrochemical methods for [2 + 1] and [2 + 2] cycloaddition reactions of alkene radical cations. Anodic oxidation of olefins produces electrophilic alkene radical cations, which further react with nucleophilic diazo compounds in a formal [2 + 1] cycloaddition toward cyclopropane synthesis. This methodology is also applicable for [2 + 2] cycloadditions with styrene derivatives, validating this efficient electrochemical system for both cyclopropane and cyclobutane ring synthesis. The advantages of this methods include the mild metal- and catalyst-free conditions, simple setup, and scalability of the procedure, as well as its potential use for industrial applications since the method requires inexpensive graphite electrodes.

## Data Availability

The data underlying this study are available in the published article and its online Supporting Information.

## Supporting Information Available

Optimization details, experimental procedures, and characterization data for all new compounds (PDF)

## References

[ref1] Skubi K. L.; Blum T. R.; Yoon T. P. Dual Catalysis Strategies in Photochemical Synthesis. Chem. Rev. 2016, 116 (17), 10035–10074. 10.1021/acs.chemrev.6b00018. 27109441 PMC5083252

[ref2] Romero N. A.; Nicewicz D. A. Organic Photoredox Catalysis. Chem. Rev. 2016, 116 (17), 10075–10166. 10.1021/acs.chemrev.6b00057. 27285582

[ref3] Prier C. K.; Rankic D. A.; MacMillan D. W. C. Visible Light Photoredox Catalysis with Transition Metal Complexes: Applications in Organic Synthesis. Chem. Rev. 2013, 113 (7), 5322–5363. 10.1021/cr300503r. 23509883 PMC4028850

[ref4] Donabauer K.; König B. Strategies for the Photocatalytic Generation of Carbanion Equivalents for Reductant-Free C–C Bond Formations. Acc. Chem. Res. 2021, 54 (1), 242–252. 10.1021/acs.accounts.0c00620. 33325678 PMC7871440

[ref5] Yan M.; Kawamata Y.; Baran P. S. Synthetic Organic Electrochemical Methods Since 2000: On the Verge of a Renaissance. Chem. Rev. 2017, 117 (21), 13230–13319. 10.1021/acs.chemrev.7b00397. 28991454 PMC5786875

[ref6] Zhu C.; Ang N. W.; Meyer T. A.; Qiu Y.; Ackermann L. Organic Electrochemistry: Molecular Syntheses with Potential. ACS Cent. Sci. 2021, 7 (3), 415–431. 10.1021/acscentsci.0c01532. 33791425 PMC8006177

[ref7] Pollok D.; Waldvogel S. R. Electro-organic synthesis – a 21st century technique. Chem. Sci. 2020, 11, 12386–12400. 10.1039/D0SC01848A. 34123227 PMC8162804

[ref8] Meyer T. H.; Choi I.; Tian C.; Ackermann L. Ackermann Powering the Future: How Can Electrochemistry Make a Difference in Organic Synthesis?. Chem 2020, 6, 2484–2496. 10.1016/j.chempr.2020.08.025.

[ref9] Yan M.; Kawamata Y.; Baran P. S. Synthetic Organic Electrochemistry: Calling All Engineers. Angew. Chem., Int. Ed. 2018, 57, 4149–4155. 10.1002/anie.201707584. PMC5823775 28834012

[ref10] Tay N.; Lehnherr D.; Rovis T. Photons or Electrons? A Critical Comparison of Electrochemistry and Photoredox Catalysis for Organic Synthesis. Chem. Rev. 2022, 122 (2), 2487–2649. 10.1021/acs.chemrev.1c00384. 34751568 PMC10021920

[ref11] Kingston C.; Palkowitz M. D.; Takahira Y.; Vantourout J. C.; Peters B. K.; Kawamata Y.; Baran P. S. A Survival Guide for the “Electro-curious. Acc. Chem. Res. 2020, 53 (1), 72–83. 10.1021/acs.accounts.9b00539. 31823612 PMC6996934

[ref12] Sbei N.; Hardwick T.; Ahmed N. Green-Chemistry: Electrochemical Organic Transformations via Paired Electrolysis. ACS Sustainable Chem. Eng. 2021, 9, 6148–6169. 10.1021/acssuschemeng.1c00665.

[ref13] Luo M.-J.; Xiao Q.; Li J.-H. Electro-/photocatalytic alkene-derived radical cation chemistry: recent advances in synthetic applications. Chem. Soc. Rev. 2022, 51, 7206–7237. 10.1039/D2CS00013J. 35880555

[ref14] Wu W.; Lin Z.; Jiang H. Recent advances in the synthesis of cyclopropanes. Org. Biomol. Chem. 2018, 16, 7315–7329. 10.1039/C8OB01187G. 30229776

[ref15] Doyle P. M.; McKervey A.; Ye T. Modern Catalytic Methods for Organic Synthesis with Diazo Compounds: From Cyclopropanes to Ylides; Wiley, 1998; ISBN: 978-0-471-13556-2.

[ref16] Zhang Y.; Qian R.; Zheng X.; Zeng Y.; Sun J.; Chen Y.; Ding A.; Guo H. Visible light induced cyclopropanation of dibromomalonates with alkenes via double-SET by photoredox catalysis. Chem. Cummun. 2015, 51, 54–57. 10.1039/C4CC08203F. 25406804

[ref17] Nguyen J. D.; Tucker J. W.; Konieczynska M. D.; Stephenson C. R. J. Intermolecular Atom Transfer Radical Addition to Olefins Mediated by Oxidative Quenching of Photoredox Catalysts. J. Am. Chem. Soc. 2011, 133 (122), 4160–4163. 10.1021/ja108560e. 21381734 PMC3086499

[ref18] Del Hoyo A. M.; Herraiz A. G.; Suero M. G. A Stereoconvergent Cyclopropanation Reaction of Styrenes. Angew. Chem., Int. Ed. 2017, 56, 1610–1613. 10.1002/anie.201610924. 27981721

[ref19] Herraiz A. G.; Suero M. G. A transition-metal-free & diazo-free styrene cyclopropanation. Chem. Sci. 2019, 10, 9374–9379. 10.1039/C9SC02749A. 32110302 PMC7017872

[ref20] Li P.; Zhao J.; Shi L.; Wang J.; Shi X.; Li F. Iodine-catalyzed diazo activation to access radical reactivity. Nat. Commun. 2018, 9, 1972 10.1038/s41467-018-04331-4. 29773787 PMC5958049

[ref21] Bauld N. L.; Stufflebeme G. W.; Lorenz K. T. Cation radical pericyclic reactions: Cyclopropanation. J. Phys. Org. Chem. 1989, 2, 585–601. 10.1002/poc.610020802.

[ref22] Bauld N. L.; Yueh W. Mechanistic Criteria for the Cation Radical vs Electrophilic Mechanistic Distinction. J. Am. Chem. Soc. 1994, 116, 8845–8846. 10.1021/ja00098a069.

[ref23] Yueh W.; Bauld N. L. Mechanistic Criteria for Cation Radical Reactions: Aminium Salt-Catalyzed Cyclopropanation. J. Am. Chem. Soc. 1995, 117 (21), 5671–5676. 10.1021/ja00126a007.

[ref24] Sarabia F. J.; Ferreira E. M. Radical Cation Cyclopropanations via Chromium Photooxidative Catalysis. Org. Lett. 2017, 19 (11), 2865–2868. 10.1021/acs.orglett.7b01095. 28498677 PMC5985524

[ref25] Cho Y. H.; Kim J. H.; An H.; Ahn K.-H.; Kang E. J. Cycloaddition Reactions of Alkene Radical Cations using Iron(III)-Phenanthroline Complex. Adv. Synth. Catal. 2020, 362 (11), 2183–2188. 10.1002/adsc.202000191.

[ref26] Bauld N. L. Cation radicals in the synthesis and reactions of cyclobutanes. PATAI’s Chemistry of Functional Groups; Wiley and Sons, 2009; 10.1002/9780470682531.

[ref27] Yu Y.; Fu Y.; Zhong F. Benign catalysis with iron: facile assembly of cyclobutanes and cyclohexenes via intermolecular radical cation cycloadditions. Green Chem. 2018, 20, 1743–1747. 10.1039/C8GC00299A.

[ref28] Shida N.; Imada Y.; Nagahara S.; Okada Y.; Chiba K. Interplay of arene radical cations with anions and fluorinated alcohols in hole catalysis. Communications Chemistry 2019, 2, 24 10.1038/s42004-019-0125-4.

[ref29] Horibe T.; Katagiri K.; Ishihara K. Radical-Cation-Induced Crossed [2 + 2] Cycloaddition of Electron-Deficient Anetholes Initiated by Iron(III) Salt. Adv. Synth. Catal. 2020, 362, 960–963. 10.1002/adsc.201901337.

[ref30] Scholz S. O.; Kidd J. B.; Capaldo L.; Flikweert N. E.; Littlefield R. M.; Yoon T. P. Construction of Complex Cyclobutane Building Blocks by Photosensitized [2 + 2] Cycloaddition of Vinyl Boronate Esters. Org. Lett. 2021, 23 (9), 3496–3501. 10.1021/acs.orglett.1c00938. 33844561 PMC8547782

[ref31] Namyslo J. C.; Kaufmann D. E. The Application of Cyclobutane Derivatives in Organic Synthesis. Chem. Rev. 2003, 103 (4), 1485–1538. 10.1021/cr010010y. 12683789

[ref32] Poplata S.; Tröster A.; Zou Y.-Q.; Bach T. Recent Advances in the Synthesis of Cyclobutanes by Olefin [2 + 2] Photocycloaddition Reactions. Chem. Rev. 2016, 116, 9748–9815. 10.1021/acs.chemrev.5b00723. 27018601 PMC5025837

[ref33] Ischay M. A.; Lu Z.; Yoon T. P. [2 + 2] Cycloadditions by Oxidative Visible Light Photocatalysis. J. Am. Chem. Soc. 2010, 132, 8572–8574. 10.1021/ja103934y. 20527886 PMC2892825

[ref34] Ischay M. A.; Ament M. S.; Yoon T. P. Crossed intermolecular [2 + 2] cycloaddition of styrenes by visible light photocatalysis. Chem. Sci. 2012, 3, 2807–2811. 10.1039/c2sc20658g. 22984640 PMC3439822

[ref35] Yoon T. P. Visible Light Photocatalysis: The Development of Photocatalytic Radical Ion Cycloadditions. ACS Catal. 2013, 3, 895–902. 10.1021/cs400088e. 23691491 PMC3656437

[ref36] Tanaka K.; Iwama Y.; Kishimoto M.; Ohtsuka N.; Hoshino Y.; Honda K. Redox Potential Controlled Selective Oxidation of Styrenes for Regio- and Stereoselective Crossed Intermolecular [2 + 2] Cycloaddition via Organophotoredox Catalysis. Org. Lett. 2020, 22 (13), 5207–5211. 10.1021/acs.orglett.0c01852. 32525321

[ref37] Miura T.; Kim S.; Kitano Y.; Tada M.; Chiba K. Electrochemical Enol Ether/Olefin Cross-Metathesis in a Lithium Perchlorate/Nitromethane Electrolyte Solution. Angew. Chem., Int. Ed. 2006, 45, 1461–1463. 10.1002/anie.200503656. 16440381

[ref38] Chiba K.; Miura T.; Kim Sh.; Kitano Y.; Tada M. J. Am. Chem. Soc. 2001, 123 (45), 11314–11315. 10.1021/ja016885b. 11697984

[ref39] A certain amount of product is obtained without adding electrolyte probably because diazo compounds can act as charge carriers due to the existence of ionic equilibrium: N^–^=N^+^=CHCO_2_Et ↔ N≡N^+^–C^–^HCO_2_Et.

[ref40] Bug T.; Hartnagel M.; Schlierf C.; Mayr H. How Nucleophilic Are Diazo Compounds?. Chem.—Eur. J. 2003, 9, 4068–4076. 10.1002/chem.200304913. 12953192

[ref41] When cyclopropanation is performed in a divided cell (H-cell), product F is formed in <10% of yield, which can prove the hypothesis that reduction of H occurs on the cathode (see SI).

