# Supplementary material for: Electrochemical Cycloaddition Reactions of Alkene Radical Cations: A Route toward Cyclopropanes and Cyclobutanes
Source: Org Lett. 2023 Feb 14;25(7):1142–6. doi: 10.1021/acs.orglett.3c00121 (PMC9972478; doi:10.1021/acs.orglett.3c00121)
Supplement: Supplementary file 1 — ol3c00121_si_001.pdf [file ol3c00121_si_001.pdf]

Supporting Information

Electrochemical Cycloaddition Reactions of Alkene Radical Cations – A Route towards Cyclopropanes and Cyclobutanes

Katarzyna Rybicka-Jasińska[a*], Zuzanna Szeptuch[a,b], Hubert Kubiszewski[a] and Agnieszka Kowaluk[a]

a Institute of Organic Chemistry, Polish Academy of Sciences, Kasprzaka 44/52, 01-224 Warsaw, Poland
b Faculty of Chemistry, Warsaw University of Technology, Noakowskiego 3, 00-664 Warsaw, Poland

e-mail: *katarzyna.rybickajasinska@icho.edu.pl

Table of contest:

1. General information **S3**
2. Synthesis of substrates **S4**
3. Radical cyclopropanation of olefins with diazo compounds
– general procedure, optimization studies, scope and limitation studies **S5**
4. Electrochemical radical synthesis of cyclobutanes from olefins and styrenes
– general procedure, optimization studies, scope and limitation studies **S15**
5. Mechanistic consideration **S19**
6. NMR spectra **S23**

1. General Information

Materials

All solvents and commercially available reagents were purchased from Sigma-Aldrich, TCI, or Acros Organics as reagent grade and were used without further purification, unless otherwise stated. Dry solvents were taken from Solvent Purification System (SPS) or purchased from Sigma Aldrich. All deuterated solvents used were purchased from Eurisotop.

General Procedures

All the electrochemical reactions were performed in 5 or 10 mL glassy vials sealed with caps containing a rubber septum. Reactions were monitored by thin layer chromatography (TLC), using 0.20 mm Merck silica plates (60F-254) and visualized using UV-light, potassium permanganate, cerium molybdate, or anisaldehyde stain, with heat as a developing agent. Chromatography columns were performed on Merck silica gel 60 (230-400 mesh). GC yields were calibrated with dodecane as an internal standard. Isolated yields refer to spectroscopically (^{1}H NMR) homogeneous materials.

Instrumentation

NMR spectra were recorded at ambient temperature (unless otherwise stated) on Bruker 400 or 500 MHz and Varian 500 or 600 MHz. Chemical shifts are reported in ppm relative to the tetramethyl silane signal or solvent peak (TMS: 0 ppm for ^{1}H and ^{13}C, $CHCl_3$: 7.26 ppm for ^{1}H and 77.00 ppm for ^{13}C). Multiplicities are given as: singlet (s), doublet (d), triplet (t), quartet (q), pentet (p), multiplet (m), broad singlet (brs), quartet of doublets (qd).
LR and HRMS Low-resolution mass spectra (LRMS) were recorded on an Applied Biosystems API 365 mass spectrometer using electrospray ionization (ESI) technique. High-resolution mass spectra (HRMS) were recorded on Waters SYNAPT G2-S HDMS instrument using electron ionization (EI), electrospray ionization (ESI), or atmospheric-pressure chemical ionization (APCI) with time of flight detector (TOF).
GC-MS analyses were performed using Shimadzu GCMS-QP2010 SE gas chromatograph with FID detector and Zebron ZB 5MSi column.
Electrochemical setup:

ElectraSyn 2.0 IKA *(picture available at the IKA website: https://www.ika.com) with* ***5 mL vials with cups*** *and* ***graphite electrode*** *(commercially available, purchased from IKA):*

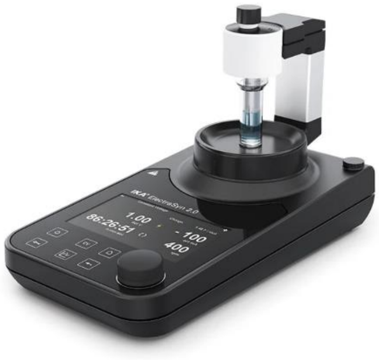
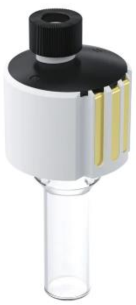
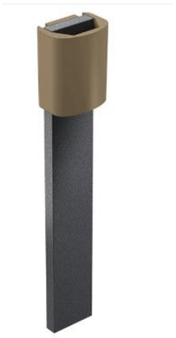

2. Synthesis of substrates

2.1. Alkenes - all the chemical reagents were purchased from commercial sources and used as received.

2.2. Diazo esters and diazo ketones:

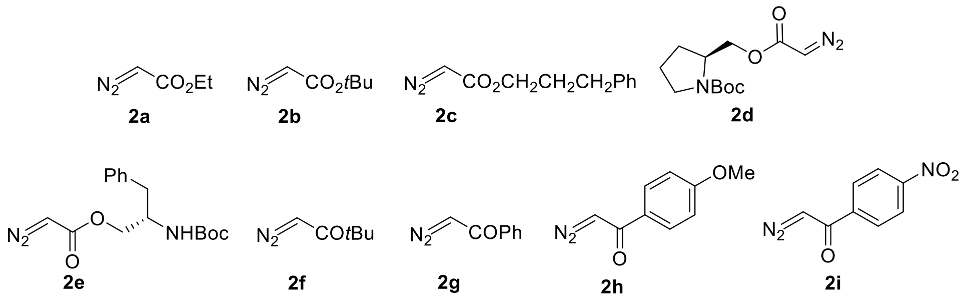

Ethyl diazo acetate (2a) and *tert*-butyl diazoacetate (2b) were purchased from commercial sources and used as received. Compounds 2c[1], 2d[1], 2e[1], 2f[2], 2g[2], 2h[2], 2i[2] are known and their spectroscopic feature is in a good agreement with that reported in the literature.

CAUTION: Although we did not observe any problems during the synthesis of diazo compounds, note that diazo reagents can be explosive and their synthesis must be perform in careful manner.

3. Radical Cyclopropanation of Olefins with Diazo Compounds

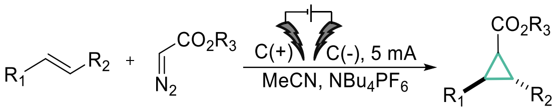

General procedure: A electrolyte (0.5 mmol in 4 mL MeCN, c = 0.125), olefin (if solid) and diazo carbonyl compound (if solid) were placed in a 5 mL vial with a septum and dissolved in dry MeCN (c = 0.125 M) then olefin (if liquid, 0.6 mmol, 1 equiv.) was added. The reaction mixture was then degassed for 10 min with Ar and then diazo carbonyl compound (1.2 equiv.) was added and stirred under constant electrical power (I = 5 mA, in some cases 3 mA) for specific amount of time. As both anode and cathode graphite electrodes were used with rapid altering polarity (1 min). The electrical power was turned off, the reaction mixture was concentrated and subjected to flash column chromatography (hexane/AcOEt).

General procedure for 1.2 mmol scale: A electrolyte (1.2 mmol in 8 mL MeCN, c = 0.125), olefin (if solid) and diazo carbonyl compound (if solid) were placed in a 10 mL vial with a septum and dissolved in dry MeCN (c = 0.125 M) then olefin (if liquid, 1.2 mmol, 1 equiv.) was added. The reaction mixture was then degassed for 10 min with Ar and then diazo carbonyl compound (1.2 equiv.) was added and stirred under constant electrical power (I = 5 mA, in some cases 3 mA) for specific amount of time. As both anode and cathode graphite electrodes were used with rapid altering polarity (1 min). The electrical power was turned off, the reaction mixture was concentrated and subjected to flash column chromatography (hexane/AcOEt).

Optimization studies:

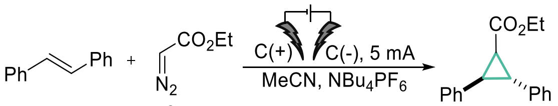

1a **2a** **3**

Table S1. Initial experiments

Entry	I [mA]	Alternation of the polarity	Time [h]	Conversion [%]	Yield [%]*
1	10	-	5	100	41
2	10	1 min	5	100	48
**3**	**5**	**1 min**	**10**	**<100**	**67**
4	-	-	24	0	0
5	5	-	10h	<100	59

Reaction conditions: *trans*-stilbene (**1**, 0.6 mmol, 1.0 equiv.), ethyl diazo acetate (**2**, 1.0 equiv.), MeCN (c = 0.125 M), NBu_4PF_6 (c = 0.125 M), $(+)C_{graphite}/(-)C_{graphite}$, alternation of the polarity (ElectraSyn 2.0 IKA, 1 min), rt. *Isolated yields

Table S2. Optimization of a solvent.

Entry	Solvent	Yield [%]*
1	MeCN	67
2	$MeCN_{dry}$	70
**3**	**$MeCN_{dry}$**	**70**
4	$MeOH_{dry}$	0
5	DMF_{dry}	0
6	THF_{dry}	40
**7**	**DCM_{dry}**	**71**
**8**	**DCE_{dry}**	**72**

Reaction conditions: *trans*-stilbene (**1**, 0.6 mmol, 1.0 equiv.), ethyl diazo acetate (**2**, 1.0 equiv.), solvent (c = 0.125 M), NBu_4PF_6 (c = 0.125 M), (+)$C_{graphite}$/(-)$C_{graphite}$, I = 5 mA, alternation of the polarity (ElectraSyn 2.0 IKA, 1 min), rt, 10h. *Isolated yields

Table S3. Electrolyte concentration

Entry	Electrolyte Concentration [M]	Yield [%]*
1	0.156	71
**2**	**0.125**	**70**
3	0.094	72
4	0	38

Reaction conditions: *trans*-stilbene (**1**, 0.6 mmol, 1.0 equiv.), ethyl diazo acetate (**2**, 1.0 equiv.), solvent (c = 0.125 M), NBu_4PF_6 (c = xx M), (+)$C_{graphite}$/(-)$C_{graphite}$, I = 5 mA, alternation of the polarity (ElectraSyn 2.0 IKA, 1 min), rt, 10h. *Isolated yields

Table S4. Optimization of the substrates' ratio.

Entry	alkene : ethyl diazoacetate	Yield [%]*
1	1.0 : 1.0	70
**2**	**1.0 : 1.2**	**74**
3	1.0 : 1.5	75
4	1.0 : 2.0	75
5	1.2 : 1.0	60

Reaction conditions: *trans*-stilbene (**1**, xx equiv.), ethyl diazo acetate (**2**, xx equiv.), solvent (c = 0.125 M), NBu_4PF_6 (c = 0.125 M), (+)$C_{graphite}$/(-)$C_{graphite}$, I = 5 mA, alternation of the polarity (ElectraSyn 2.0 IKA, 1 min), rt, 10 h. *Isolated yields

Table S5. Solvent concentration

Entry	Solvent Concentration [M]	Yield [%]*
1	0.1	71
2	0.125	74
**3**	**0.15**	**78**
4	0.175	80

Reaction conditions: *trans*-stilbene (**1**, 0.6 mmol, 1.0 equiv.), ethyl diazo acetate (**2**, 1.2 equiv.), solvent (c = xx M), NBu_4PF_6 (c = 0.125 M), (+)$C_{graphite}$/(-)$C_{graphite}$, I = 5 mA, alternation of the polarity (ElectraSyn 2.0 IKA, 1 min), rt, 10 h. *Isolated yields

Table S6. Electrolyte

Entry	Electrolyte	Yield [%]*
**1**	**$LiCF_3SO_2$**	**75**
2	$LiBF_4$	47
**3**	**NBu_4PF_6**	**78/80**
4	NBu_4BF_4	75
**5**	NBu_4ClO_4	38
**6**	$NBu_4CF_3SO_2$	72

Reaction conditions: *trans*-stilbene (**1**, 0.6 mmol, 1.0 equiv.), ethyl diazoacetate (**2**, 1.2 equiv.), solvent (c = 0.15 M), electrolyte (c = 0.125 M), (+)$C_{graphite}$/(-)$C_{graphite}$, I = 5 mA, alternation of the polarity (ElectraSyn 2.0 IKA, 1 min), rt, 10h. *Isolated yields

Scope and limitations studies:

Ethyl 2,3-diphenylcyclopropane-1-carboxylate (**3**)

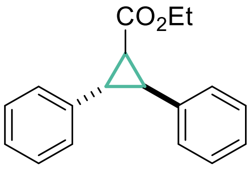

Following the general procedure, compound **3** was obtained from *trans*-stilbene (0.6 mmol) and ethyl diazoacetate (0.72 mmol). Reaction was performed for 10 h, with the I = 5 mA. The crude product was purified by column chromatography to using silica gel (hexane/AcOEt) afford 127 mg of compound **3** (colorless oil, yield = **80%**). *For the reaction performed for 1.2 mmol scale: yield = 74% (236 mg), 20h.* Analytical data for compound **3** are in agreement with the literature data.[3]

^{1}H NMR (500 MHz, $CDCl_3$) δ 7.65 – 6.90 (m, 10H), 4.00 (qd, *J* = 7.1, 1.6 Hz, 2H), 3.28 (dd, *J* = 7.1, 5.2 Hz, 1H), 2.97 (dd, *J* = 9.6, 7.0 Hz, 1H), 2.47 (dd, *J* = 9.6, 5.2 Hz, 1H), 1.08 (t, *J* = 7.1 Hz, 3H) ppm.

^{13}C{H} NMR (126 MHz, $CDCl_3$) δ 169.9, 139.6, 136.1, 129.1, 128.54, 128.53, 128.0, 126.8, 126.61, 126.58, 60.4, 34.3, 31.2, 29.2, 14.0 ppm.

HRMS (EI): *m/z* calcd for $C_{18}H_{18}O_2Na$: 289.1204 [*M+Na*]; found 289.1206.

Ethyl 2,3-di-*p*-tolylcyclopropane-1-carboxylate (**4**)

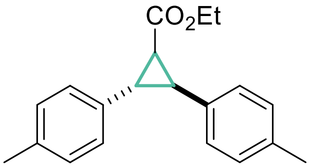

Following the general procedure, compound **4** was obtained from (*E*)-1,2-di-p-tolylethene (0.6 mmol) and ethyl diazoacetate (0.72 mmol). Reaction was performed for 10 h, with the I = 5 mA. The crude product was purified by column chromatography to using silica gel (hexane/AcOEt) afford 97 mg of compound **4** (colorless oil, yield = **55%**). Analytical data for compound **4** are in agreement with the literature data.[3]

^{1}H NMR (500 MHz, $CDCl_3$) δ 7.22 (d, *J* = 7.9 Hz, 2H), 7.13 (s, 4H), 7.09 (d, *J* = 7.8 Hz, 2H), 4.04 – 3.87 (m, 2H), 3.15 (dd, *J* = 7.0, 5.2 Hz, 1H), 2.85 (dd, *J* = 9.5, 7.1 Hz, 1H), 2.36 (s, 1H), 2.33 (s, 3H), 2.31 (s, 3H), 1.06 (t, *J* = 7.1 Hz, 3H) ppm.

^{13}C{H} NMR (126 MHz, $CDCl_3$) δ 170.2, 136.7, 136.4, 136.3, 133.1, 129.2, 129.0, 128.8, 126.6, 60.4, 34.1, 31.1, 29.1, 21.1, 21.0, 14.1 ppm.

Ethyl 2,3-bis(4-bromophenyl)cyclopropane-1-carboxylate (**5**)

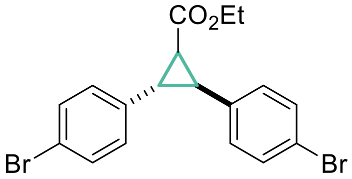

Following the general procedure, compound **5** was obtained from (*E*)-1,2-bis(4-bromophenyl)ethene (0.4 mmol) and ethyl diazoacetate (1.2 equiv. mmol). Reaction was performed for 15 h, with the I = 5 mA Because of low solubility of substrate in MeCN instead of MeCN DCE was used. The concentration was also changed to: 0.1 M. The crude product was purified by column chromatography to using silica gel (hexane/AcOEt) afford 101 mg of compound **5** (white solid, yield = **60%**). Analytical data for compound **5** are in agreement with the literature data.[3]

^{1}H NMR (500 MHz, $CDCl_3$) δ 7.43 (m, 4H), 7.19 (d, *J* = 8.2 Hz, 2H), 7.09 (d, *J* = 8.3 Hz, 2H), 3.98 (qd, *J* = 7.1, 1.6 Hz, 2H), 3.12 (dd, *J* = 7.0, 5.2 Hz, 1H), 2.80 (dd, *J* = 9.6, 7.0 Hz, 1H), 2.38 (dd, *J* = 9.6, 5.2 Hz, 1H), 1.08 (t, *J* = 7.1 Hz, 3H) ppm.

^{13}C{H} NMR (126 MHz, $CDCl_3$) δ 169.4, 138.2, 134.7, 131.7, 131.2, 130.8, 128.3, 121.0, 120.6, 60.7, 33.6, 31.1, 28.8, 14.1 ppm.

Ethyl 2,3-bis(4-fluorophenyl)cyclopropane-1-carboxylate (**6**)

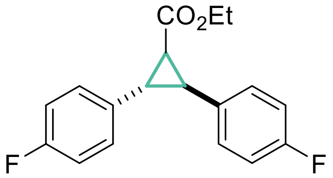

Following the general procedure, compound **6** was obtained from (*E*)-1,2-bis(4-fluorophenyl)ethene (0.6 mmol) and ethyl diazoacetate (0.72 mmol). Reaction was performed for 15 h, with the I = 5 mA. Because of low solubility of substrate in MeCN instead of MeCN, DCE was used. The crude product was purified by column chromatography to using silica gel (hexane/AcOEt) afford 112 mg of compound **6** (colorless oil, yield = **62%**). Analytical data for compound **6** are in agreement with the literature data.[4]
^{1}H NMR (400 MHz, $CDCl_3$) δ 7.33 – 7.27 (m, 2H), 7.24 – 7.16 (m, 2H), 7.06 – 6.94 (m, 4H), 3.97 (q, *J* = 6.88 Hz, 2H), 3.16 (dd, *J* = 7.0, 5.2 Hz, 1H), 2.83 (ddd, *J* = 9.5, 7.0, 1.0 Hz, 1H), 2.35 (dd, *J* = 9.6, 5.2 Hz, 1H), 1.07 (t, *J* = 7.1 Hz, 3H) ppm.
^{13}C{H} NMR (101 MHz, $CDCl_3$) δ 169.7, 163.1, 163.0, 160.7, 160. 6, 135.0, 134.9, 131.61, 131.58, 130.65, 130.57, 128.3, 128.2, 115.6, 115.4, 115.1, 114.9, 60.6, 33.4, 31.1, 28.8, 14.1 ppm.

Ethyl 2,3-bis(4-nitrophenyl)cyclopropane-1-carboxylate (**7**)

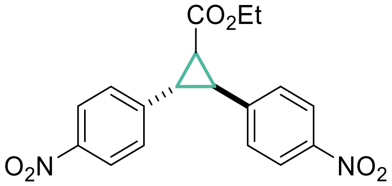

Because of low solubility of substrate in MeCN, DCE, DCM, product **7** was not obtained, yield = 0%.

Ethyl 2-(4-nitrophenyl)-3-phenylcyclopropane-1-carboxylate (**8**) (mixture of two diastereoisomers 1:1)

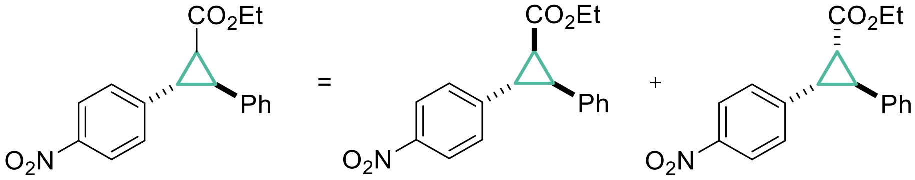

1,2-*anti*-**8** 1,2-*syn*-**8**

Following the general procedure, compound **8** was obtained from (*E*)-1-nitro-4-styrylbenzene (0.6 mmol) and 3-phenylpropyl 2-diazoacetate (0.72 mmol). Reaction was performed for 12 h, with the I = 5 mA. The crude product was purified by column chromatography to using silica gel (hexane/AcOEt) afford 109 mg (sum of two diastereoisomers) of compound **8** as two diastereoisomers (1:1) (both yellow oils, yield = **58%**).
1,2-*anti*-8:
^{1}H NMR (500 MHz, $CDCl_3$) δ 8.16 (d, *J* = 8.5 Hz, 2H), 7.51 (d, *J* = 8.4 Hz, 2H), 7.35 (m, 2H), 7.26 (m, 3H), 3.99 (m, 2H), 3.26 (dd, *J* = 7.1, 5.3 Hz, 1H), 2.95 (dd, *J* = 9.6, 7.1 Hz, 1H), 2.52 (dd, *J* = 9.6, 5.3 Hz, 1H), 1.10 (t, *J* = 7.1 Hz, 3H) ppm.
^{13}C{H} NMR (126 MHz, $CDCl_3$) δ 169.4, 146.9, 143.9, 138.4, 130.0, 128.7, 127.1, 126.6, 123.3, 60.9, 33.7, 31.5, 29.9, 14.1 ppm.
HRMS (EI): *m/z* calcd for $C_{18}H_{16}NO_4$: 310.1081 [*M-H*]; found 310.1079.
IR (cm^{-1}): 2982, 1727, 1601, 1519, 1346, 1181.
1,2-*syn*-8:
^{1}H NMR (500 MHz, $CDCl_3$) δ 8.25 – 8.11 (m, 2H), 7.43 – 7.37 (m, 2H), 7.34 – 7.29 (m, 4H), 7.26 (m, 1H), 3.98 (q, *J* = 7.1 Hz, 2H), 3.30 (dd, *J* = 6.9, 5.2 Hz, 1H), 3.04 – 2.92 (m, 1H), 2.52 (dd, *J* = 9.8, 5.2 Hz, 1H), 1.05 (t, *J* = 7.1 Hz, 3H) ppm.
^{13}C{H} NMR (126 MHz, $CDCl_3$) δ 169.0, 147.5, 146.7, 135.1, 128.9, 128.2, 127.3, 127.2, 123.9, 60.8, 35.1, 31.9, 28.8, 13.9 ppm.
HRMS (EI): *m/z* calcd for $C_{18}H_{16}NO_4$: 310.1081 [*M-H*]; found 310.1079.

IR (cm^{-1}): 2982, 1728, 1601, 1519, 1346, 1181.

Ethyl 4-(2-(ethoxycarbonyl)-3-phenylcyclopropyl)benzoate (**9**) ((mixture of *syn* and *anti* isomers (1:1))

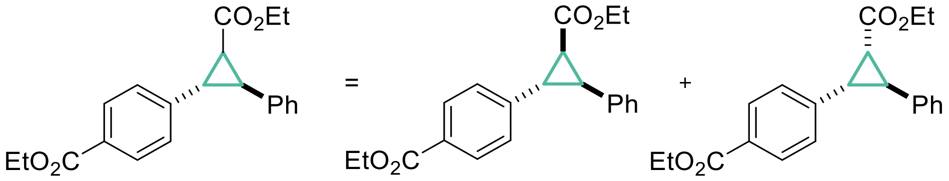

1,2-*anti*-**9** 1,2-*syn*-**9**

Following the general procedure, compound **9** was obtained from *ethyl (E)-4-styrylbenzoate* (0.6 mmol) and ethyl diazoacetate (0.72 mmol). Reaction was performed for 12 h, with the I = 5 mA The crude product was purified by column chromatography to using silica gel (hexane/AcOEt) afford 122 mg of compound **9** as a mixture of two diastereoisomers (colorless oil, yield = **60%**).
^{1}H NMR (500 MHz, $CDCl_3$) *syn* and *anti*: δ 8.00 (d, *J* = 8.4 Hz, 2H), 7.38 – 7.12 (m, 7H), 4.38 (q, *J* = 7.1 Hz, 2H), 3.96 (q, *J* = 7.1 Hz, 2H), 3.25 (dd, *J* = 6.9, 5.2 Hz, 1H), 3.07 – 2.80 (m, 1H), 2.47 (dd, *J* = 9.7, 5.2 Hz, 1H), 1.39 (t, *J* = 7.1 Hz, 3H), 1.04 (t, *J* = 7.1 Hz, 3H) ppm.
^{13}C{H} NMR (126 MHz, $CDCl_3$) *syn* and *anti*: δ 169.5, 169.4, 166.3, 166.2, 144.9, 141.3, 138.9, 135.6, 129.8, 129.3, 129.1, 128.97, 128.85, 128.6, 128.0, 126.9, 126.8, 126.5, 126.3, 60.8, 60.7, 60.52, 60.51, 34.7, 34.1, 31.6, 31.3, 29.4, 29.0, 14.2, 14.0, 13.9 ppm.
HRMS (EI): *m/z* calcd for $C_{21}H_{22}O_4Na$: 361.1416 [*M+Na*]; found 361.1413.
IR (cm^{-1}): 2981, 1778, 1277, 1179, 1105, 699.

Ethyl 2-(4-bromophenyl)-3-phenylcyclopropane-1-carboxylate (**10**) ((mixture of *syn* and *anti* isomers (1:1))

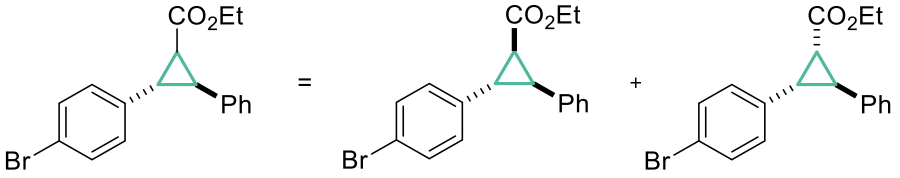

1,2-*anti*-**10** 1,2-*syn*-**10**

Following the general procedure, compound **10** was obtained from *(E)-1,2-bis(4-bromophenyl)ethene* (0.6 mmol) and ethyl diazoacetate (0.72 mmol). Reaction was performed for 12 h, with the I = 5 mA The crude product was purified by column chromatography to using silica gel (hexane/AcOEt) afford 132 mg of compound **10** as a mixture of two diastereoisomers (1:1) (colorless oil, yield = **64%**).
^{1}H NMR (500 MHz, $CDCl_3$) *syn* and *anti*: δ 7.45 (m, 2H + 2H), 7.37 – 7.28 (m, 3H + 3H), 7.29 – 7.18 (m, 3H + 3H), 7.13 (m, 1H + 1H), 4.05 – 3.91 (m, 2H + 2H), 3.19 (m, 1H + 1H), 2.92 – 2.80 (m, 1H + 1H), 2.44 (dd, J = 9.6 Hz, J = 5.6 Hz, 1H) 2.40 (dd, J = 9.6 Hz, J = 5.6 Hz, 1H), 1.10 (t, *J* = 7.1 Hz, 3H), 1.05 (t, *J* = 7.1 Hz, 3H) ppm.
^{13}C{H} NMR (126 MHz, $CDCl_3$) *syn* and *anti*: δ 169.7, 169.6, 139.2, 138.7, 135.8, 135.2, 131.7, 131.2, 130.9, 129.2, 129.1, 128.7, 128.6, 128.4, 128.2, 128.1, 127.1, 126.9, 126. 7, 126.6, 120.8, 120.4, 60.64, 60.59, 34.4, 33.7, 31.24, 31.21, 29.5, 28.6, 14.2, 14.1 ppm.
HRMS (EI): *m/z* calcd for $C_{18}H_{17}O_2BrNa$: 367.0310 [*M+Na*]; found 367.0311.
IR (cm^{-1}): 3028, 2980, 1727, 1492, 1179.

Ethyl 2-(4-chlorophenyl)-3-phenylcyclopropane-1-carboxylate (**11**) (mixture of *syn* and *anti* isomers (1:1))

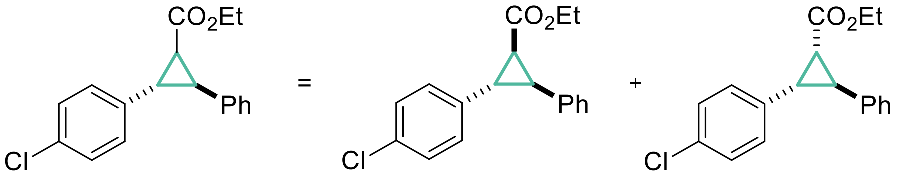

1,2-*anti*-**11** 1,2-*syn*-**11**

Following the general procedure, compound **11** was obtained from *(E)-1,2-bis(4-chlorophenyl)ethene* (0.6 mmol) and ethyl diazoacetate (0.72 mmol). Reaction was performed for 15 h, with the I = 5 mA The crude product was purified by column chromatography to using silica gel (hexane/AcOEt) afford 90 mg of compound **11** as a mixture of two diastereoisomers (1:1) (colorless oil, yield = **50%**). Analytical data for compound **11** are in agreement with the literature data.[5]

^{1}H NMR (400 MHz, $CDCl_3$) *syn* and *anti*: 7.39 – 7.16 (m, 9H), 4.08 – 3.86 (m, 2H), 3.18 (dd, *J* = 6.8, 5.4 Hz, 1H), 2.87 (dd, *J* = 9.5, 6.8 Hz, 1H), 2.41 (ddd, *J* = 14.7, 9.6, 5.2 Hz, 1H), 1.04 (2 x t, 3H) ppm.

^{13}C{H} NMR (100 MHz, $CDCl_3$) *syn* and *anti*: δ 169.8, 169.7, 139.2, 138.2, 135.8, 134.7, 132.7, 132.4, 130.5, 129.1, 128.72, 128.66, 128.3, 128.13, 128.07, 127.0, 126.9, 126.6, 60.62, 60.58, 34.4, 33.7, 31.25, 31.33, 29.5, 28.6, 14.1, 14.0 ppm.

Ethyl 2-phenyl-3-(*p*-tolyl)cyclopropane-1-carboxylate (**12**) (mixture of *syn* and *anti* isomers (1:1.2))

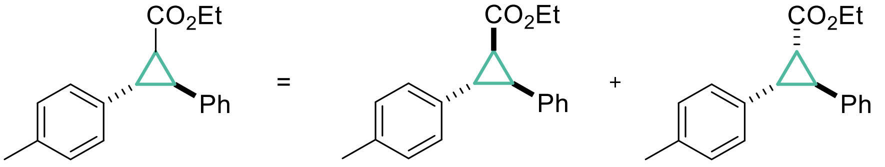

1,2-*anti*-**12** 1,2-*syn*-**12**

Following the general procedure, compound **12** was obtained from (*E*)-1-methyl-4-styrylbenzene (0.6 mmol) and ethyl diazoacetate (0.72 mmol). Reaction was performed for 10 h, with the I = 5 mA The crude product was purified by column chromatography to using silica gel (hexane/AcOEt) afford 117 mg of compound **12** as a mixture of two diastereoisomers (1:1) (colorless oil, yield = **70%**). Analytical data for compound **12** are in agreement with the literature data.[3]

^{1}H NMR (400 MHz, $CDCl_3$) *syn* and *anti*: 7.38 – 7.28 (m, 2H), 7.28 – 7.21 (m, 4H + 4H), 7.16 (s, 2H + 2H), 7.14 – 7.08 (m, 1H + 1H), 3.98 (m, 2H + 2H), 3.21 (m, 1H + 1H), 2.91 (m, 1H + 1H), 2.40 (m, 1H + 1H), 2.35 (s, 3H), 2.34 (s, 3H), 1.09 (t, 3H), 1.05 (t, 3H) ppm.

^{13}C{H} NMR (101 MHz, $CDCl_3$) *syn* and *anti*: δ 170.1, 139.8, 136.6, 136.4, 136.32, 136.27, 133.0, 129.3, 129.2, 129.0, 128.8, 128.63, 128.59, 128.1, 126.9, 126.7, 126.63, 126.60, 60.5, 60.4, 34.3, 34.2, 31.20, 31.16, 29.4, 29.0, 21.1, 21.0, 14.12, 14.07 ppm.

Ethyl 2-(4-methoxyphenyl)-3-methylcyclopropane-1-carboxylate (**13**) (mixture of *syn* and *anti* isomers (1:1))

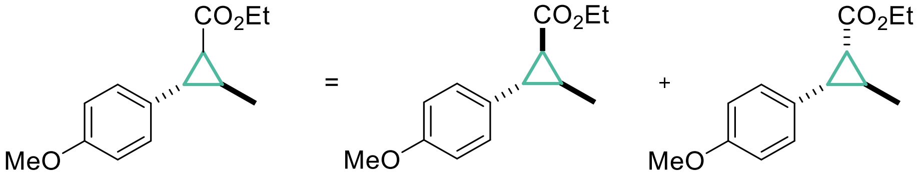

1,2-*anti*-**13** 1,2-*syn*-**13**

Following the general procedure, compound **13** was obtained from (*E*)-1-methoxy-4-(prop-1-en-1-yl)benzene (0.6 mmol) and ethyl diazoacetate (0.72 mmol). Reaction was performed for 10 h, with the I = 5 mA The crude product was purified by column chromatography to using silica gel (hexane/AcOEt) afford 85 mg (sum of two diastereoisomers) of compound **13** as a two diastereoisomers (1:1) (both colorless oil, yield = **61%**). Analytical data for compound **13** are in agreement with the literature data.[4]

1,2-syn-13:

^{1}H NMR (400 MHz, $CDCl_3$) δ 7.21 – 7.05 (m, 2H), 6.88 – 6.72 (m, 2H), 3.89 (qd, *J* = 7.1, 0.8 Hz, 2H), 3.77 (s, 3H), 2.28 (dd, *J* = 9.2, 6.8 Hz, 1H), 2.01 (ddd, *J* = 6.8, 6.0, 5.0 Hz, 1H), 1.76 (dd, *J* = 9.2, 5.1 Hz, 1H), 1.25 (d, *J* = 6.0 Hz, 3H), 1.02 (t, *J* = 7.1 Hz, 3H) ppm.

^{13}C{H} NMR (100 MHz, $CDCl_3$) δ 170.9, 158.3, 130.1, 128.8, 113.3, 60.0, 55.2, 33.6, 29.9, 19.8, 17.7, 14.1 ppm

1,2-anti-13:

^{1}H NMR (400 MHz, $CDCl_3$) δ 7.09 – 6.92 (m, 2H), 6.87 – 6.77 (m, 2H), 4.17 (qd, *J* = 7.1, 1.5 Hz, 2H), 3.77 (s, 3H), 2.36 (dd, *J* = 6.7, 5.0 Hz, 1H), 1.93 (dd, *J* = 9.2, 4.9 Hz, 1H), 1.65 – 1.57 (m, 1H), 1.34 (d, *J* = 6.2 Hz, 3H), 1.28 (t, *J* = 7.1 Hz, 3H) ppm.

^{13}C{H} NMR (100 MHz, $CDCl_3$) δ 171.8, 158.2, 132.6, 127.2, 113.9, 60.4, 55.3, 31.8, 29.1, 25.1, 14.4, 11.9 ppm.

Ethyl 2-methyl-3-phenylcyclopropane-1-carboxylate (**14**) (mixture of two diastereoisomers 1.67 : 1.23)

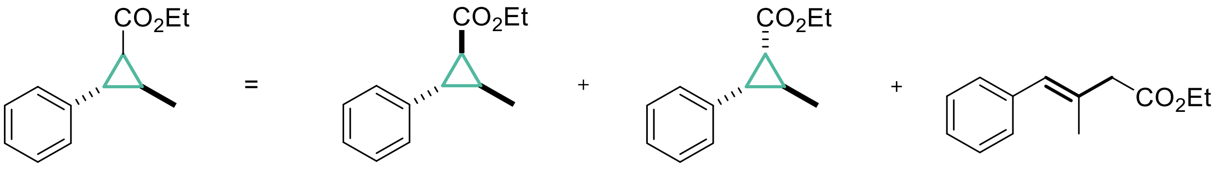

1,2-*anti*-**14** 1,2-*syn*-**14** proton-migrated by-product

Following the general procedure, compound **14** was obtained from *(E)-prop-1-en-1-ylbenzene* (0.6 mmol) and ethyl diazoacetate (0.72 mmol). Reaction was performed for 10 h, with the I = 5 mA and $LiSO_2CF_3$ as a electrolyte. The crude product was purified by column chromatography to using silica gel (hexane/AcOEt) afford 88 mg (sum of two diastereoisomers) of compound **14** as a two diastereoisomers (1:1) (colorless oil, NMR yield = **72**%, NMR yield for proton-migrated by-product: 14%). *For the reaction performed for 1.2 mmol scale: yield = 70% (171 mg), 30h.* Analytical data for compound **14** are in agreement with the literature data.[6]

1,2-syn-14:

^{1}H NMR (500 MHz, $CDCl_3$) δ 7.26 – 7.21 (m, 4H), 7.19 – 7.14 (m, 1H), 3.86 (qd, *J* = 7.1, 1.1 Hz, 2H), 2.38 – 2.26 (m, 1H), 2.06 (ddd, *J* = 6.8, 5.9, 4.9 Hz, 1H), 1.80 (dd, *J* = 9.3, 5.1 Hz, 1H), 1.26 (d, *J* = 6.1 Hz, 3H), 0.97 (t, *J* = 7.1 Hz, 3H) ppm.

^{13}C{H} NMR (126 MHz, $CDCl_3$) δ 170.9, 136.8, 129.1, 127.8, 126.5, 60.1, 34.2, 30.1, 19.6, 17.7, 14.0 ppm.

***1,2-anti-14* + proton-migrated by-product (inseparable via flash column chromatography)**

^{1}H NMR (500 MHz, $CDCl_3$) δ 7.39 – 7.13 (m, 5H *+ proton migrated by-product*), *6.39 (s, 1H, from proton migrated by-product), 4.18 (q, J = 7.1 Hz, 2H from proton migrated by-product)*, 3.87 (q, J = 7.6 Hz, 2H), *3.17 (d, J = 1.2 Hz, 1H from proton migrated by-product)*, 2.34 (dd, *J* = 9.3, 6.9 Hz, 1H), 2.07 (dd, *J* = 6.0, 0.9 Hz, 1H), *1. 94 (d, J = 1.2 Hz, 1H from proton migrated by-product)*, 1.81 (dd, *J* = 9.3, 5.1 Hz, 1H), *1.29 (d, J = 6.8 Hz, 3H from proton migrated by-product)*, 1.27 (d, *J* = 6.4 Hz, 3H), 0.98 (t, *J* = 7.1 Hz, 3H) ppm.

^{13}C{H} NMR (101 MHz, $CDCl_3$) δ 170.84, 170.81, 136.8, 136.7, 129.1, 128.9, 128.1, 127.8, 126.5, 126.4, 60.6, 60.1, 34.2, 34.1, 30.1, 30.1, 19.64, 19.60, 17.73, 17.69, 14.2, 14.0 ppm.

Ethyl 1a,2,3,7b-tetrahydro-1H-cyclopropa[a]naphthalene-1-carboxylate (**15**) (mixture of two diastereoisomers 1:1 + proton-migrated by-product)

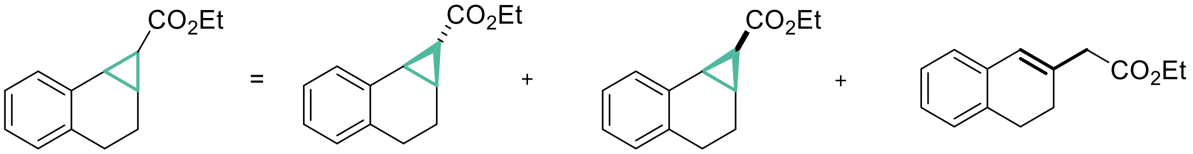

1,2-*anti*-**15** 1,2-*syn*-**15** proton-migrated by-product

Following the general procedure, compound **15** was obtained from *1,2-dihydronaphthalene* (0.6 mmol) and ethyl diazoacetate (0.72 mmol). Reaction was performed for 10 h, with the I = 5 mA and NBu_4PF_6 as a electrolyte. The crude product was purified by column chromatography to using silica gel (hexane/AcOEt) afford 52 mg of compound **15** as a mixture of two diastereoisomers (1:1) (colorless oil, yield = **40%,** NMR yield for proton-migrated by-product: 23%). Analytical data for compound **15** are in agreement with the literature data.[7]

1,2-syn-15:

^{1}H NMR (400 MHz, $CDCl_3$) δ 7.29 – 7.22 (m, 1H), 7.17 – 7.04 (m, 3H), 3.93 (qd, *J* = 7.1, 0.7 Hz, 2H), 2.85 – 2.70 (m, 1H), 2.55 (dt, *J* = 15.6, 5.5 Hz, 1H), 2.41 (t, *J* = 8.7 Hz, 1H), 2.22 – 2.08 (m, 2H), 2.01 (t, *J* = 8.8 Hz, 1H), 1.94 (ddd, *J* = 8.7, 7.5, 4.9 Hz, 1H), 1.05 (t, *J* = 7.2 Hz, 3H) ppm.

^{13}C{H} NMR (101 MHz, $CDCl_3$) δ 170.4, 138.1, 132.6, 129.7, 127.8, 126.2, 125.9, 59.9, 27.9, 26.3, 20.7, 18.7, 18.2, 14.0 ppm.

1,2-anti-14 + proton-migrated by-product (inseparable via flash column chromatography)

^{1}H NMR (400 MHz, $CDCl_3$) δ 7.33 – 7.27 (m, 1H *+ proton migrated by-product)*), 7.19 – 7.08 (m, 2H *+ proton migrated by-product*)), 7.06 – 6.98 (m, 1H *+ proton migrated by-product)*), *6.36 (brs, 1H from proton migrated by-product*) 4.18 (m, 2H *+ proton migrated by-product)*, 3.22 (brs, 1H *from proton migrated by-product)*, 2.86 (t, *J* = 8.2 Hz, 1H *from proton migrated by-product*), 2.73 – 2.62 (m, 1H), 2.57 (dd, *J* = 9.0, 3.5 Hz, 1H), 2.49 (dd, *J* = 13.6, 6.2 Hz, 1H), *2.37 (ddd, J = 9.0, 7.6, 1.0 Hz, 1H)*, 2.25 – 2.14 (m, 2H), 2.11 – 2.03 (m, 1H), 1.88 – 1.75 (m, 1H), 1.28 (td, *J* = 7.1, 0.7 Hz, 3H *+ proton migrated by-product*) ppm.

^{13}C{H} NMR (101 MHz, $CDCl_3$) δ 173.2, 171.1, 135.1, 134.5, 134.2, 133.9, 133.8, 128.8, 128.6, 127.2, 126.8, 126.4, 126.3, 126.1, 125.9, 125.9, 60.7, 60.6, 43.0, 28.0, 27.2, 26.4, 25.4, 24.3, 23.2, 18.6, 14.3, 14.2 ppm.

Ethyl 2,2,3-trimethyl-3-phenylcyclopropane-1-carboxylate (**16**)

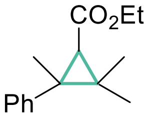

Product **16** was not obtained, yield = 0%, probably due to high steric hindrance.

***Tert*-butyl 2,3-diphenylcyclopropane-1-carboxylate** (**17**)

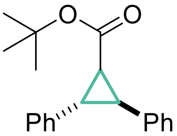

Following the general procedure, compound **17** was obtained from *trans*-stilbene (0.6 mmol) and *tert*-butyl diazoacetate (0.72 mmol). Reaction was performed for 10 h, with the I = 5 mA. The crude product was purified by column chromatography to using silica gel (hexane/AcOEt) afford 128 mg of compound **17** (white solid, yield =**73%**). Analytical data for compound **17** are in agreement with the literature data.[8]

^{1}H NMR (500 MHz, $CDCl_3$) δ 7.47 – 7.18 (m, 10H), 3.20 (dd, *J* = 6.9, 5.1 Hz, 1H), 2.91 (dd, *J* = 9.7, 6.9 Hz, 1H), 2.39 (dd, *J* = 9.7, 5.2 Hz, 1H), 1.26 (s, 9H) ppm.

^{13}C{H} NMR (126 MHz, $CDCl_3$) δ 169.0, 139.9, 136.4, 129.3, 128.5, 127.9, 126.7, 126.6, 126.5, 80.6, 34.2, 32.2, 28.6, 27.8 ppm.

HRMS (EI): *m/z* calcd for $C_{20}H_{22}O_2Na$: 317.1517 [*M+Na*]; found 317.1520.

3-phenylpropyl (2R,3R)-2,3-diphenylcyclopropane-1-carboxylate (**18**)

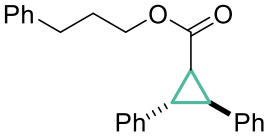

Following the general procedure, compound **18** was obtained from *trans*-stilbene (0.6 mmol) and 3-phenylpropyl 2-diazoacetate (0.72 mmol). Reaction was performed for 12 h, with the I = 5 mA. The crude product was purified by column chromatography to using silica gel (hexane/AcOEt) afford 150 mg of compound **18** (colorless, yield = **70%**).

^{1}H NMR (500 MHz, $CDCl_3$) δ 7.44 – 7.10 (m, 15H), 3.96 (dt, *J* = 9.0, 6.5 Hz, 2H), 3.27 (dd, *J* = 7.1, 5.2 Hz, 1H), 2.98 (dd, *J* = 9.6, 7.0 Hz, 1H), 2.54 (dd, *J* = 8.7, 6.7 Hz, 2H), 2.48 (dd, *J* = 9.6, 5.2 Hz, 1H), 1.83 – 1.69 (m, 2H) ppm.

^{13}C{H} NMR (126 MHz, $CDCl_3$) δ 169.9, 141.2, 139.5, 136.1, 129.1, 128.6, 128.3, 128.1, 126.9, 126.7, 126.6, 125.9, 63.9, 34.4, 31.9, 31.3, 30.0, 29.2 ppm.

HRMS (EI): *m/z* calcd for $C_{25}H_{24}O_2Na$: 379.1674 [*M+Na*]; found 379.1675.

IR (cm^{-1}): 3027, 2952, 1728, 1496, 1171, 748, 698.

tert-Butyl-2,3-diphenylcyclopropane-1-carbonyl)oxy)methyl)pyrrolidine-1-carboxylate (**19**)

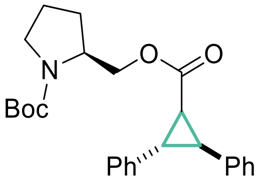

Following the general procedure, compound **19** was obtained from *trans*-stilbene (0.6 mmol) and *tert*-butyl (*S*)-2-((2-diazoacetoxy)methyl)pyrrolidine-1-carboxylate (0.72 mmol). Reaction was performed for 12 h, with the I = 5 mA. The crude product was purified by column chromatography to using silica gel (hexane/AcOEt) afford 202 mg of compound **19** (white foam, yield = **80%**).

^{1}H NMR (400 MHz, $CDCl_3$) δ 7.39 – 7.17 (m, 10H), 4.12 (m, 1H), 4.03 (m, 1H), 3.90 (m, 1H), 3.72 (m, 1H), 3.43 – 3.17 (m, 3H), 2.94 (dd, *J* = 9.7, 7.0 Hz, 1H), 2.43 (dd, *J* = 9.6, 5.3 Hz, 1H), 1.82 – 1.66 (m, 3H), 1.43 (s, 9H) ppm.

^{13}C{H} NMR (101 MHz, $CDCl_3$) δ 169.7, 139.5, 136.1, 129.1, 128.6, 128.2, 126.9, 126.8, 126.7, 126.5, 64.7, 60.4, 55.5, 46.4, 34.5, 31.3, 29.4, 28.5, 23.0, 14.2 ppm.

HRMS (EI): *m/z* calcd for $C_{26}H_{31}NO_4Na$: 444.2152 [*M+Na*]; found 444.2151.

IR (cm^{-1}): 2975, 1732, 1694, 1393, 1166.

2-((_tert_-butoxycarbonyl)amino)-3-phenylpropyl-2,3-diphenylcyclopropane-1-carboxylate (**20**)

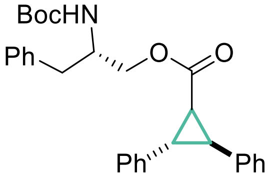

Following the general procedure, compound **20** was obtained from *trans*-stilbene (0.6 mmol) and (*S*)-2-((*tert*-butoxycarbonyl)amino)-3-phenylpropyl 2-diazoacetate (0.72 mmol). Reaction was performed for 12 h, with the I = 5 mA. The crude product was purified by column chromatography to using silica gel (hexane/AcOEt) afford 183 mg of compound **20** (colorless oil, yield = **65%**).

^{1}H NMR (500 MHz, $CDCl_3$) δ 7.46 – 7.35 (m, 6H), 7.33 – 7.24 (m, 8H), 7.09 (s, 1H), 4.31 (s, 1H), 3.99 – 3.79 (m, 3H), 3.28 (dd, *J* = 7.1, 5.2 Hz, 1H), 3.04 (dd, *J* = 9.6, 7.2 Hz, 1H), 2.69 (s, 1H), 2.57 (dd, *J* = 13.6, 8.1 Hz, 1H), 2.50 (dd, *J* = 9.5, 5.2 Hz, 1H), 1.44 (d, *J* = 7.2 Hz, 9H) ppm.

^{13}C{H} NMR (101 MHz, $CDCl_3$) due to the amide structure and inhibited rotation some of the picks are doubled: δ 169.9, 169.8, 155.1, 139.3, 137.4, 137.3, 136.1, 132.9, 129.34, 129.25, 129.2, 129.1, 128.9, 128.73, 128.71, 128.61, 128.54, 128.51, 128.46, 128.4, 128.1, 127.8, 127.6, 127.3, 127.2, 126.92, 126.89, 126.7, 126.6, 126.5, 65.1, 50.6, 37.7, 34.5, 31.3, 29.7, 29.6, 28.4 ppm.

HRMS (EI): *m/z* calcd for $C_{30}H_{33}NO_4Na$: 494.2313 [*M+Na*]; found 494.2307.

IR (cm^{-1}): 3410, 3028, 2977, 1714, 1603, 1497, 1355, 1168.

1-2,3-diphenylcyclopropyl-2,2-dimethylpropan-1-one (**21**)

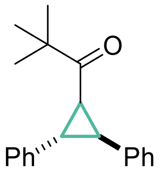

Following the general procedure, compound **21** was obtained from *trans*-stilbene (0.5 mmol) and 1-diazo-3,3-dimethylbutan-2-one (0.6 mmol). Reaction was performed for 18 h, with the I = 5 mA. The crude product was purified by column chromatography to using silica gel (hexane/AcOEt) afford 28 mg of compound **21** (white solid, yield = **20%**). Analytical data for compound **21** are in agreement with the literature data.[3]

^{1}H NMR (400 MHz, $CDCl_3$) δ 7.35 – 7.20 (m, 10H), 3.39 (dd, *J* = 7.2, 5.3 Hz, 1H), 3.03 (dd, *J* = 9.5, 7.1 Hz, 1H), 2.92 (dd, *J* = 9.5, 5.3 Hz, 1H), 1.09 (s, 9H) ppm.

^{13}C{H} NMR (101 MHz, $CDCl_3$) δ 208.9, 140.3, 135.7, 129.0, 128.6, 127.9, 126.8, 126.7, 126.6, 43.9, 37.4, 35.3, 29.7, 26.1 ppm.

2,3-diphenylcyclopropyl)(phenyl)methanone (**22**)

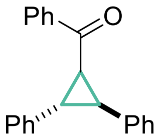

Following the general procedure, compound **22** was obtained from *trans*-stilbene (0.5 mmol) and 2-diazo-1-phenylethan-1-one (0.6 mmol). Reaction was performed for 18 h, with the I = 5 mA. The crude product was purified by column chromatography to using silica gel (hexane/AcOEt) afford 66 mg of compound **22** (white solid, yield = **45%**). Analytical data for compound **22** are in agreement with the literature data.[10]

^{1}H NMR (400 MHz, $CDCl_3$) δ 8.01 – 7.93 (m, 2H), 7.53 (d, *J* = 7.4 Hz, 1H), 7.47 – 7.41 (m, 2H), 7.41 – 7.12 (m, 10H), 3.64 (dd, *J* = 7.0, 5.3 Hz, 1H), 3.39 (dd, *J* = 9.5, 5.4 Hz, 1H), 3.30 (d, *J* = 7.0 Hz, 1H) ppm.

^{13}C{H} NMR (101 MHz, $CDCl_3$) δ 194.9, 140.0, 138.4, 135.6, 132.7, 129.1, 128.6, 128.5, 128.12, 128.08, 126.92, 126.89, 126.7, 37.9, 36.5, 30.0 ppm.

2,3-diphenylcyclopropyl)(4-methoxyphenyl)methanone (**23**)

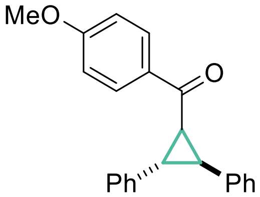

Following the general procedure, compound **23** was obtained from *trans*-stilbene (0.5 mmol) and 2-diazo-1-phenylethan-1-one (0.6 mmol). Reaction was performed for 18 h, with the I = 5 mA. The crude product was purified by column chromatography to using silica gel (hexane/AcOEt) afford 54 mg of compound **23** (white solid, yield = **33%**). Analytical data for compound **23** are in agreement with the literature data.[11]

^{1}H NMR (400 MHz, $CDCl_3$) δ 8.01 – 7.91 (m, 2H), 7.35 (m, 4H), 7.32 – 7.22 (m, 5H), 7.22 – 7.13 (m, 1H), 6.96 – 6.87 (m, 2H), 3.85 (s, 3H), 3.61 (dd, *J* = 6.9, 5.3 Hz, 1H), 3.34 (dd, *J* = 9.6, 5.4 Hz, 1H), 3.23 (dd, *J* = 9.6, 6.9 Hz, 1H) ppm.

^{13}C{H} NMR (101 MHz, $CDCl_3$) δ 193.3, 163.3, 140.3, 135.8, 131.5, 130.4, 129.0, 128.6, 128.1, 126.9, 126.8, 126.6, 113.7, 55.4, 37.4, 36.2, 29.6 ppm.

2,3-diphenylcyclopropyl)(4-nitrophenyl)methanone (**24**)

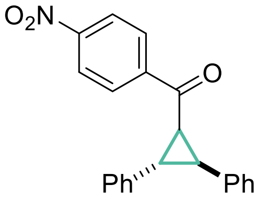

Following the general procedure, compound **24** was obtained from *trans*-stilbene (0.5 mmol) and 2-diazo-1-(4-methoxyphenyl)ethan-1-one (0.6 mmol). Reaction was performed for 18 h, with the I = 5 mA. The crude product was purified by column chromatography to using silica gel (hexane/AcOEt) afford 103 mg of compound **24** (yellow solid, yield = **60%**). Analytical data for compound **24** are in agreement with the literature data.[10]

^{1}H NMR (400 MHz, $CDCl_3$) δ 8.31 – 8.22 (m, 2H), 8.13 – 8.02 (m, 2H), 7.42 – 7.15 (m, 10H), 3.69 (dd, *J* = 7.0, 5.4 Hz, 1H), 3.48 – 3.26 (m, 2H) ppm.

^{13}C{H} NMR (101 MHz, $CDCl_3$) δ 193.6, 150.1, 142.7, 139.3, 134.9, 129.0, 128.8, 128.3, 127.3, 127.0, 126.9, 123.8, 38.6, 37.1, 30.5 ppm.

4. Electrochemical Radical Synthesis of Cyclobutanes from olefins and styrenes

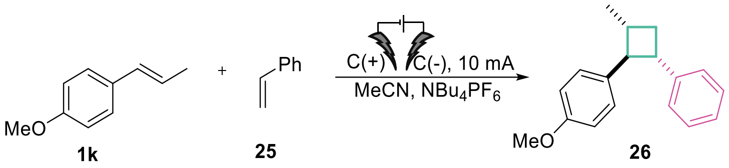

General procedure: A electrolyte (c = 0.125) was placed in a 5 mL vial with a septum and dissolved in MeCN (c = 0.15 M). Then, the reaction mixture was then degassed for 10 min with Ar and then trans-anethole (**1k**, 1.0 equiv.) and styrene (7.5 equiv.) were added and stirred under constant electrical power (I = 10 mA) for 5 hours. As both anode and cathode graphite electrodes were used with rapid altering polarity (1 min). The electrical power was was turned off, the reaction mixture was concentrated and subjected to flash column chromatography (hexane/AcOEt).

Optimization studies:

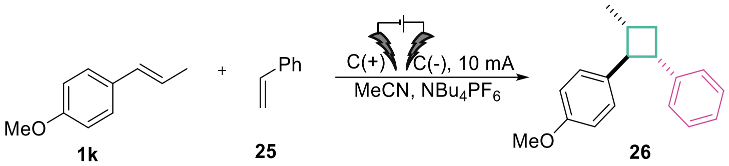

Table S7. Initial experiments

Entry	I [mA]	RAP	Time [h]	Yield [%]*
1	5	-	10	39
2	-	-	24	0
3[b]	-	-	24	0

Reaction conditions: *trans*-anethole (**1k**, 0.6 mmol, 1.0 equiv.), styrene (**25**, 5.0 equiv.), MeCN (c = 0.15 M), NBu_4PF_6 (c = 0.1 M), (+)$C_{graphite}$/(-)$C_{graphite}$, alternation of the polarity (ElectraSyn 2.0 IKA, 1 min), rt.[b] No electrolyte *Isolated yields

Table S8. Electrolyte

Entry	Electrolyte	Yield [%]*
1	$LiCF_3SO_2$	39
2	$LiBF_4$	38
**3**	**NBu_4PF_6**	**39**
4	NBu_4BF_4	35
**5**	NBu_4ClO_4	18
**6**	-	20

Reaction conditions: *trans*-anethole (**1k**, 0.6 mmol, 1.0 equiv.), styrene (**25**, 5.0 equiv.), MeCN (c = 0.15 M), electrolyte (c = 0.1 M), (+)$C_{graphite}$/(-)$C_{graphite}$, alternation of the polarity (ElectraSyn 2.0 IKA, 1 min), rt. *Isolated yields

Table S9. Optimization of electrical power vs time

Entry	Current [mA]	Time [h]	Yield [%]*
1	5 mA	10 h	39
**2**	**10 mA**	**5 h**	**60**

Reaction conditions: *trans*-anethole (**1k**, 0.6 mmol, 1.0 equiv.), styrene (**25**, 5.0 equiv.), MeCN (c = 0.15 M), NBu_4PF_6 (c = 0.1 M), (+)$C_{graphite}$/(-)$C_{graphite}$, alternation of the polarity (ElectraSyn 2.0 IKA, 1 min), rt. *Isolated yields

Table S10. Optimization of the substrates' ratio

Entry	alkene : styrene	Yield [%]*
1	1.0 : 5.0	60
**2**	**1.0 : 7.5**	**66**
3	1.0 : 10	66

Reaction conditions: *trans*-anethole (**1k**, 0.6 mmol, 1.0 equiv.), styrene (**25**, xx equiv.), MeCN (c = 0.15 M), NBu_4PF_6 (c = 0.1 M), (+)$C_{graphite}$/(-)$C_{graphite}$, alternation of the polarity (ElectraSyn 2.0 IKA, 1 min), rt. *Isolated yields

Table S11. Solvent concentration

Entry	Solvent Concentration [M]	Yield [%]*
1	0.2	48
**2**	**0.15**	**66**
3	0.12	50

Reaction conditions: *trans*-anethole (**1k**, 0.6 mmol, 1.0 equiv.), styrene (**25**, xx equiv.), MeCN (c = xx M), NBu_4PF_6 (c = 0.1 M), (+)$C_{graphite}$/(-)$C_{graphite}$, alternation of the polarity (ElectraSyn 2.0 IKA, 1 min), rt. *Isolated yields

Table S12. Electrolyte concentration

Entry	Electrolyte Concentration [M]	Yield [%]*
1	0.075	50
2	0.1	66
**3**	**0.125**	**70**

Reaction conditions: *trans*-anethole (**1k**, 0.6 mmol, 1.0 equiv.), styrene (**25**, xx equiv.), MeCN (c = 0.15 M), NBu_4PF_6 (c = xx M), (+)$C_{graphite}$/(-)$C_{graphite}$, alternation of the polarity (ElectraSyn 2.0 IKA, 1 min), rt. *Isolated yields

Scope and limitations studies:

1-methoxy-4-(2-methyl-4-phenylcyclobutyl)benzene (**26**)

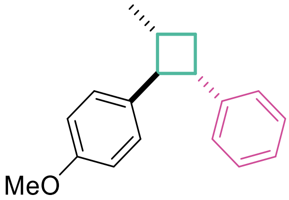

Following the general procedure, compound **26** was obtained from (*E*)-1-methoxy-4-(prop-1-en-1-yl)benzene (0.6 mmol) and styrene (7.5 equiv., 4.5 mmol). Reaction was performed for 5 h, with the I = 10 mA. The crude product was purified by column chromatography to using silica gel (hexane/AcOEt) afford 106 mg of compound **26** (colorless, yield = **70%**). Analytical data for compound **26** are in agreement with the literature data.[9]

^{1}H NMR (600 MHz, $CDCl_3$) δ 7.33 – 7.26 (m, 2H), 7.25 – 7.14 (m, 5H), 6.93 – 6.82 (m, 2H), 3.80 (s, 3H), 3.41 (td, *J* = 10.0, 7.9 Hz, 1H), 2.96 (t, *J* = 9.5 Hz, 1H), 2.57 – 2.50 (m, 1H), 2.42 – 2.23 (m, 1H), 1.72 (q, *J* = 10.1 Hz, 1H), 1.20 (d, *J* = 6.5 Hz, 3H) ppm.

^{13}C{H} NMR (126 MHz, $CDCl_3$) δ 158.1, 144.7, 135.9, 128.3, 127.8, 126.6, 125.9, 113.8, 55.6, 55.3, 44.1, 35.5, 33.9, 20.5 ppm.

1-methoxy-4-(2-methyl-4-(p-tolyl)cyclobutyl)benzene (**27**)

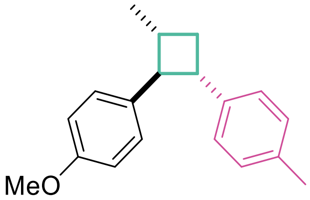

Following the general procedure, compound **27** was obtained from (*E*)-1-methoxy-4-(prop-1-en-1-yl)benzene (0.6 mmol) and 1-methyl-4-vinylbenzene (7.5 equiv., 4.5 mmol). Reaction was performed for 5 h, with the I = 10 mA. The crude product was purified by column chromatography to using silica gel (hexane/AcOEt) afford 109 mg of compound **27** (colorless oil, yield = **68%**). Analytical data for compound **27** are in agreement with the literature data.[11]

^{1}H NMR (500 MHz, $CDCl_3$) δ 7.20 – 7.13 (m, 2H), 7.08 (s, 4H), 6.87 – 6.79 (m, 2H), 3.78 (s, 3H), 3.34 (td, *J* = 10.0, 7.9 Hz, 1H), 2.92 (t, *J* = 9.5 Hz, 1H), 2.55 – 2.44 (m, 1H), 2.31 (s, 3H), 1.67 (q, *J* = 10.1 Hz, 1H), 1.18 (d, *J* = 6.5 Hz, 3H) ppm.

^{13}C{H} NMR (126 MHz, $CDCl_3$) δ 158.0, 141.7, 135.9, 135.4, 128.9, 127.8, 126.5, 113.7, 55.6, 55.3, 43.8, 35.4, 34.1, 21.0, 20.5 ppm.

1-chloro-4-(2-(4-methoxyphenyl)-3-methylcyclobutyl)benzene (**28**)

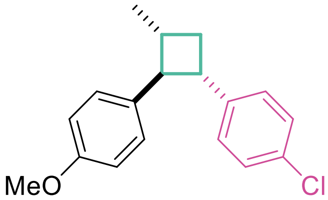

Following the general procedure, compound **28** was obtained from (*E*)-1-methoxy-4-(prop-1-en-1-yl)benzene (0.6 mmol) and 1-chloro-4-vinylbenzene (7.5 equiv., 4.5 mmol). Reaction was performed for 5 h, with the I = 10 mA. The crude product was purified by column chromatography to using silica gel (hexane/AcOEt) afford 106 mg of compound **28** (colorless oil, yield = **62%**). Analytical data for compound **28** are in agreement with the literature data.[11]

^{1}H NMR (500 MHz, $CDCl_3$) δ δ 7.24 – 7.19 (m, 2H), 7.16 – 7.13 (m, 2H), 7.12 – 7.07 (m, 2H), 6.91 – 6.76 (m, 2H), 3.79 (s, 3H), 3.33 (td, *J* = 10.0, 7.9 Hz, 1H), 2.87 (t, *J* = 9.5 Hz, 1H), 2.50 (dt, *J* = 10.3, 7.7 Hz, 1H), 2.41 – 2.23 (m, 1H), 1.65 (q, *J* = 10.1 Hz, 1H), 1.17 (d, *J* = 6.5 Hz, 3H) ppm.

^{13}C{H} NMR (126 MHz, $CDCl_3$) δ 158.1, 143.1, 135.4, 131.6, 128.3, 127.9, 127.7, 113.8, 55.8, 55.3, 43.7, 35.4, 33.9, 20.4 ppm.

1-bromo-4-(2-(4-methoxyphenyl)-3-methylcyclobutyl)benzene (**29**)

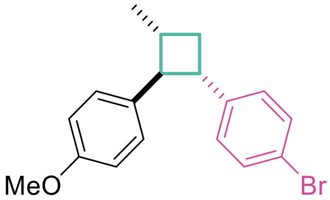

Following the general procedure, compound **28** was obtained from (*E*)-1-methoxy-4-(prop-1-en-1-yl)benzene (0.6 mmol) and 1-bromo-4-vinylbenzene (7.5 equiv., 6 mmol). Reaction was performed for 5 h, with the I = 10 mA. The crude product was purified by column chromatography to using silica gel (hexane/AcOEt) afford 79 mg of compound **28** (colorless oil, yield = **40%**). Analytical data for compound **28** are in agreement with the literature data.[11]

^{1}H NMR (500 MHz, $CDCl_3$) δ 7.41 – 7.34 (m, 2H), 7.18 – 7.11 (m, 2H), 7.09 – 7.00 (m, 2H), 6.90 – 6.80 (m, 2H), 3.80 (s, 3H), 3.32 (td, *J* = 10.0, 7.9 Hz, 1H), 2.88 (t, *J* = 9.5 Hz, 1H), 2.55 – 2.46 (m, 1H), 2.41 – 2.28 (m, 1H), 1.65 (q, *J* = 10.1 Hz, 1H), 1.18 (d, *J* = 6.5 Hz, 3H) ppm.

^{13}C{H} NMR (126 MHz, $CDCl_3$) δ 158.2, 143.6, 135.4, 131.3, 128.3, 127.7, 119.6, 113.8, 55.7, 55.3, 43.7, 35.4, 33.8, 20.40 ppm.

1-fluoro-4-(2-(4-methoxyphenyl)-3-methylcyclobutyl)benzene (**30**)

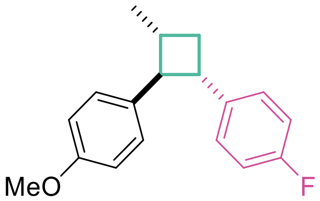

Following the general procedure, compound **29** was obtained from (*E*)-1-methoxy-4-(prop-1-en-1-yl)benzene (0.6 mmol) and 1-fluoro-4-vinylbenzene (7.5 equiv., 4.5 mmol). Reaction was performed for 5 h, with the I = 10 mA. The crude product was purified by column chromatography to using silica gel (hexane/AcOEt) afford 120 mg of compound **29** (colorless oil, yield = **74%**). Analytical data for compound **29** are in agreement with the literature data.[11]

^{1}H NMR (500 MHz, $CDCl_3$ δ 7.22 – 7.12 (m, 4H), 7.03 – 6.93 (m, 2H), 6.92 – 6.85 (m, 2H), 3.83 (s, 3H), 3.45 – 3.27 (m, 1H), 2.91 (t, *J* = 9.5 Hz, 1H), 2.63 – 2.48 (m, 1H), 2.41 – 2.29 (m, 1H), 1.69 (q, *J* = 10.1 Hz, 1H), 1.22 (d, *J* = 6.5 Hz, 3H) ppm.

^{13}C{H} NMR (126 MHz, $CDCl_3$) δ 162.3, 160.3, 158.2, 140.3, 140.3, 135.5, 127.9, 127.9, 127.7, 115.0, 114.8, 113.8, 76.8, 55.9, 55.3, 43.5, 35.4, 34.1, 20.41 ppm.

5. Mechanistic consideration

Mechanistic proposal for the electrochemical [2+1] (path a) and [2+2] (path b) cycloadditions of alkene radical cations:

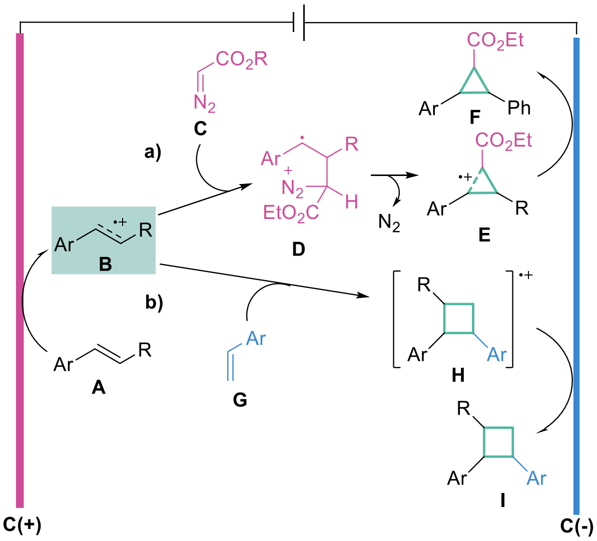

5a. Addition of radical scavengers:

I) TEMPO quenching experiment

[2+1] cycloaddition:

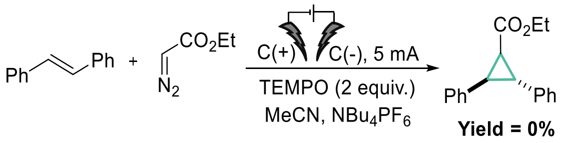

General procedure: A electrolyte (0.5 mmol in 4 mL MeCN, c = 0.125), olefin and TEMPO (2 equiv.) were placed in a 5 mL vial with a septum and dissolved in dry MeCN (c = 0.125 M). The reaction mixture was then degassed for 10 min with Ar and then diazo carbonyl compound (1.2 equiv.) was added and stirred under constant electrical power (I = 5 mA) for 10h. As both anode and cathode graphite electrodes were used with rapid altering polarity (1 min). The electrical power was turned off, the reaction mixture was concentrated and checked for product.

Results: The addition of TEMPO (at the beginning of the reaction) stops the reaction completely, thus confirming the radical nature of the transformation.

[2+2] cycloaddition:

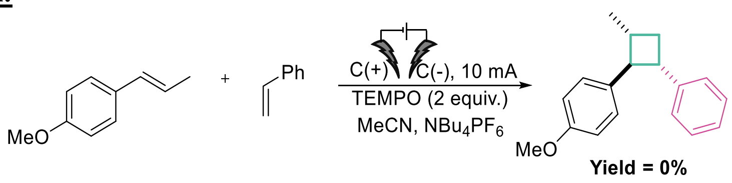

General procedure: A electrolyte (c = 0.125) and TEMPO (2 equiv.) was placed in a 5 mL vial with a septum and dissolved in MeCN (c = 0.15 M). Then, the reaction mixture was then degassed for 10 min with Ar and then *trans*-anethole (1.0 equiv.) and styrene (7.5 equiv.) were added and stirred under constant electrical power (I = 10 mA) for 5 hours. As both anode and cathode graphite electrodes were used with rapid altering polarity (1 min). The electrical power was was turned off, the reaction mixture was concentrated and and checked for product.

Results: The addition of TEMPO (at the beginning of the reaction) stops the reaction completely, thus confirming the radical nature of the transformation.

5b. *Cis* vs *trans* selectivity

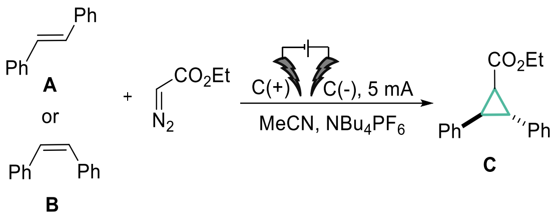

General procedure: A electrolyte (0.5 mmol in 4 mL MeCN, c = 0.125), olefin (A or B) were placed in a 5 mL vial with a septum and dissolved in dry MeCN (c = 0.125 M). The reaction mixture was then degassed for 10 min with Ar and then ethyl diazoacetate (1.2 equiv.) was added and stirred under constant electrical power (I = 5 mA) for 10h. As both anode and cathode graphite electrodes were used with rapid altering polarity (1 min). The electrical power was turned off, the reaction mixture was concentrated and checked for product.

GC chromatograms after reactions for A (black) and B (green):

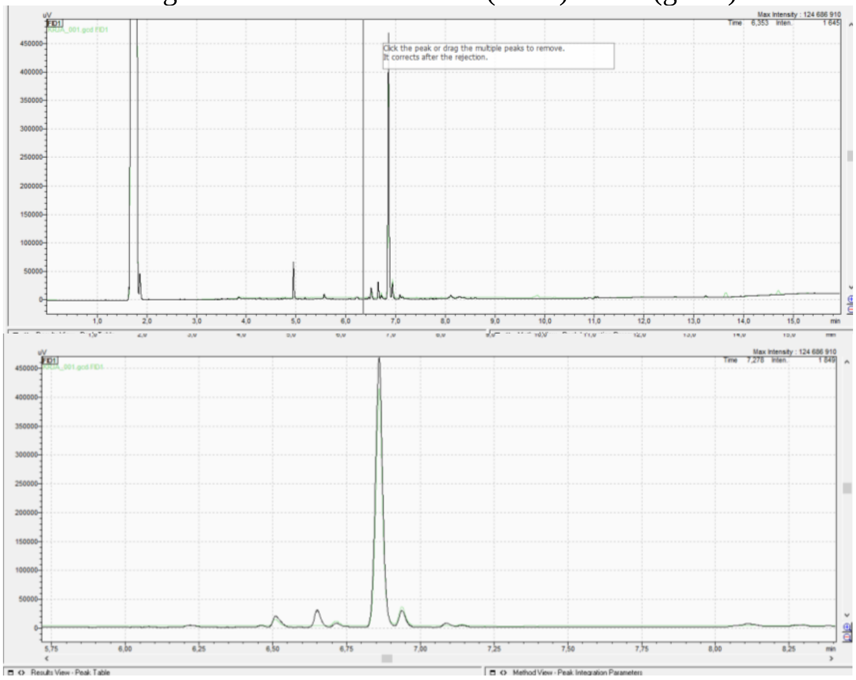

NMR data for the both products is also the same.

Results: Both olefins with an *E* or *Z* configuration gave only *trans*-cyclopropane, suggesting that the reaction proceeds via a radical-cation intermediate. The only difference between reaction for A and B is the yield of the reaction (A – 82%, and B – 67%), suggesting that *trans*-olefin undergoes cyclopropanation faster and isomer cis.

5c. Undivided vs divided electrochemical cell

[2+1] cycloaddition:

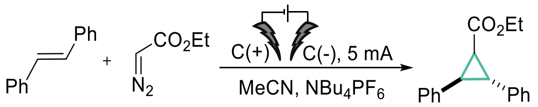

Undivided cell: A electrolyte (0.5 mmol in 4 mL MeCN, c = 0.125), olefin (A or B) were placed in a 5 mL vial with a septum and dissolved in dry MeCN (c = 0.125 M). The reaction mixture was then degassed for 10 min with Ar and then ethyl diazoacetate (1.2 equiv.) was added and stirred under constant electrical power (I = 5 mA) for specific amount of time. As both anode and cathode graphite

electrodes were used with rapid altering polarity (1 min). The electrical power was turned off, the reaction mixture was concentrated and checked for product.

Results:

2e$^-$ process for 0.6 mmol = 1.2 F/ mol (theoretical)
Yield after 1.2 F/mol = 67% (not full conversion of substrate)
Yield after 1.8 F/mol = 79% (full conversion of substrate)

Divided cell (H-Cell): *Anodic chamber*: An electrolyte (0.5 mmol in 4 mL MeCN, c = 0.125), olefin were placed in a 5 mL vial with a septum and dissolved in dry MeCN (c = 0.125 M). The reaction mixture was then degassed for 10 min with Ar and then ethyl diazoacetate (1.2 equiv.) was added. Cathodic chamber: an electrolyte (0.5 mmol) in 4 mL MeCN (c = 0.125) and DCM (100 µl) for the sacrificial reaction on cathode. Next reaction mixture was stirred under constant electrical power (I = 5 mA) for specific amount of time. Next, the electrical power was turned off, the reaction mixture was concentrated and checked for product.

Results:

2e$^-$ process for 0.6 mmol = 1.2 F/ mol (theoretical)
Yield after 1.2 F/mol < 10% (full conversion of substrate)

[2+2] cycloaddition:

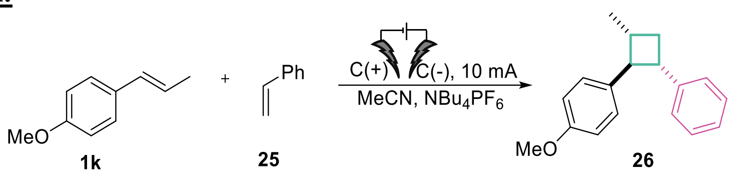

Undivided cell: A electrolyte (c = 0.125) was placed in a 5 mL vial with a septum and dissolved in MeCN (c = 0.15 M). Then, the reaction mixture was then degassed for 10 min with Ar and then *trans*-anethole (**1k**, 1.0 equiv.) and styrene (7.5 equiv.) were added and stirred under constant electrical power (I = 10 mA) for 5 hours. As both anode and cathode graphite electrodes were used with rapid altering polarity (1 min). The electrical power was was turned off, the reaction mixture was concentrated and checked for product.

Results:

2e$^-$ process for 0.6 mmol = 1.2 F/ mol (theoretical)
Yield after 1.2 F/mol = 67% (not full conversion of substrate)
Yield after 1.6 F/mol = 78% (full conversion of substrate)

Divided cell (H-Cell): *Anodic chamber*: An electrolyte (0.5 mmol in 4 mL MeCN, c = 0.125), *trans*-anethole (**1k**, 05. Mmol, 1.0 equiv.) were placed in a 5 mL vial with a septum and dissolved in dry MeCN (c = 0.125 M). The reaction mixture was then degassed for 10 min with Ar and then styrene (7.5 equiv.) was added. Cathodic chamber: an electrolyte (0.5 mmol) in 4 mL MeCN (c = 0.125) and DCM (100 µl) for the sacrificial reaction on cathode. Next reaction mixture was stirred under constant electrical power (I = 10 mA) for specific amount of time. Next, the electrical power was turned off, the reaction mixture was concentrated and checked for product.

Results:

2e$^-$ process for 0.6 mmol = 1.2 F/ mol (theoretical)
Yield after 1.2 F/mol = 19% (full conversion of substrate)

Conclusions:

[2+1] cycloaddition:

Based on the aforementioned results and the literature data, we propose a plausible radical reaction pathway for the electrochemical [2+1] cycloaddition of alkene cation radicals (see Scheme from S19 for the mechanistic proposal). The first step is anodic oxidation of the olefin **A** that generates an olefin electrophilic radical cation **B**. In the case of [2+1] cycloaddition, radical cation **B**, which undergoes a reaction with nucleophilic diazo compound **C** to generate radical cation **D**, which after spontaneous nitrogen extrusion forms radical cation **E**. Subsequent reduction at the cathode generates cyclopropane

F. Of course, we cannot exclude the possibility of a radical chain reaction and subsequent reduction of radical cation **E** by olefin **A**, but since the reaction does not proceed effectively in H-cell (yield < 10%) it is unlikely.

[2+2] cycloaddition:

Based on the aforementioned results and the literature data, we propose a plausible radical reaction pathway for the electrochemical [2+2] cycloaddition of alkene cation radicals (see Scheme from S19 for the mechanistic proposal). The first step is anodic oxidation of the olefin **A** that generates an olefin electrophilic radical cation **B**. In the case of [2+2] cycloaddition, radical cation **B**, which undergoes a reaction with styrene derivative **G** to generate radical cation **H**, which after subsequent reduction at the cathode generates cyclopropane **I**. Of course, we cannot exclude the possibility of a radical chain reaction and subsequent reduction of radical cation **H** by olefin **A**, especially that in the case of [2+2] cycloaddition the reaction proceeds more effectively in H-cell (yield = 19%) then in the case of [2+1].

5d. Proton-migrated by product

As it was previously reported by Ferreira and Kang, [2+1] cycloaddition between olefin radical cations and diazo compounds gives beside the cyclopropane product (80%), proton-migrated by product (less then 6%).

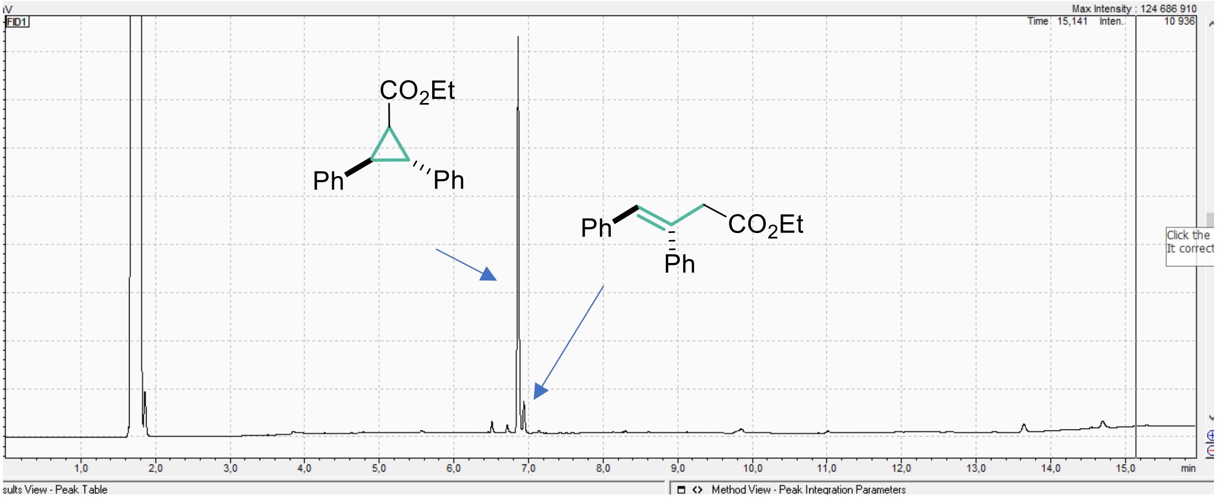

6. NMR spectra

Ethyl 2,3-diphenylcyclopropane-1-carboxylate (3)

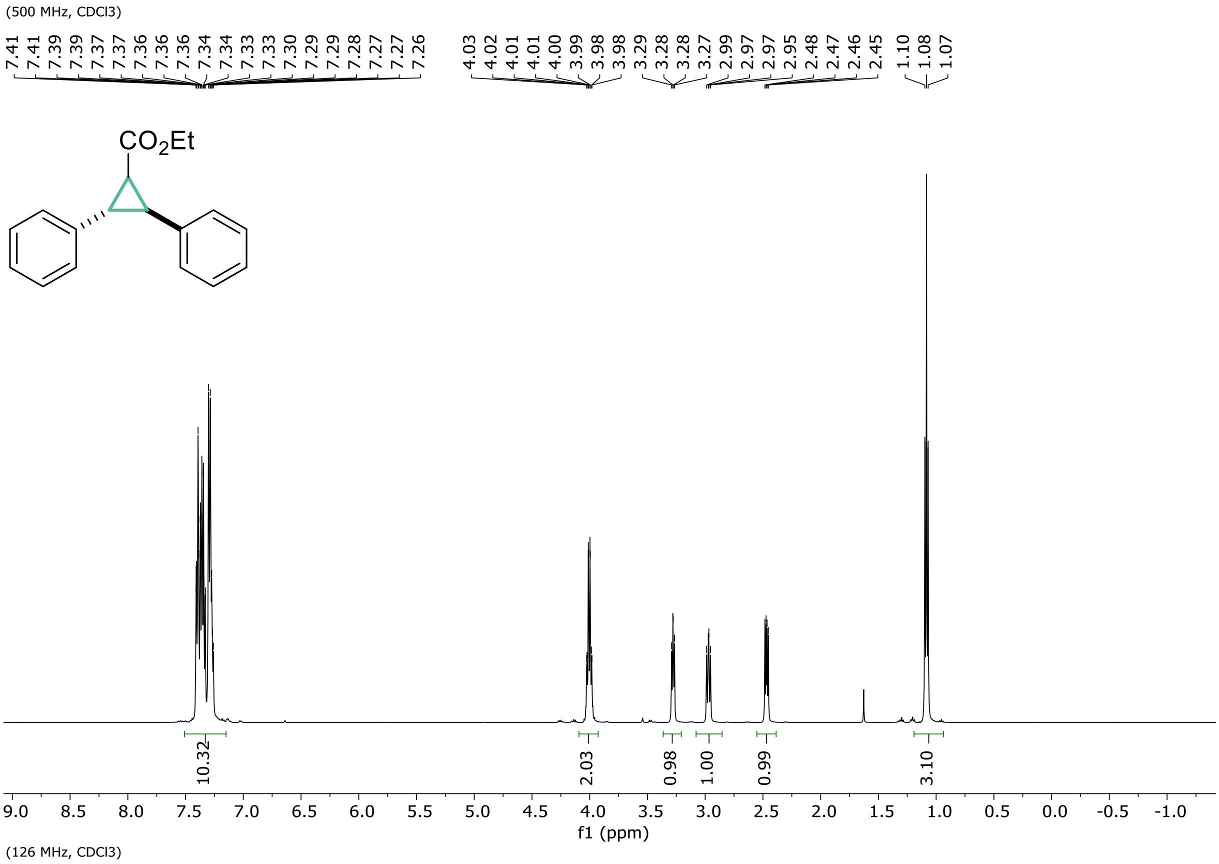

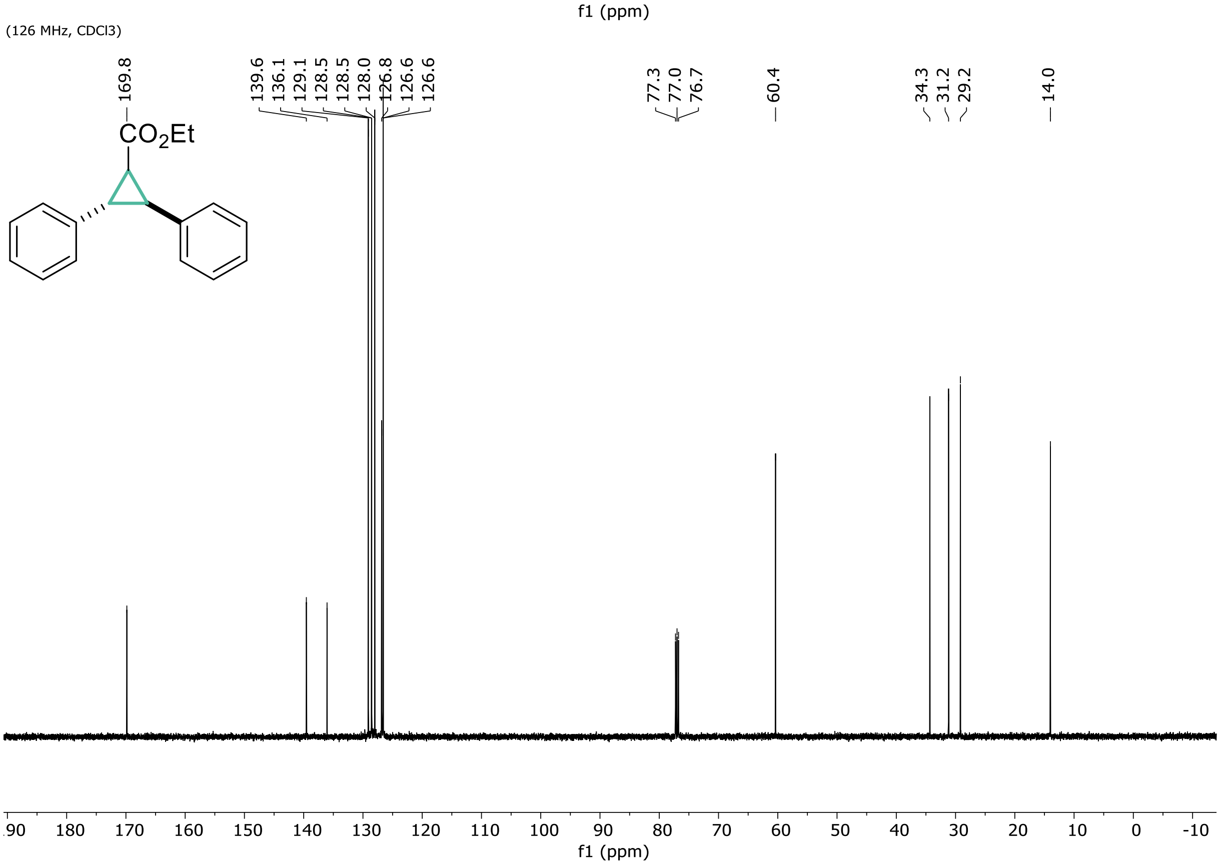

Ethyl 2,3-di-*p*-tolylcyclopropane-1-carboxylate (4)

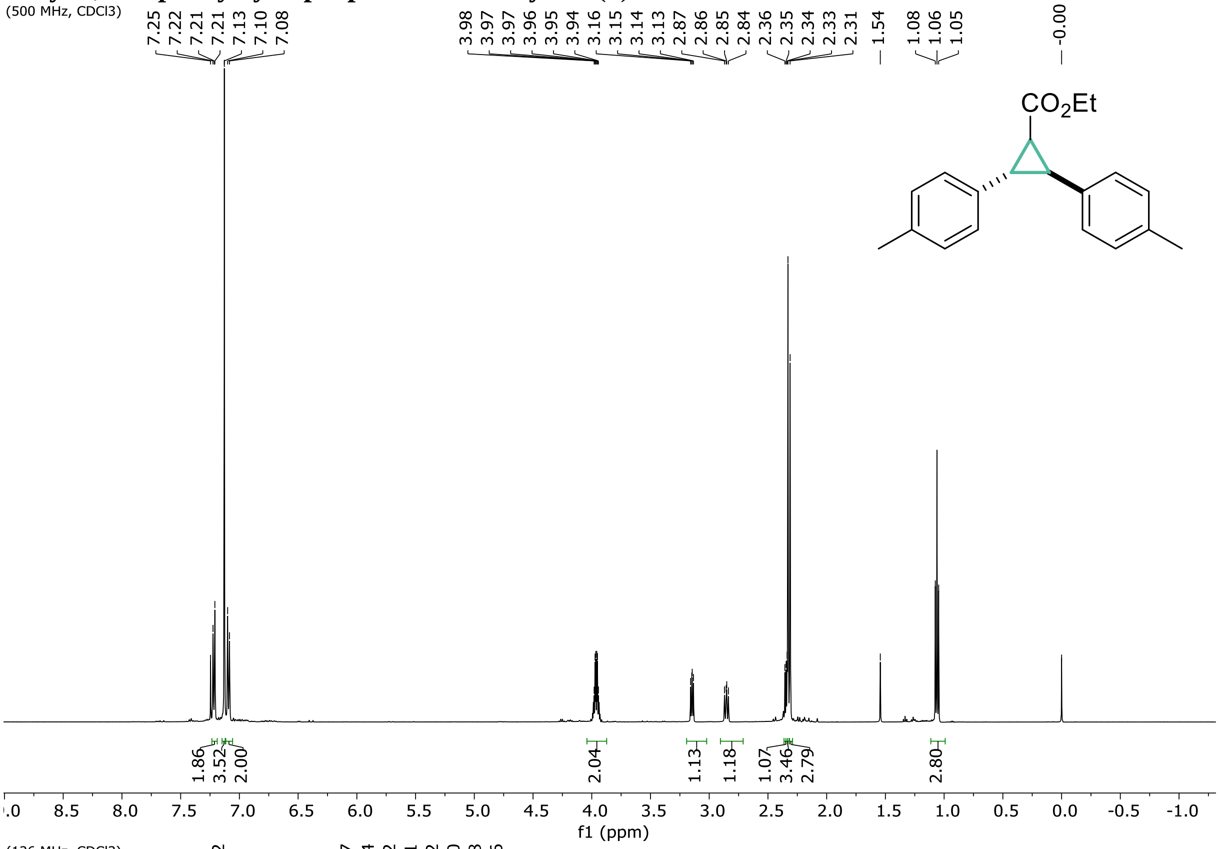

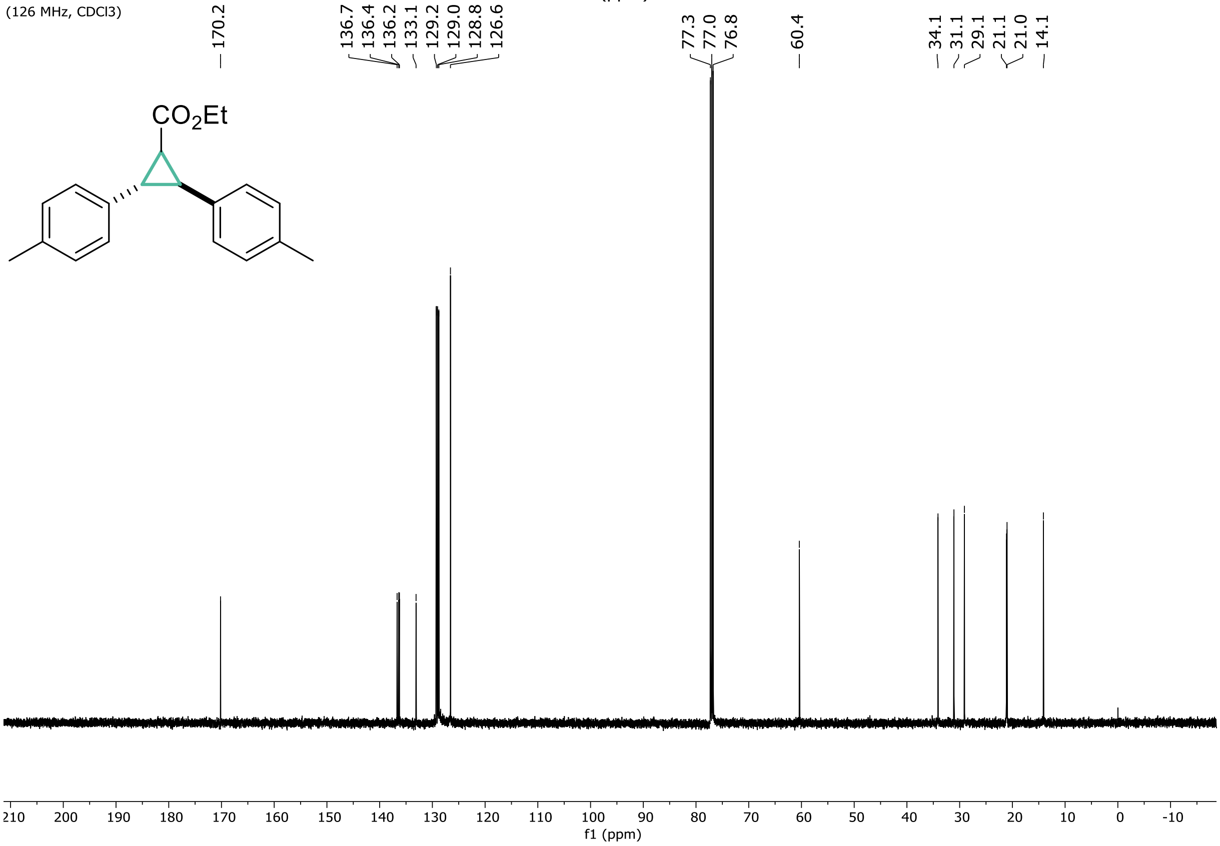

Ethyl 2,3-bis(4-bromophenyl)cyclopropane-1-carboxylate (5)

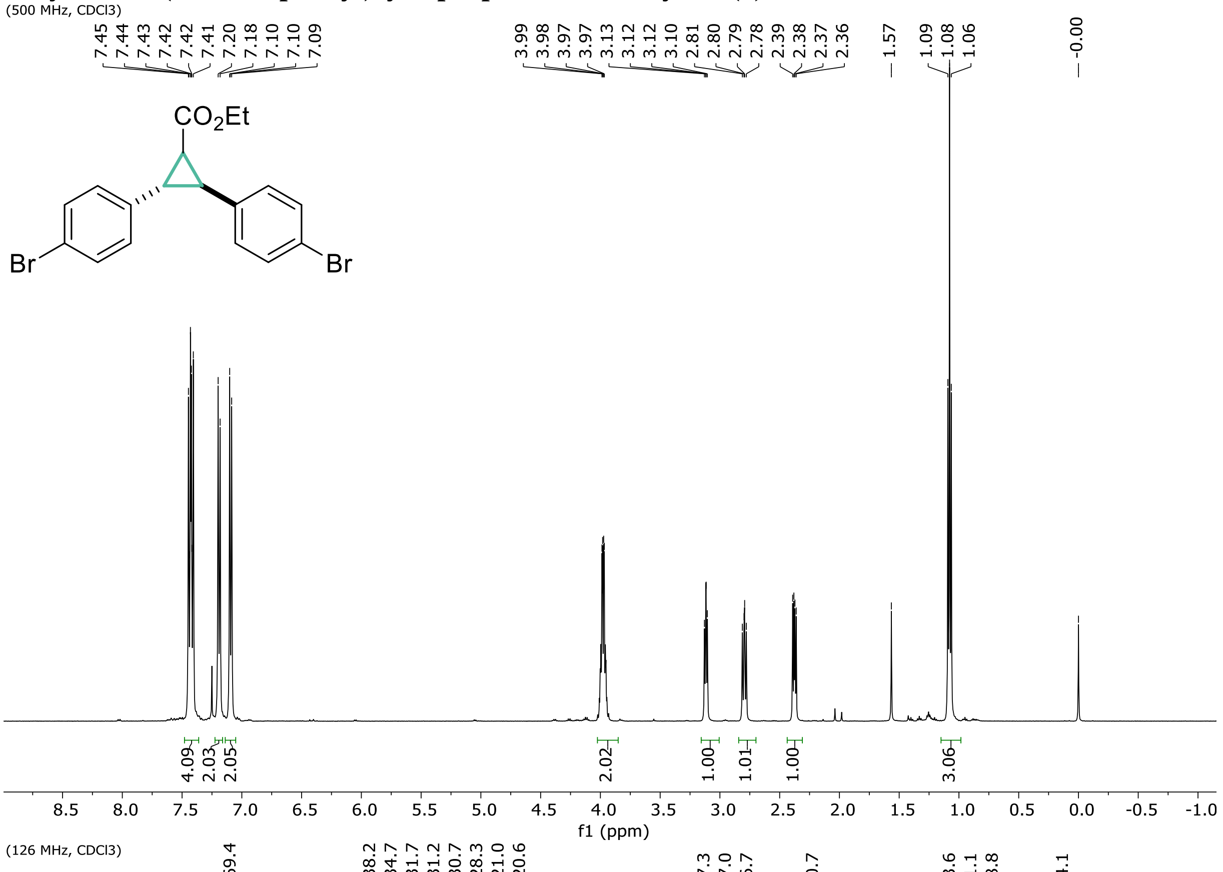

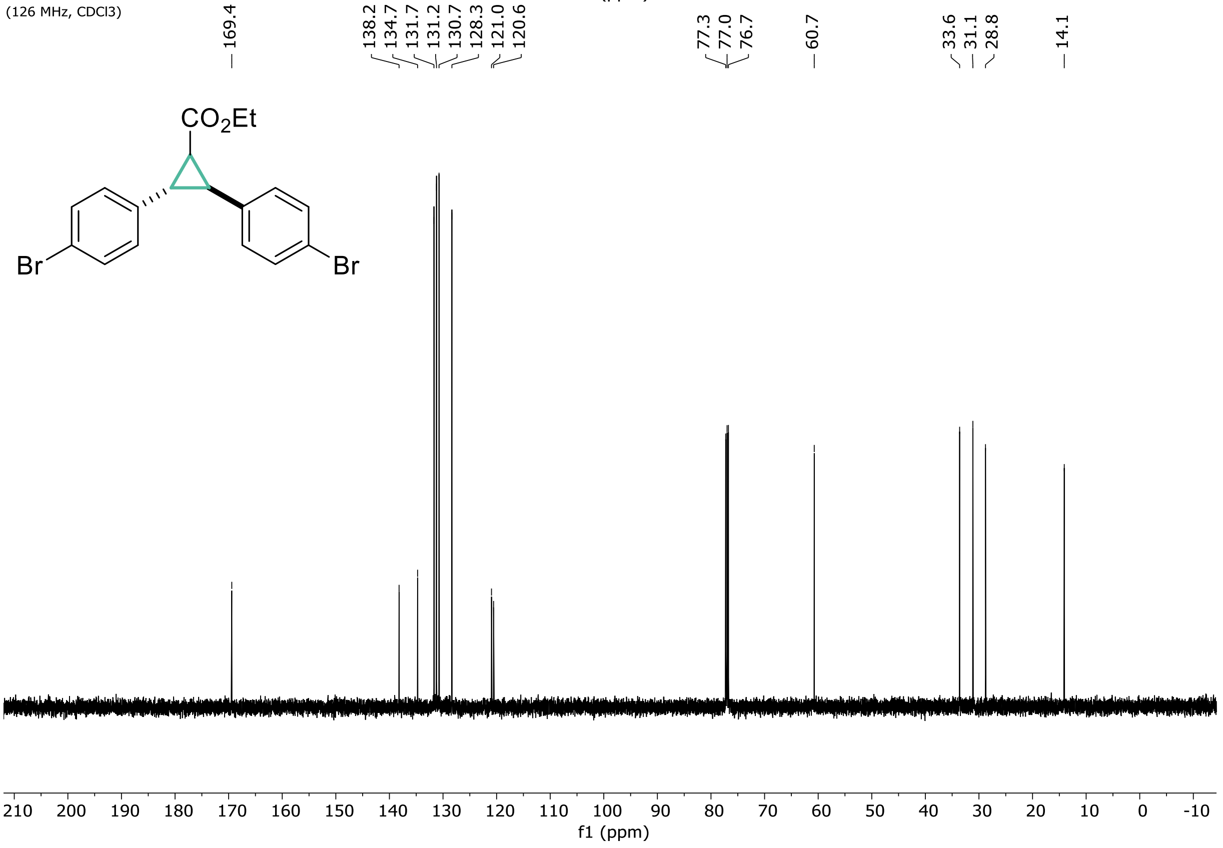

Ethyl 2,3-bis(4-fluorophenyl)cyclopropane-1-carboxylate (6)

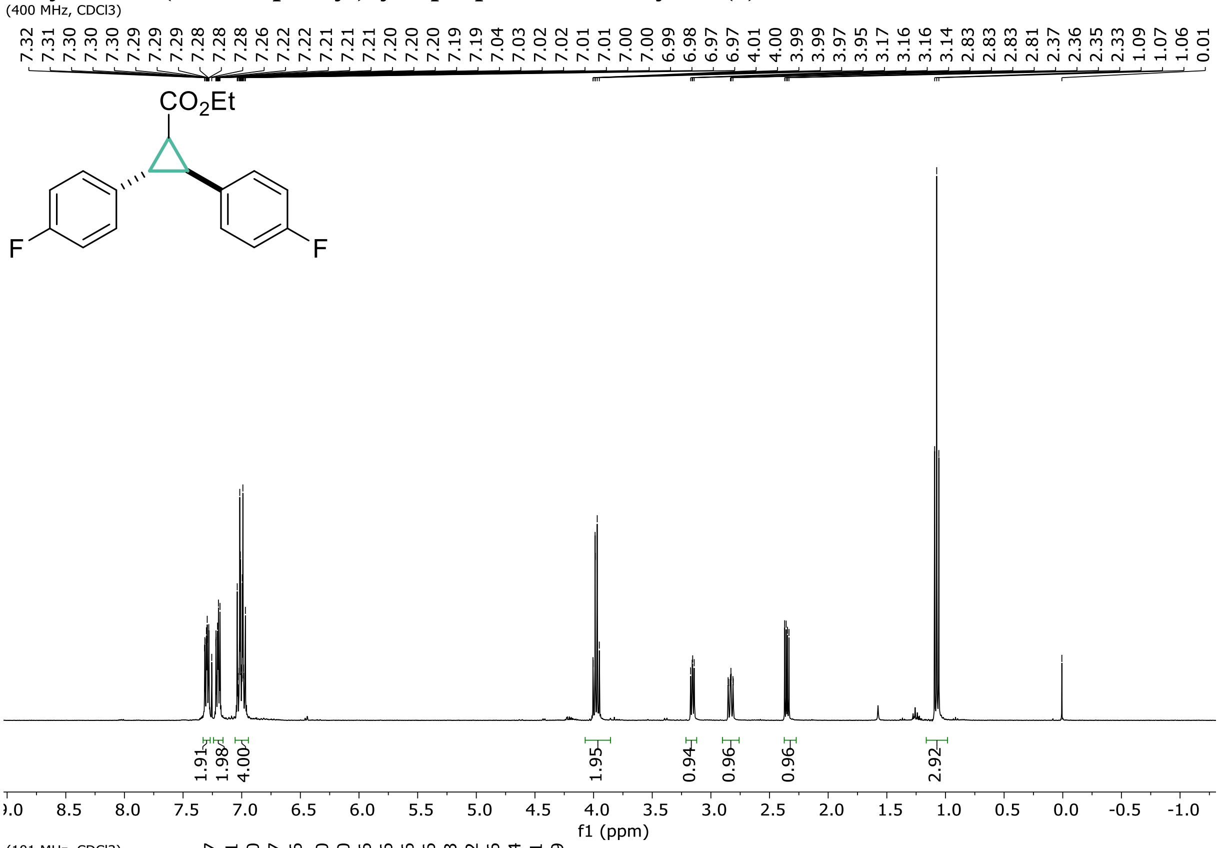

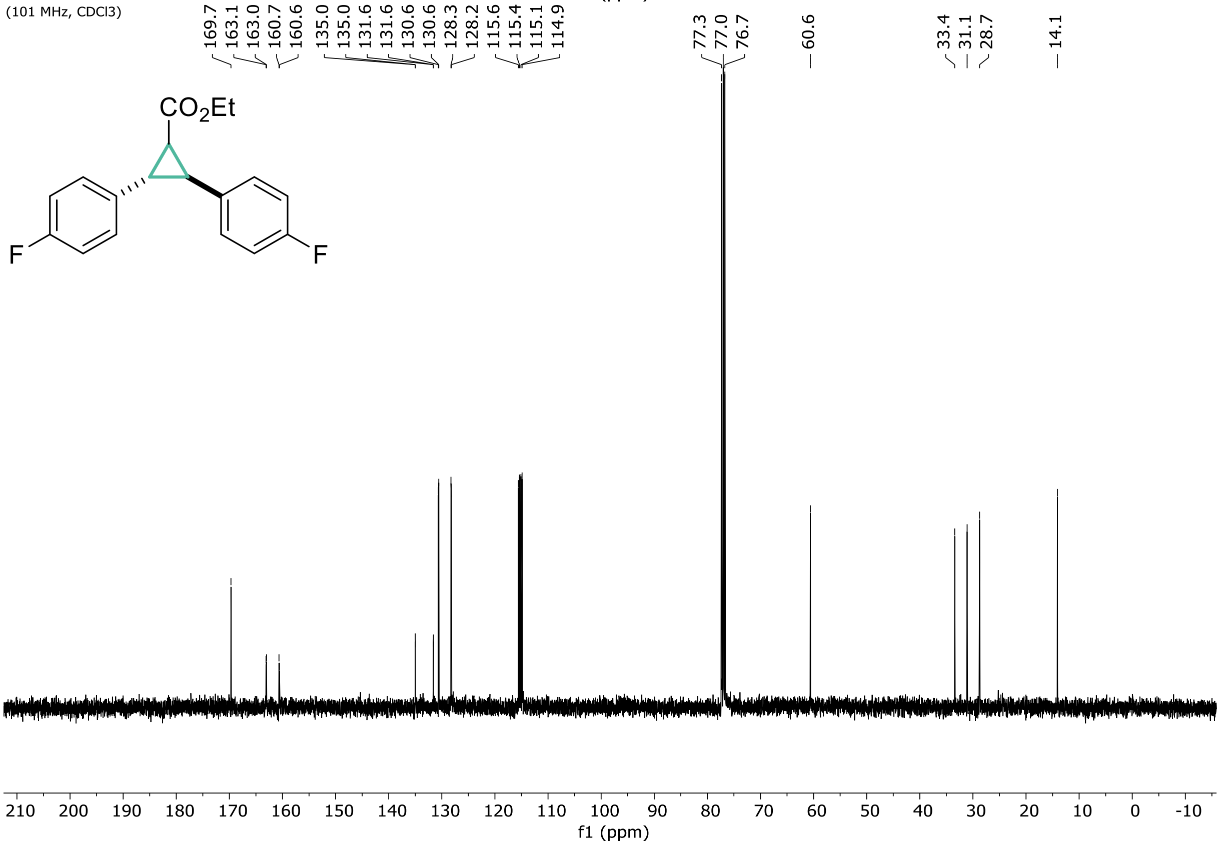

Ethyl 2-(4-nitrophenyl)-3-phenylcyclopropane-1-carboxylate (1,2-*anti*-8)

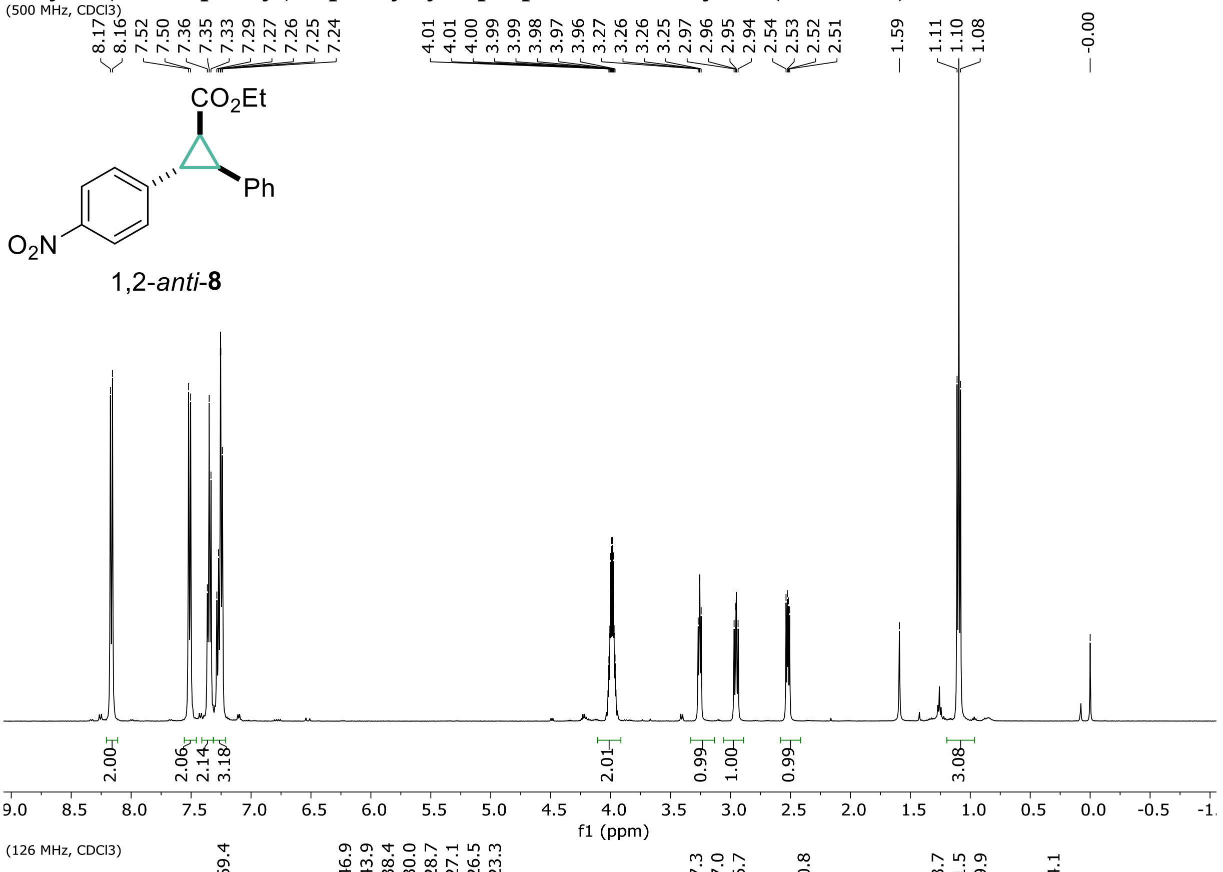

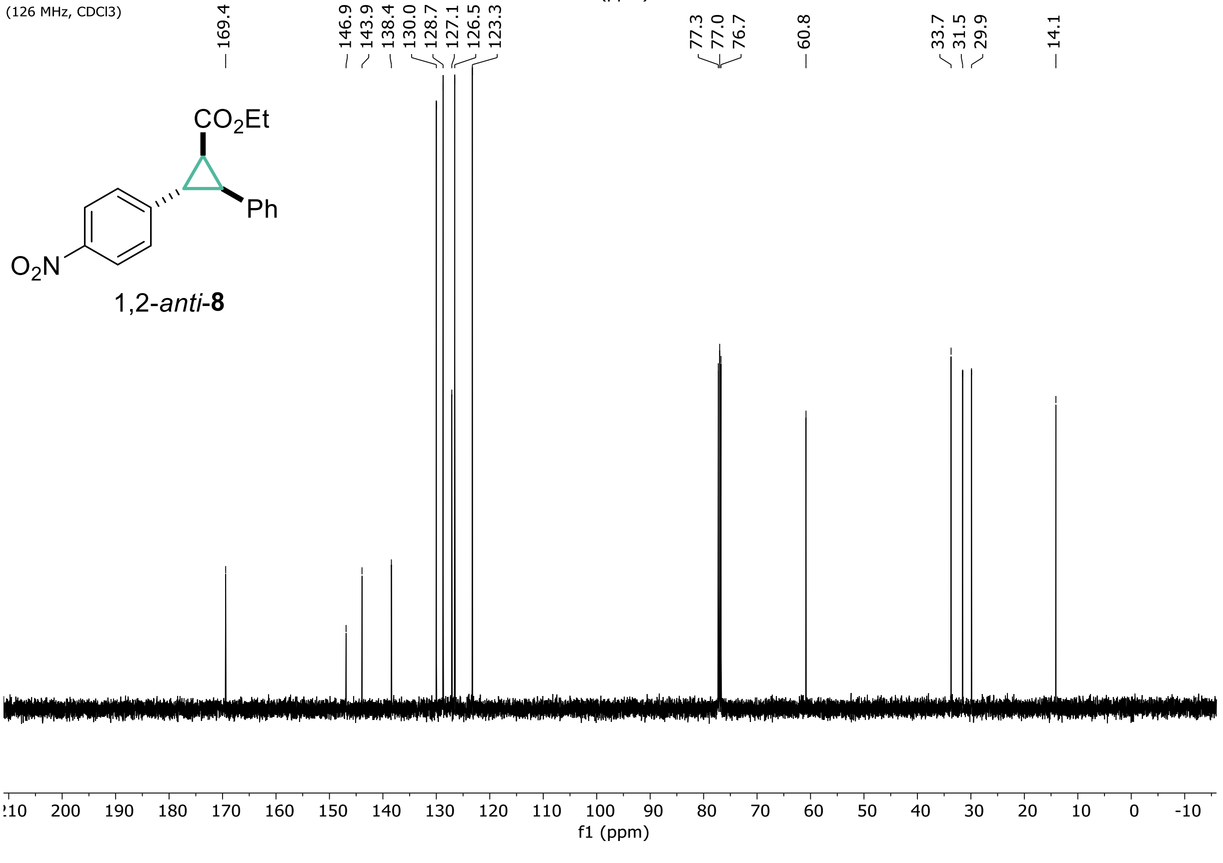

Ethyl 2-(4-nitrophenyl)-3-phenylcyclopropane-1-carboxylate (1,2-*syn*-8)

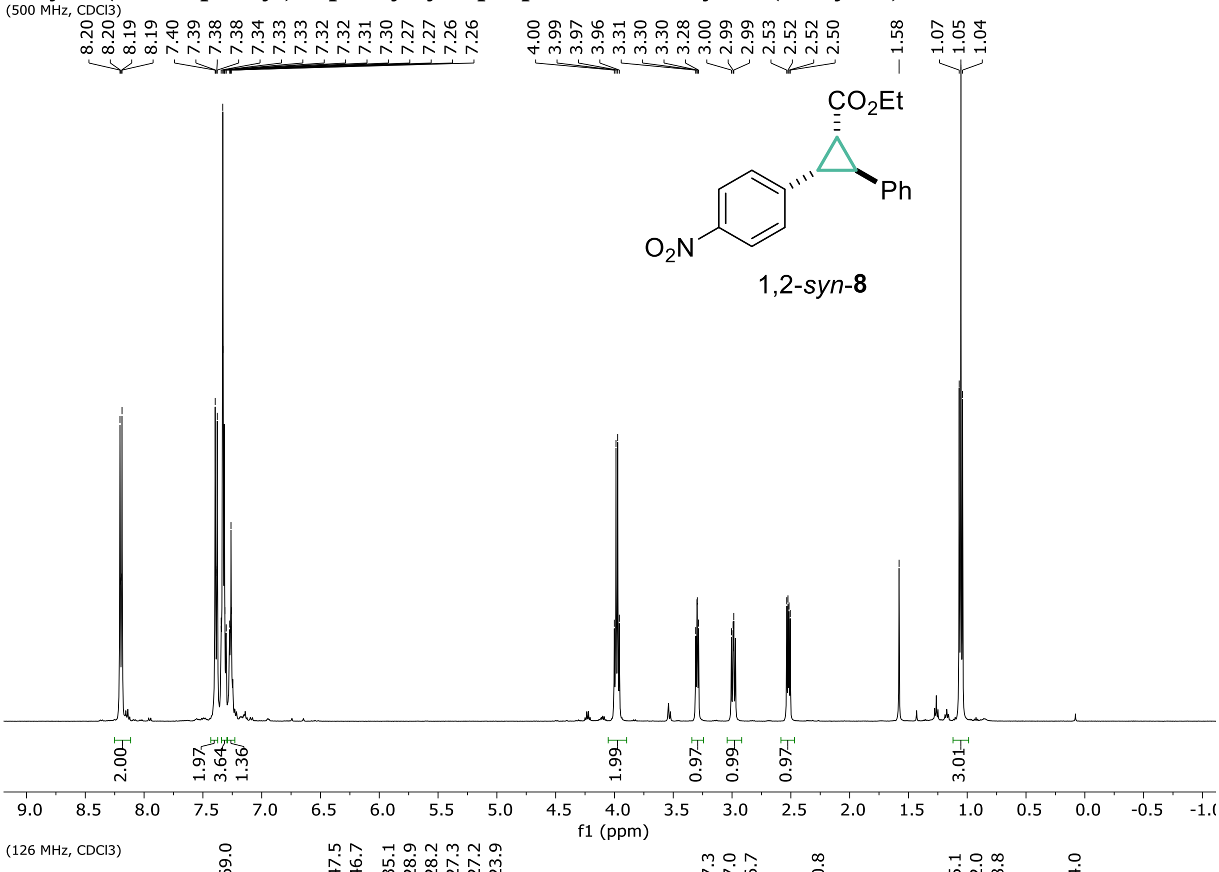

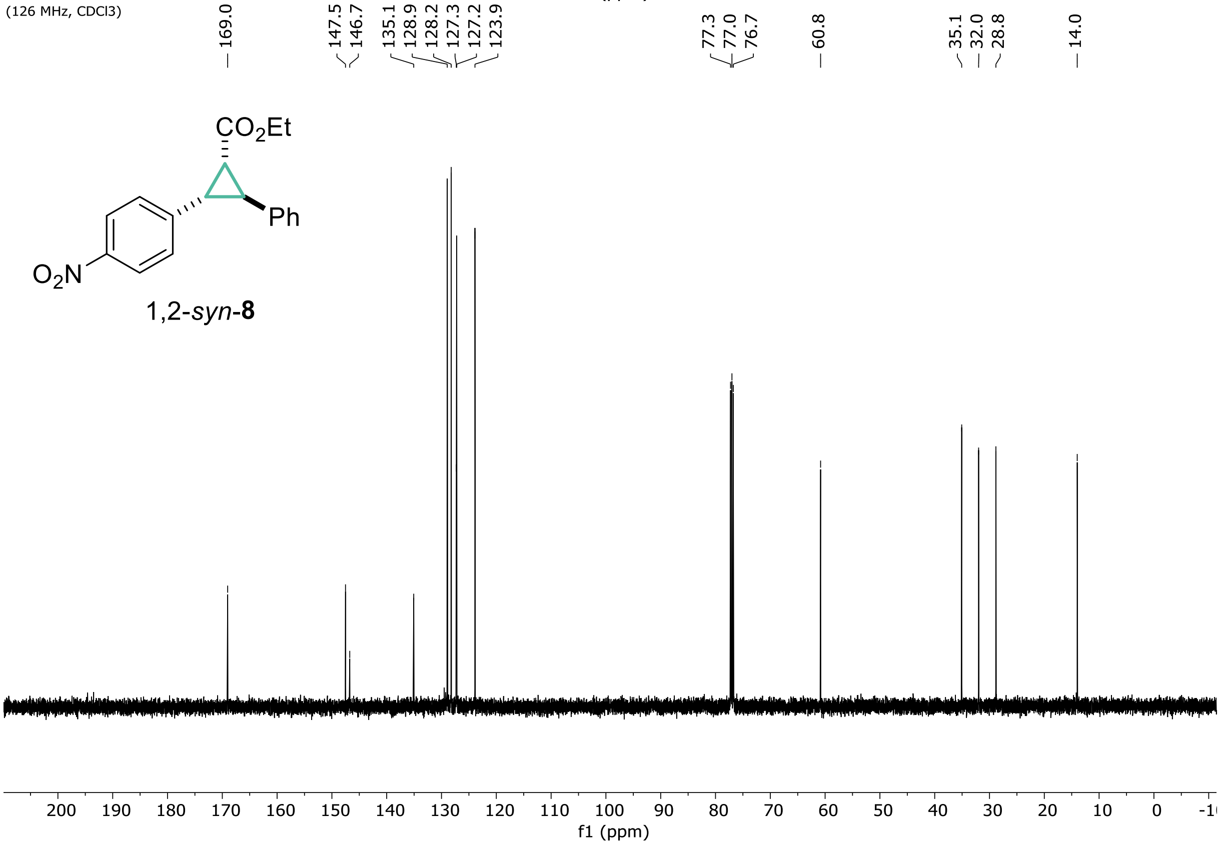

Ethyl 4-(2-(ethoxycarbonyl)-3-phenylcyclopropyl)benzoate (9) (mixture of *syn* and *anti* isomers 1:1)

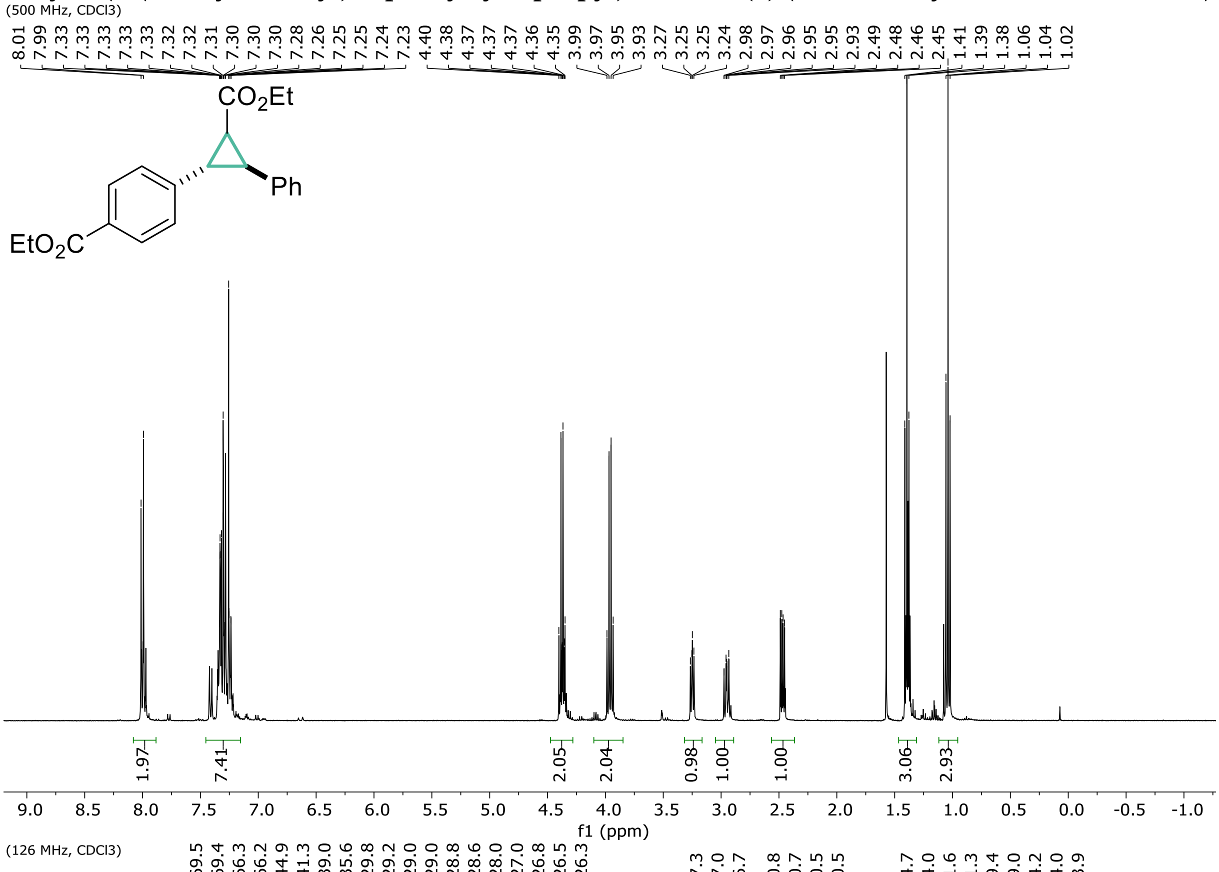

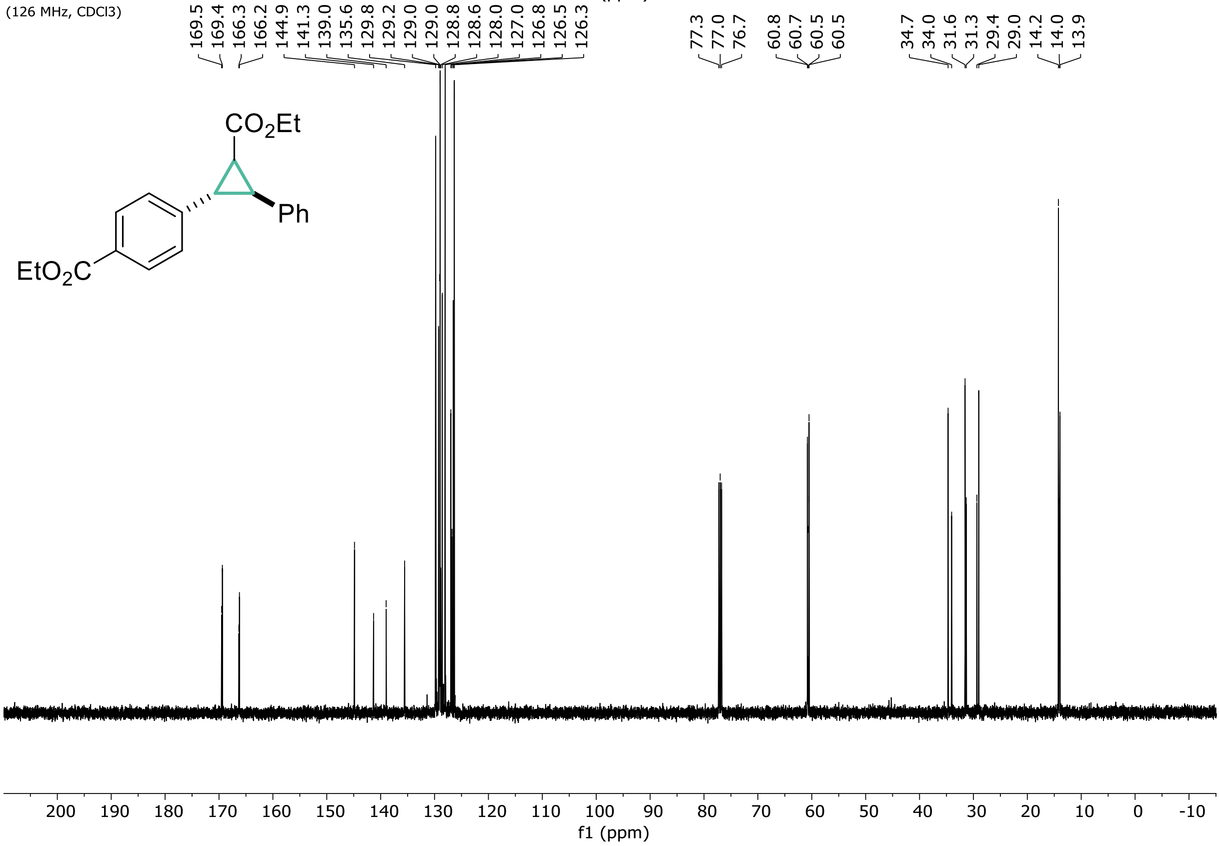

Ethyl 2-(4-bromophenyl)-3-phenylcyclopropane-1-carboxylate (**10**) (mixture of *syn* and *anti* isomers 1:1)

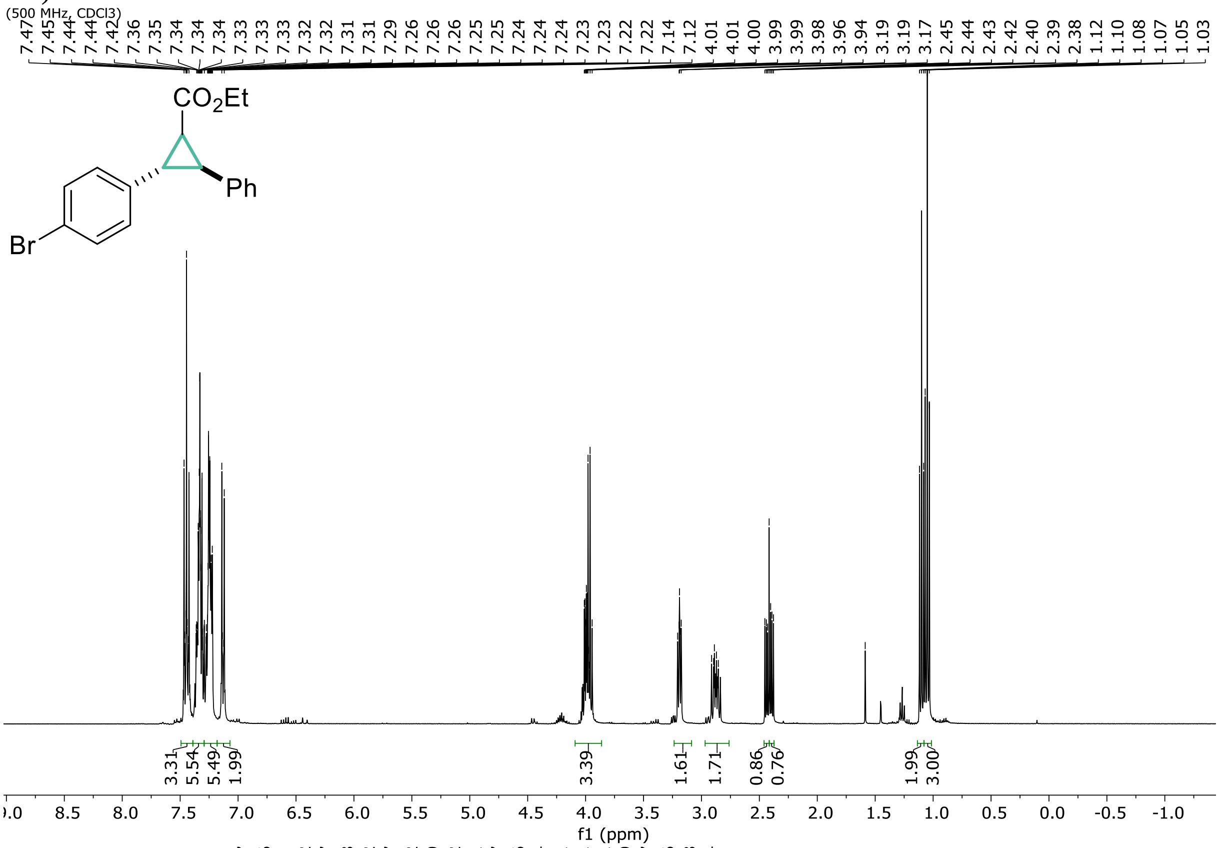

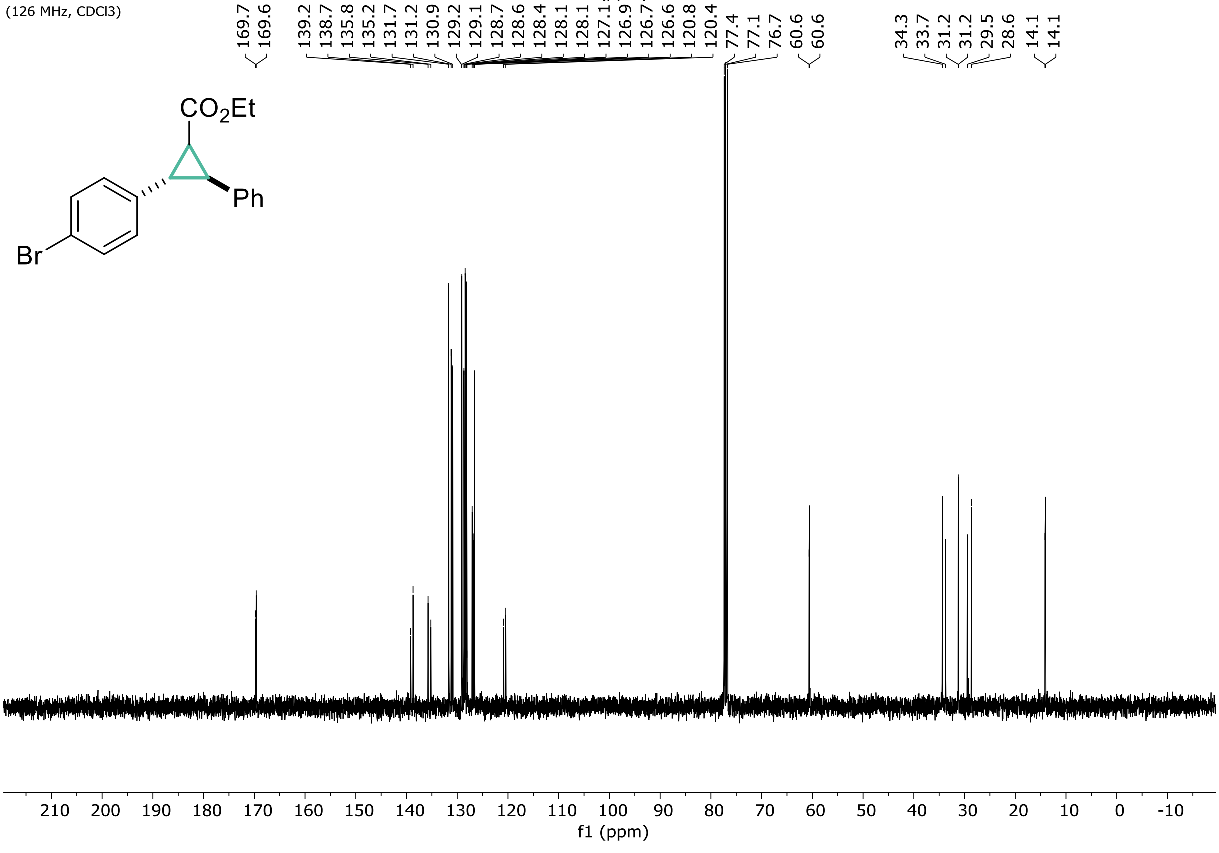

Ethyl (2-(4-chlorophenyl)-3-phenylcyclopropane-1-carboxylate (**11**) (mixture of *syn* and *anti* isomers)

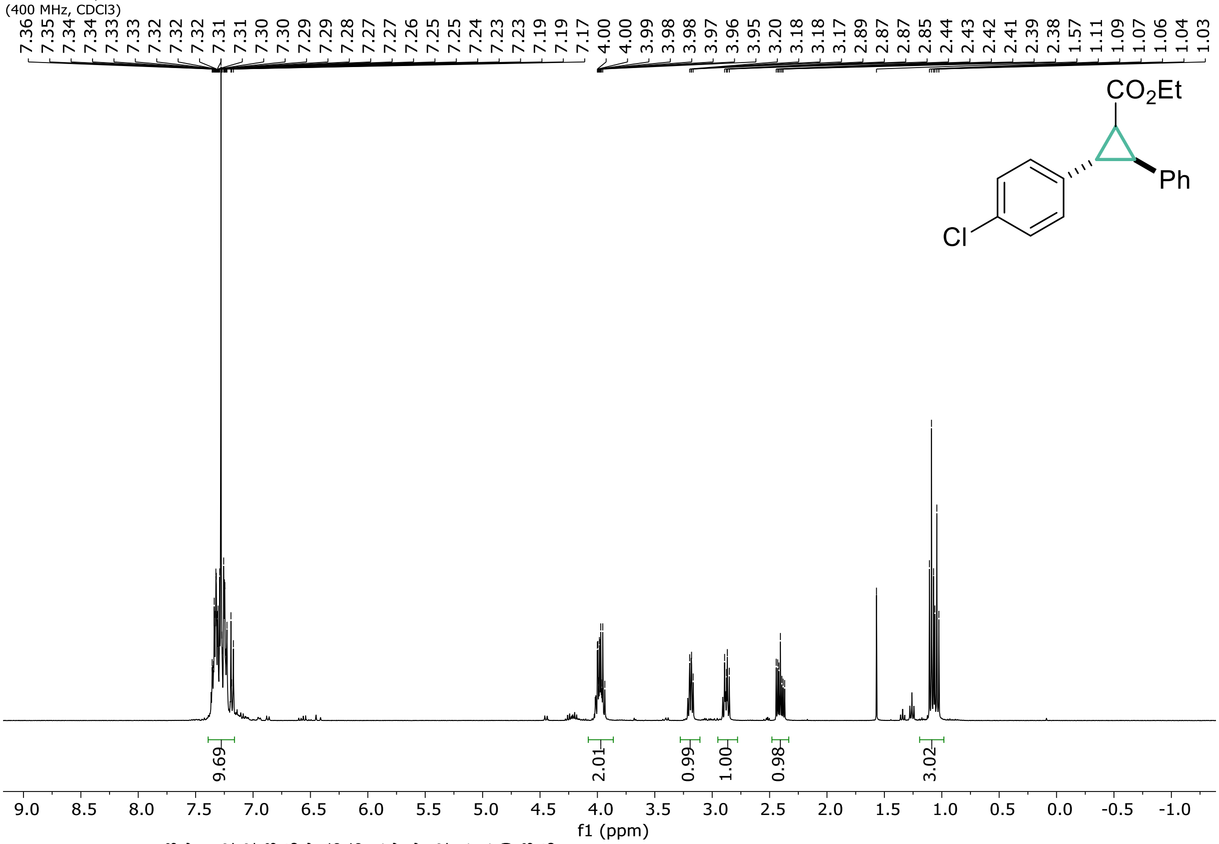

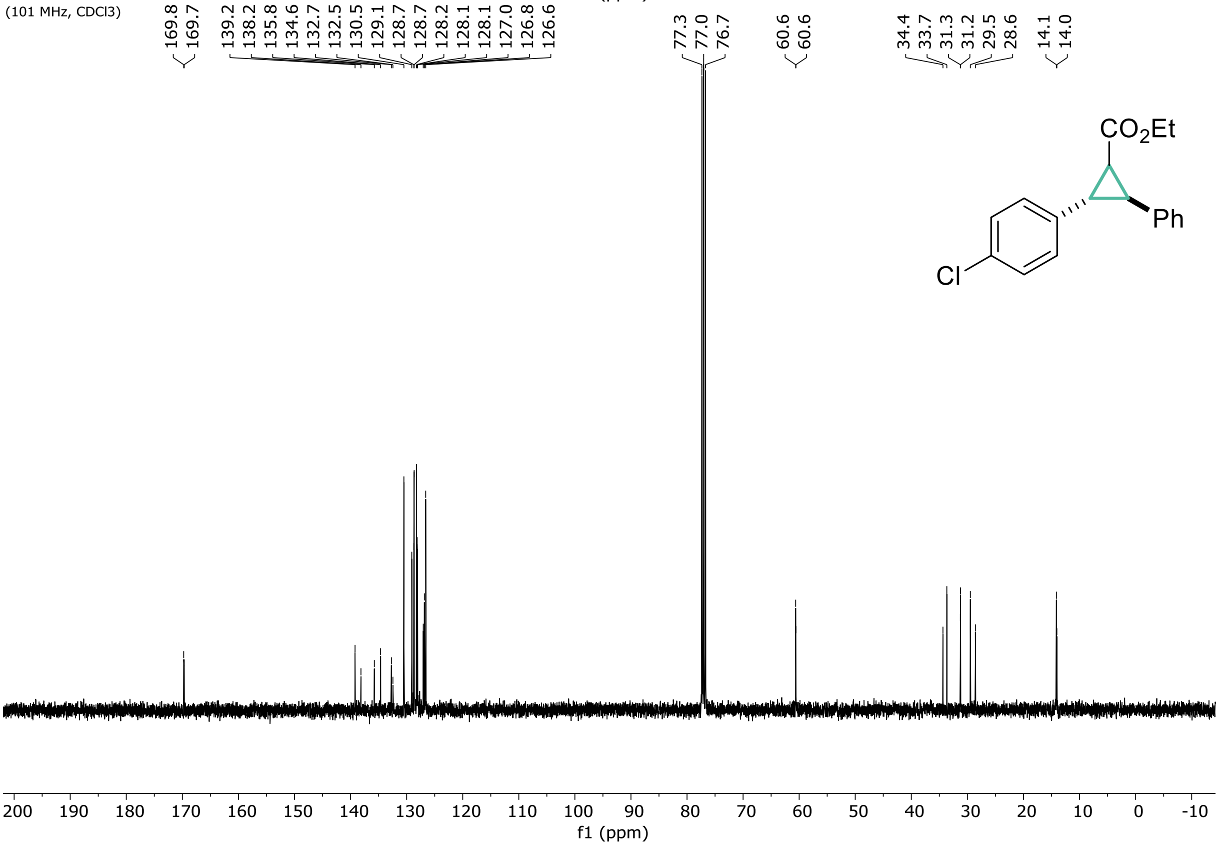

Ethyl 2-phenyl-3-(*p*-tolyl)cyclopropane-1-carboxylate (**12**) (mixture of *syn* and *anti* isomers 1:1.2)

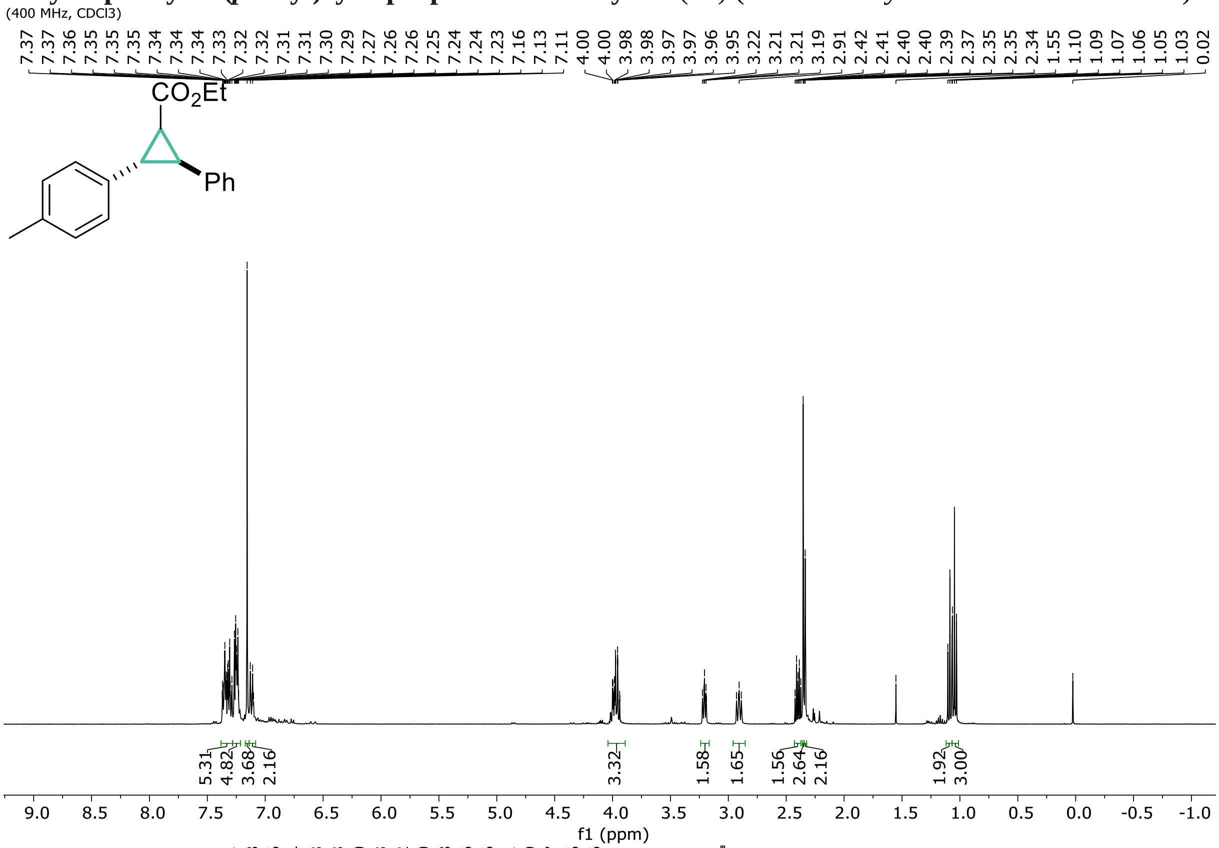

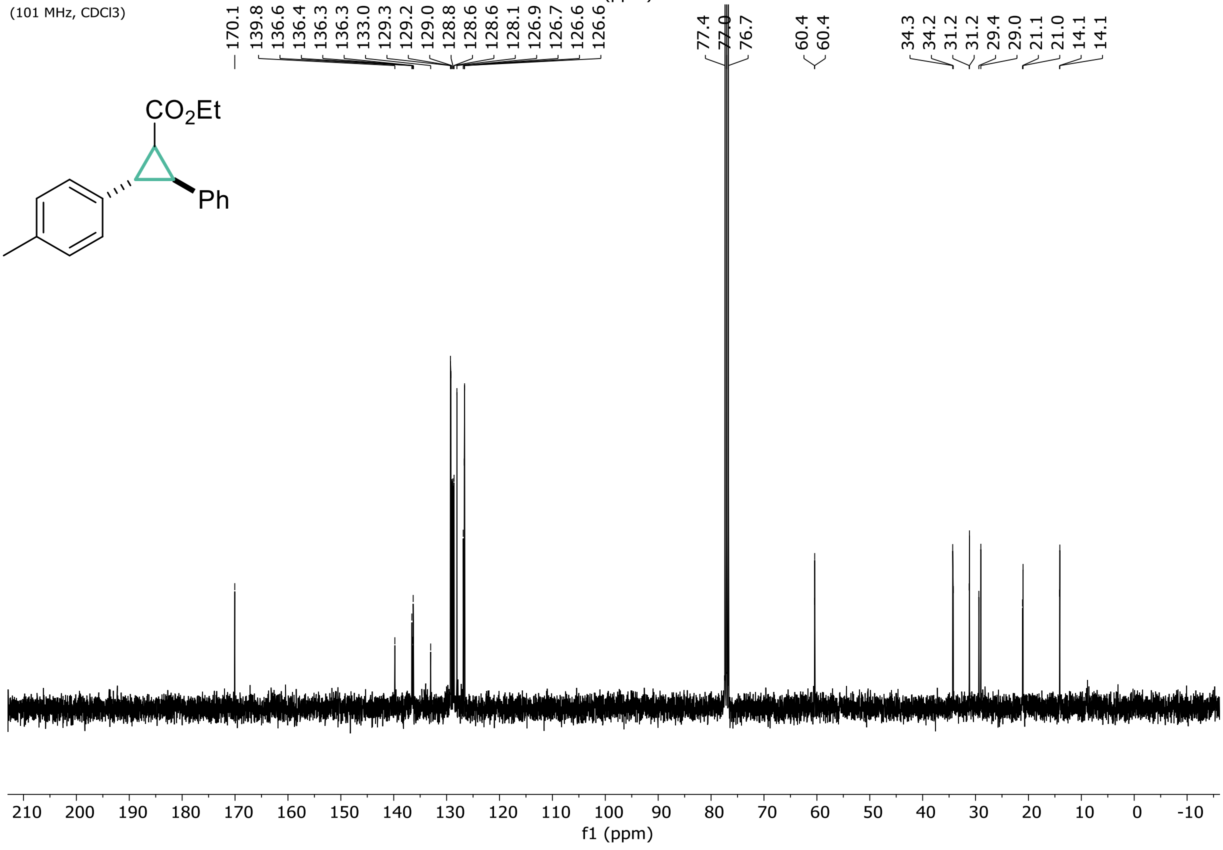

Ethyl 2-(4-methoxyphenyl)-3-methylcyclopropane-1-carboxylate (1,2-*syn*-13)

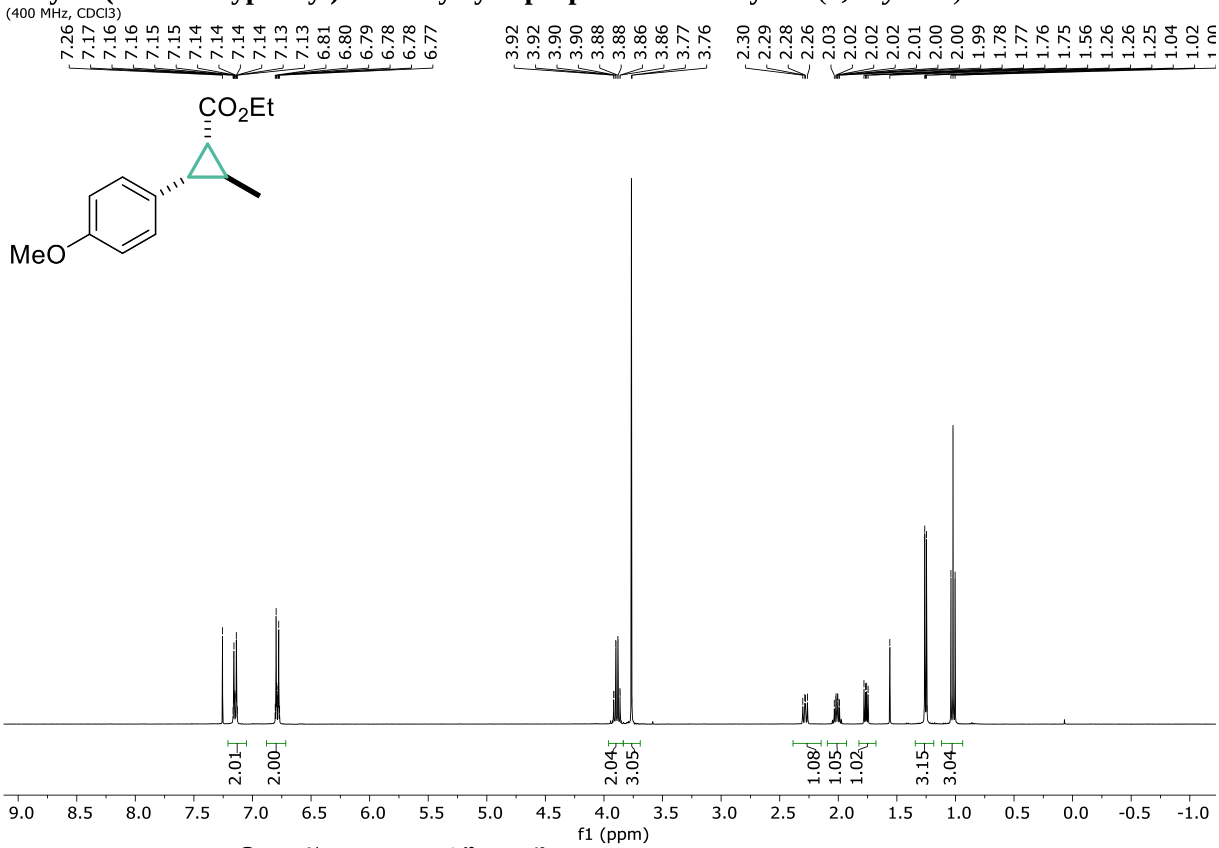

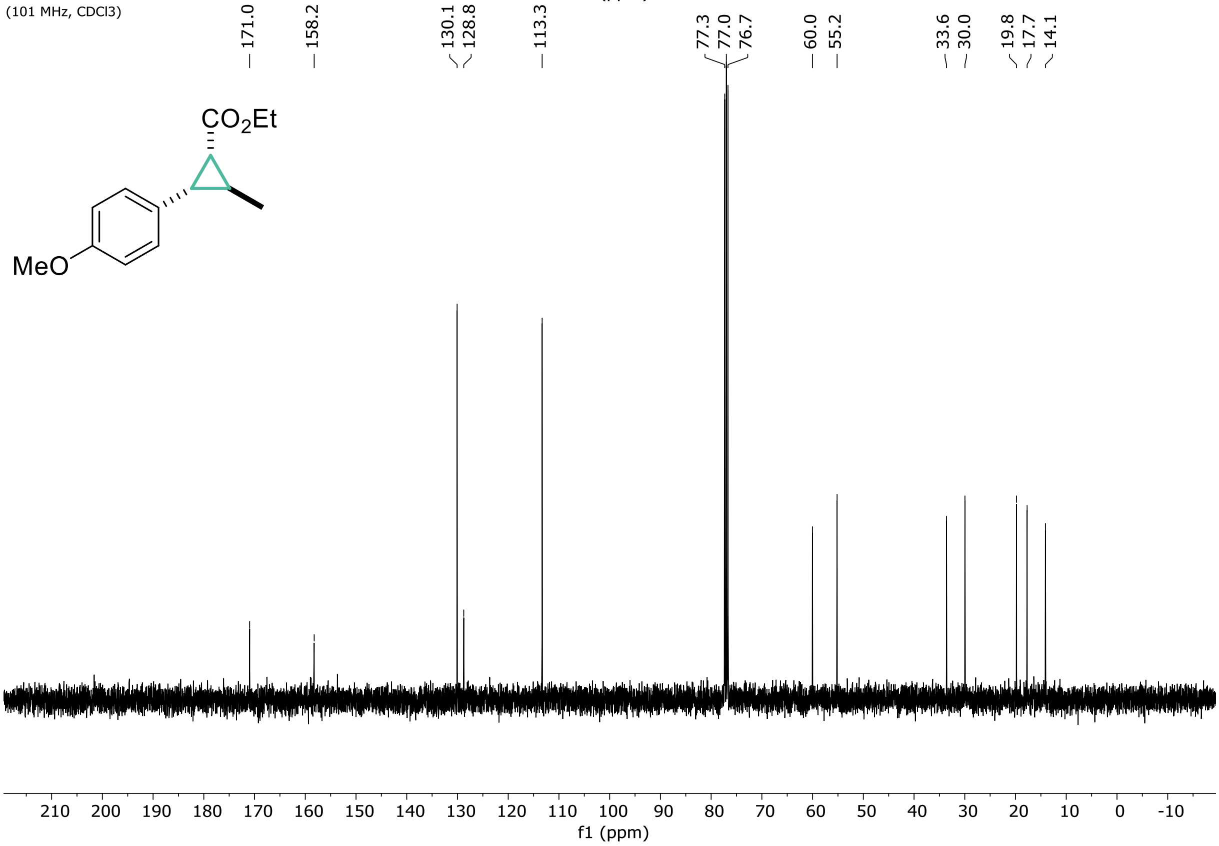

Ethyl 2-(4-methoxyphenyl)-3-methylcyclopropane-1-carboxylate (**1,2-*anti*-13**) + traces of isomer *syn*

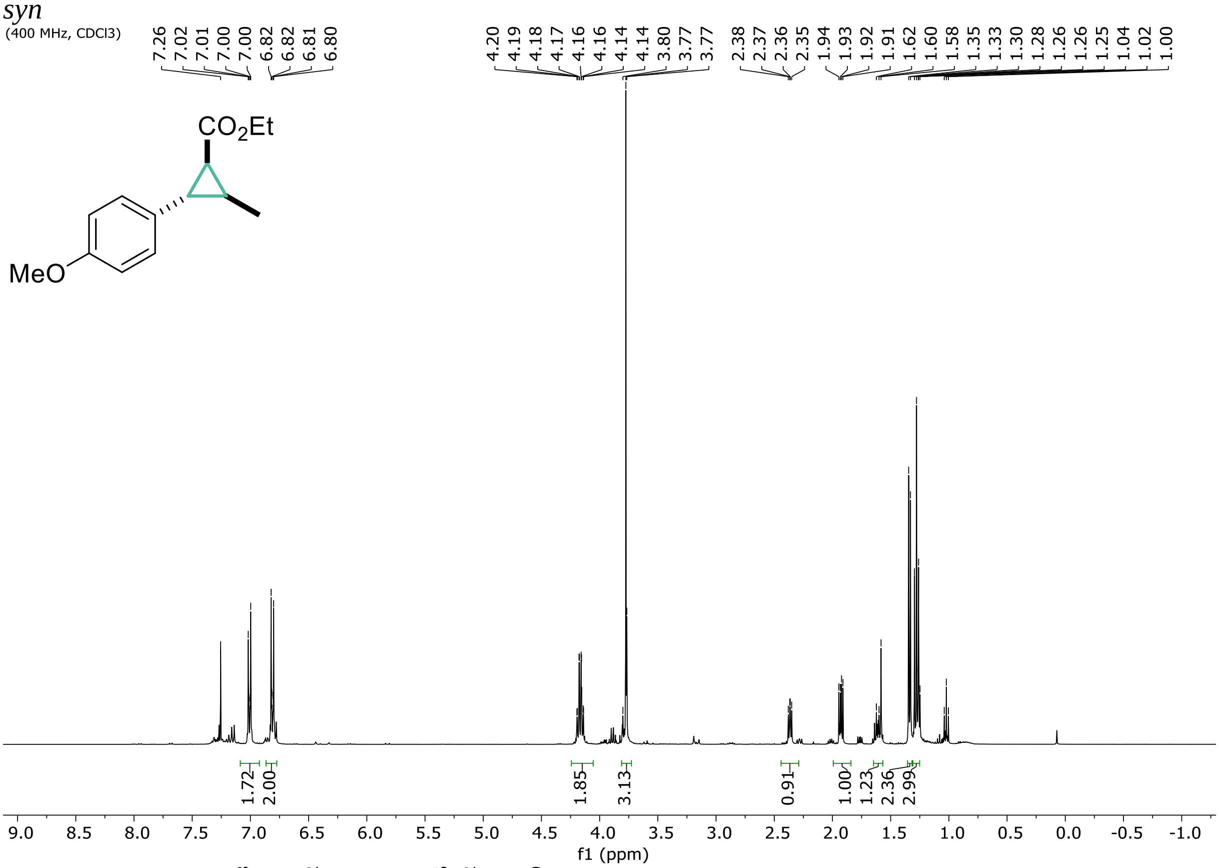

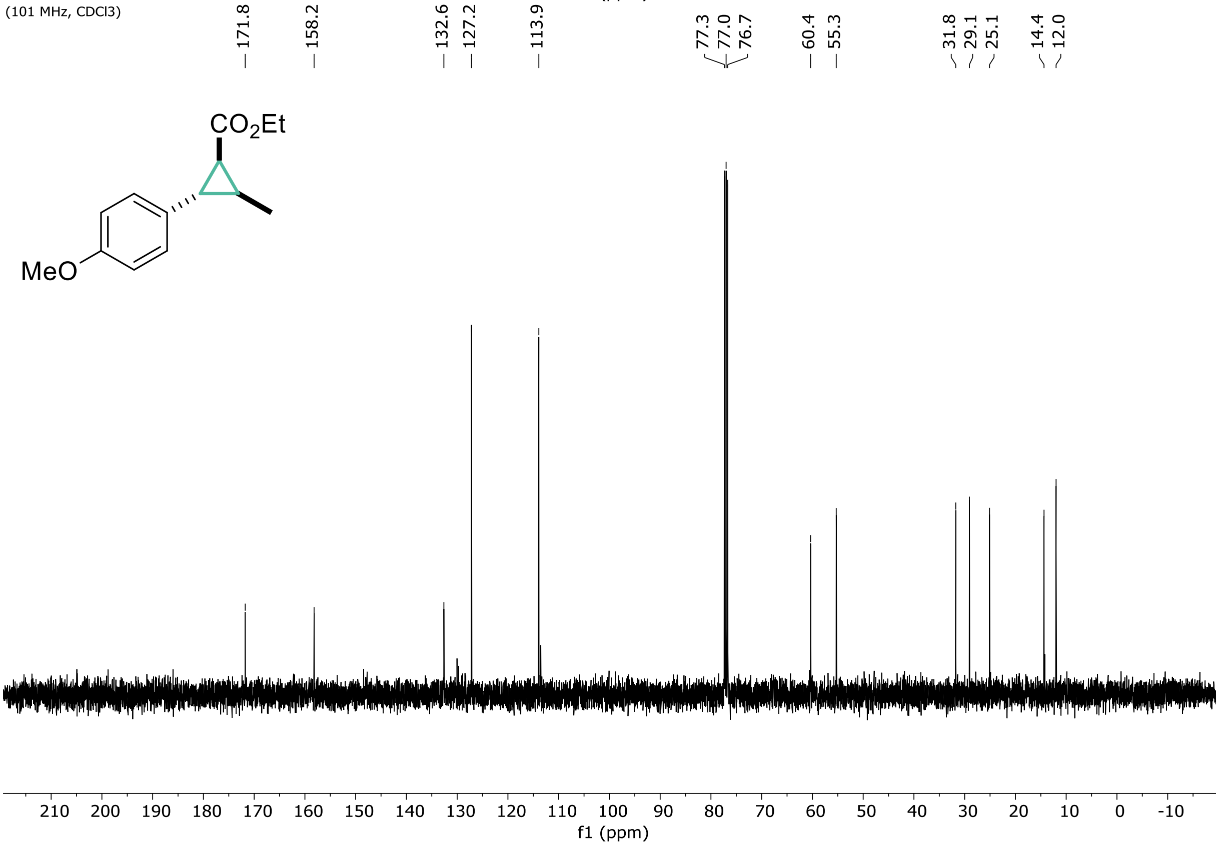

Ethyl 2-methyl-3-phenylcyclopropane-1-carboxylate (1,2-*syn*-14)

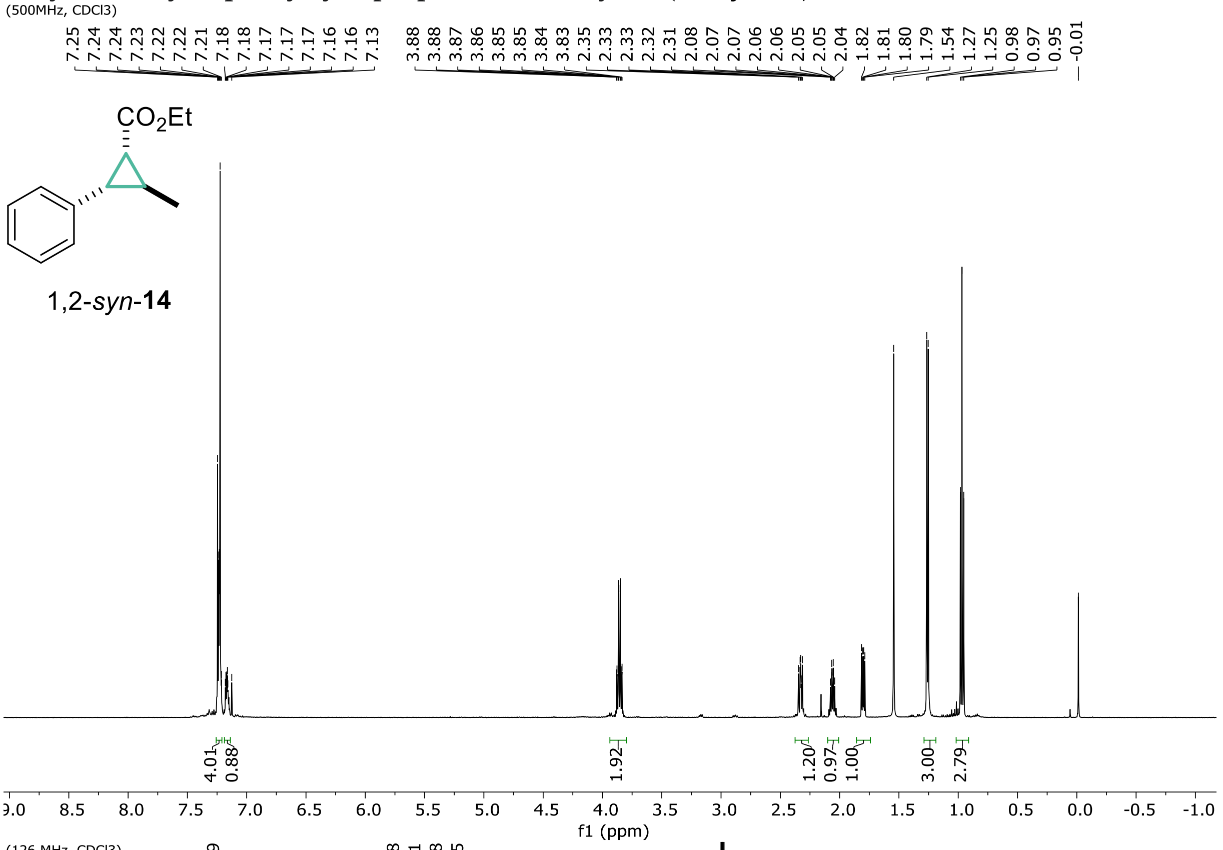

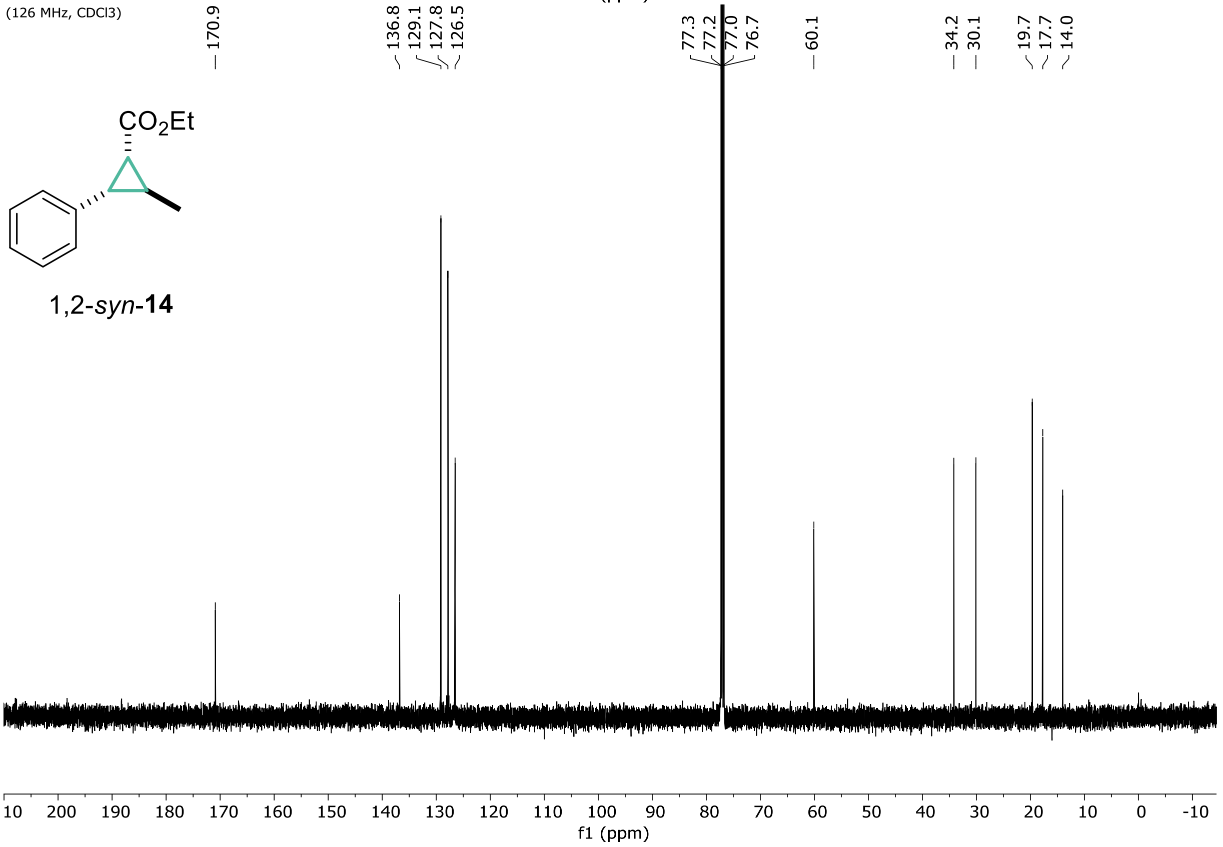

Ethyl 2-methyl-3-phenylcyclopropane-1-carboxylate (1,2-*anti*-14) + proton-migrated by-prodyct

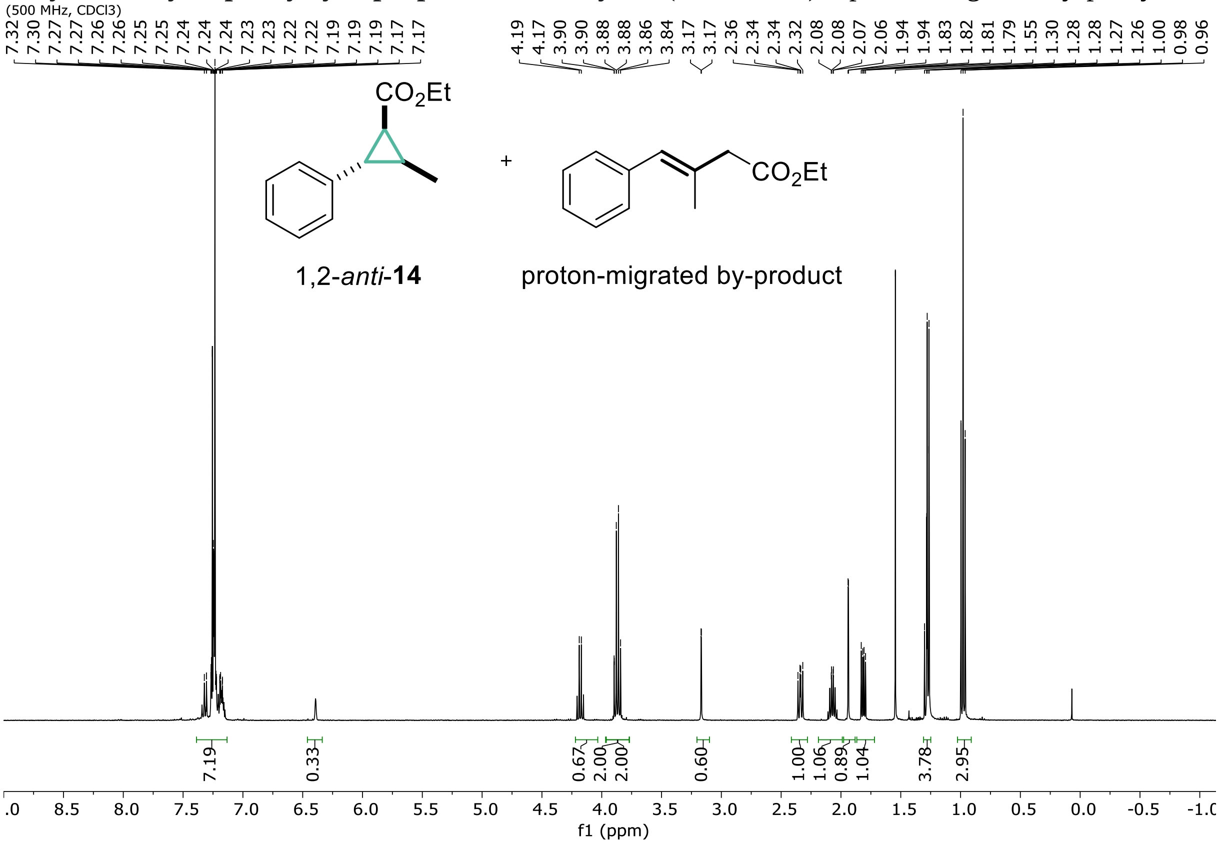

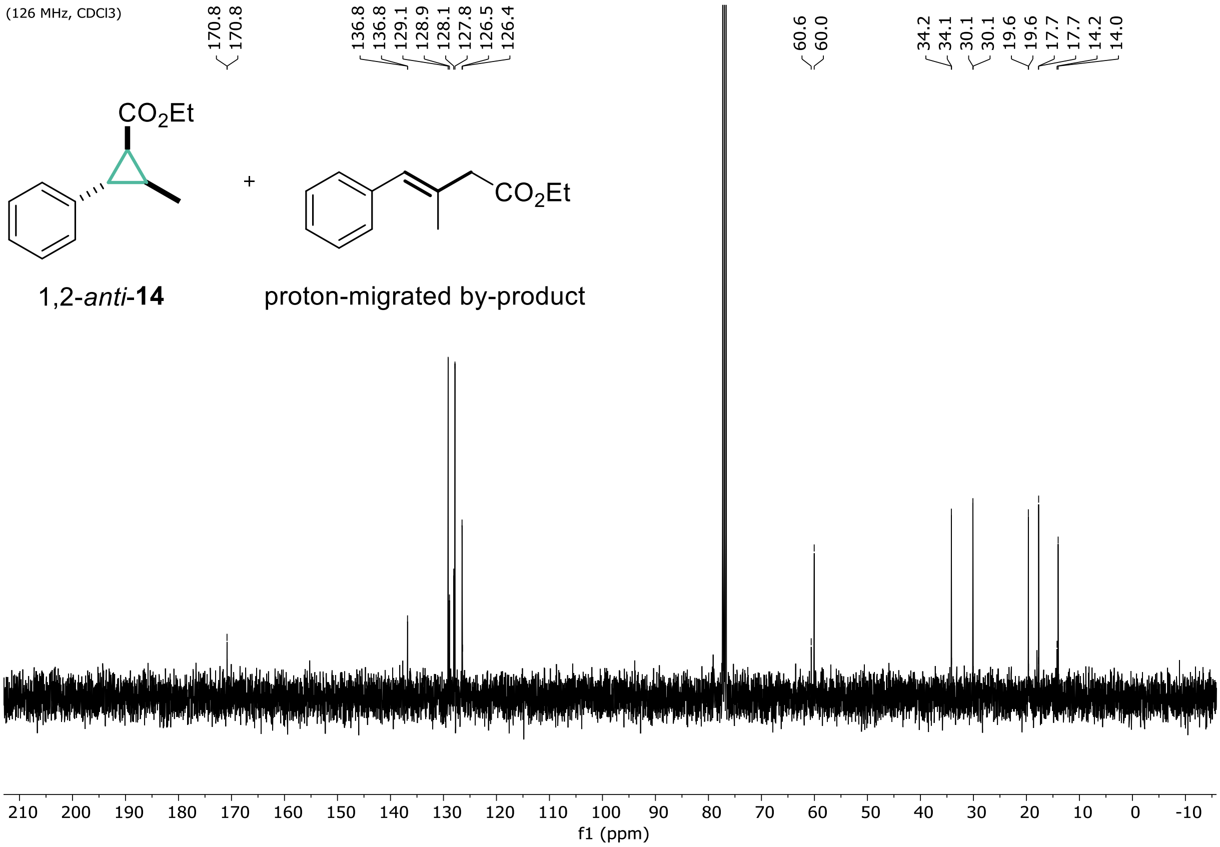

Ethyl 1a,2,3,7b-tetrahydro-1H-cyclopropa[a]naphthalene-1-carboxylate (1,2-*syn*-15) + traces of 1,2-*anti*-15

(400 MHz, CDCl3)

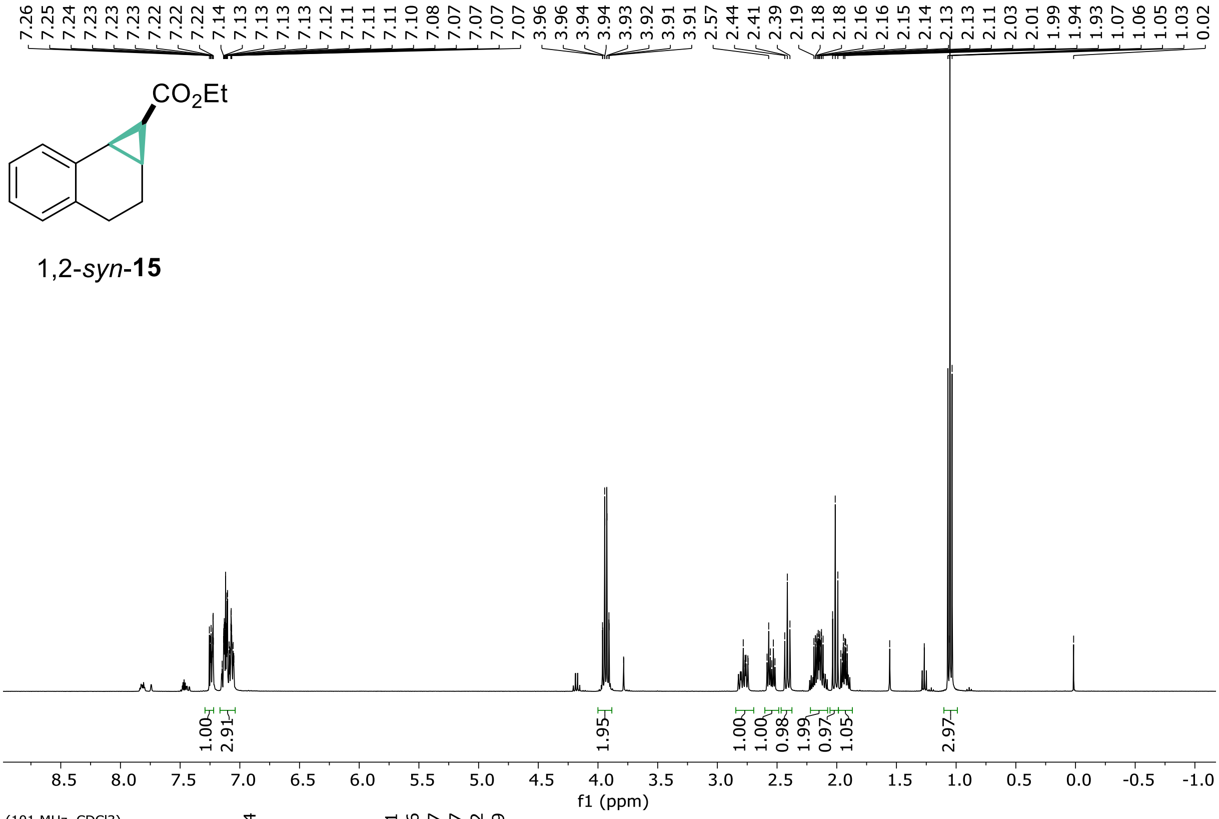

(101 MHz, CDCl3)

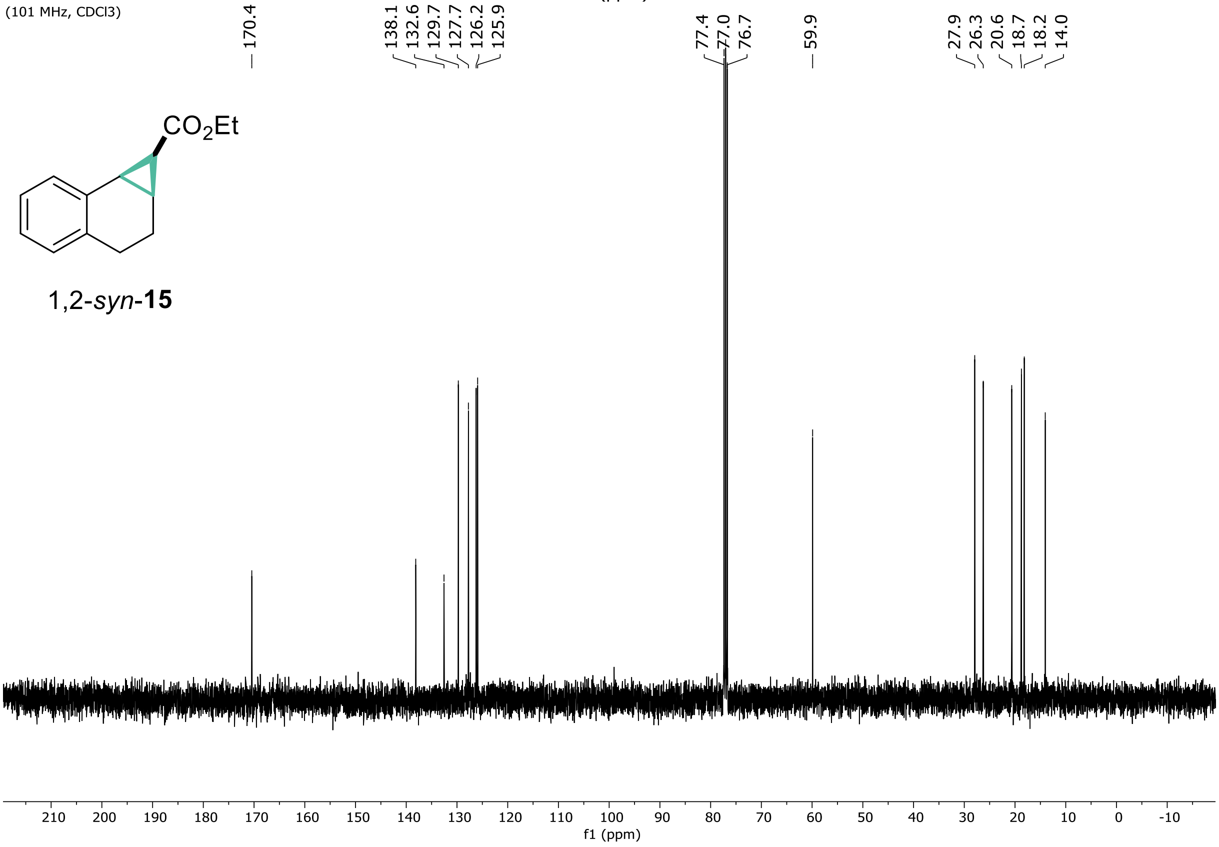

Ethyl 1a,2,3,7b-tetrahydro-1H-cyclopropa[a]naphthalene-1-carboxylate (**15**) (mixture of two 1,2-*anti*-15 + proton-migrated by-product)

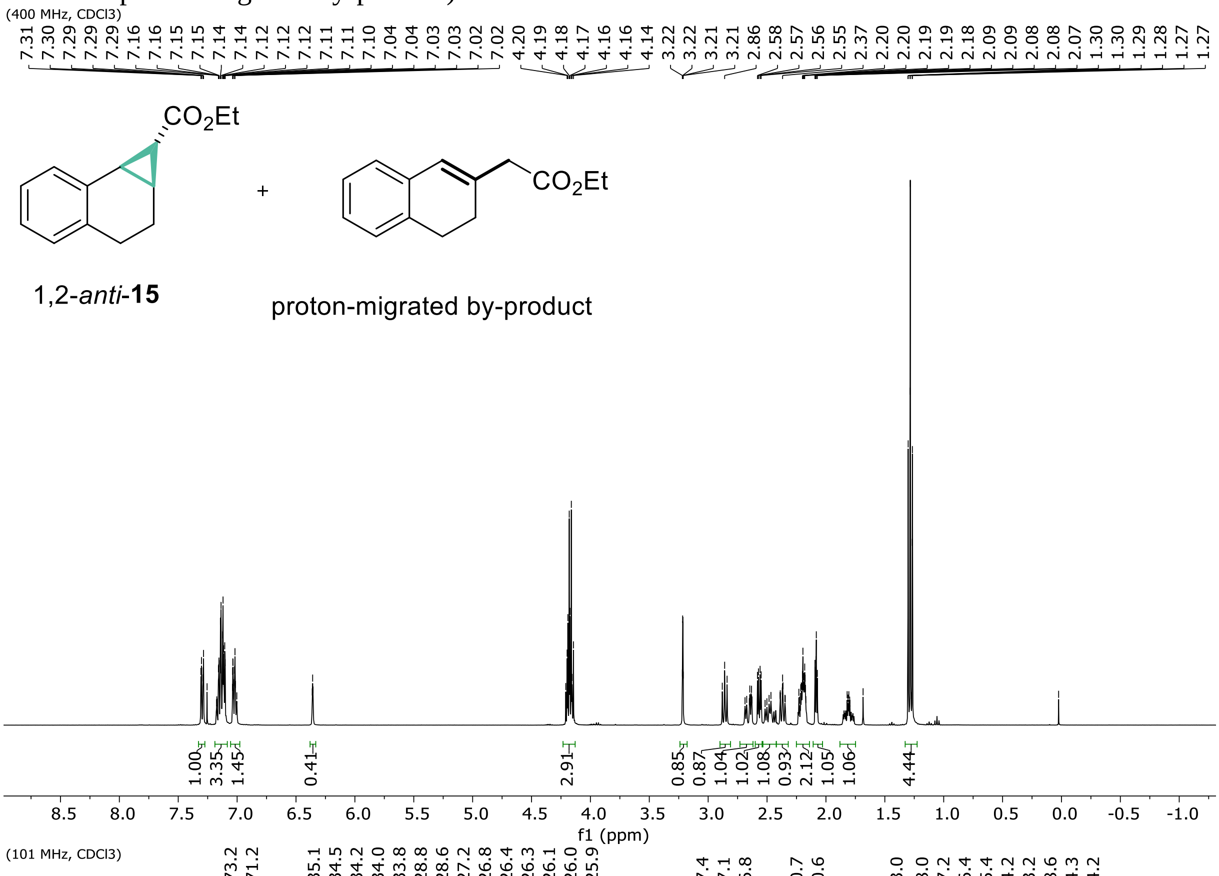

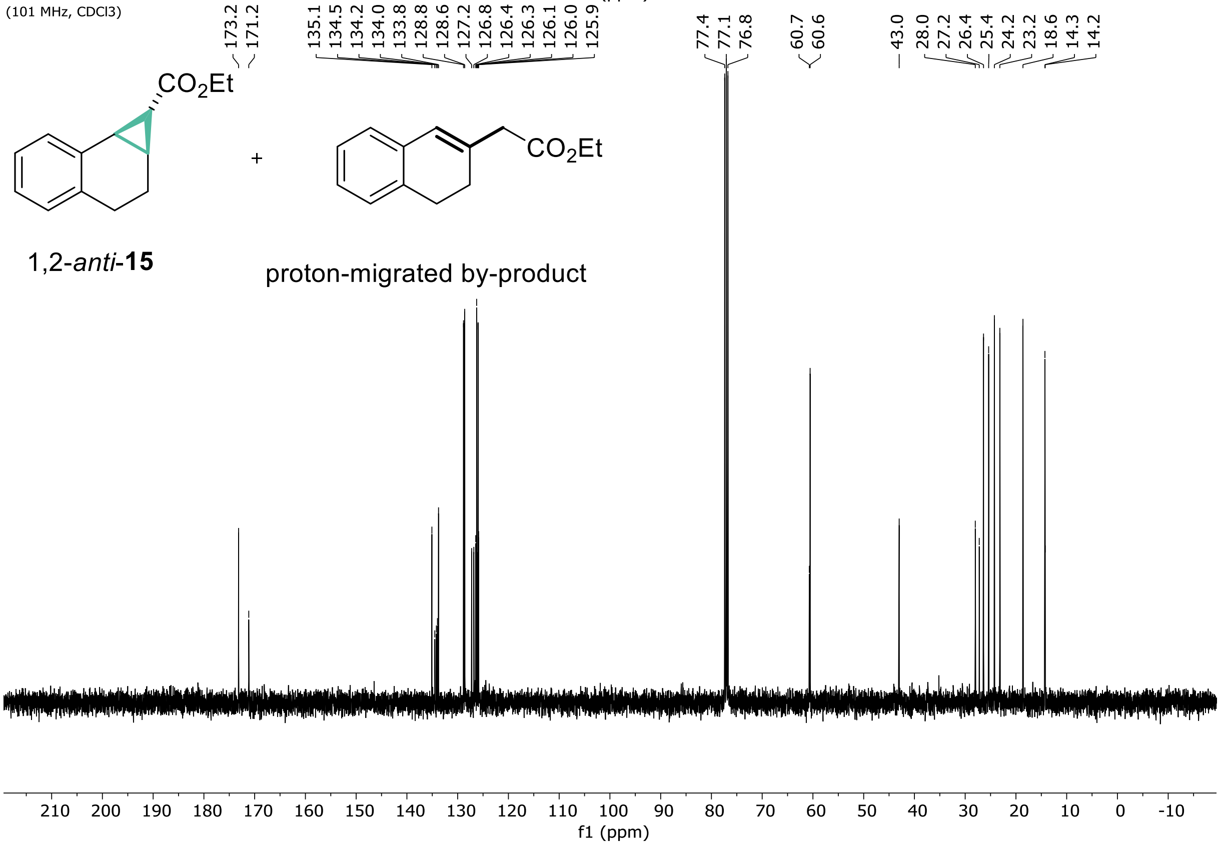

***Tert*-butyl 2,3-diphenylcyclopropane-1-carboxylate (17)**

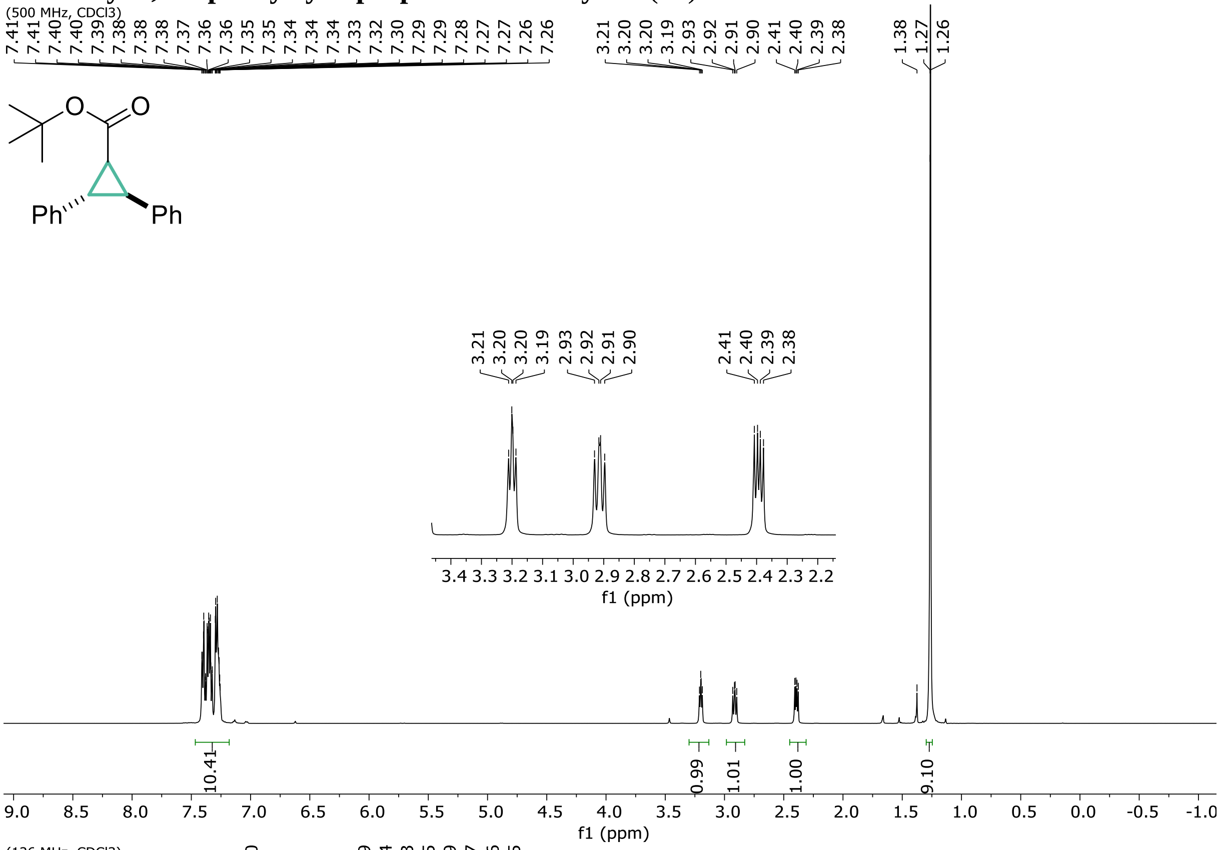

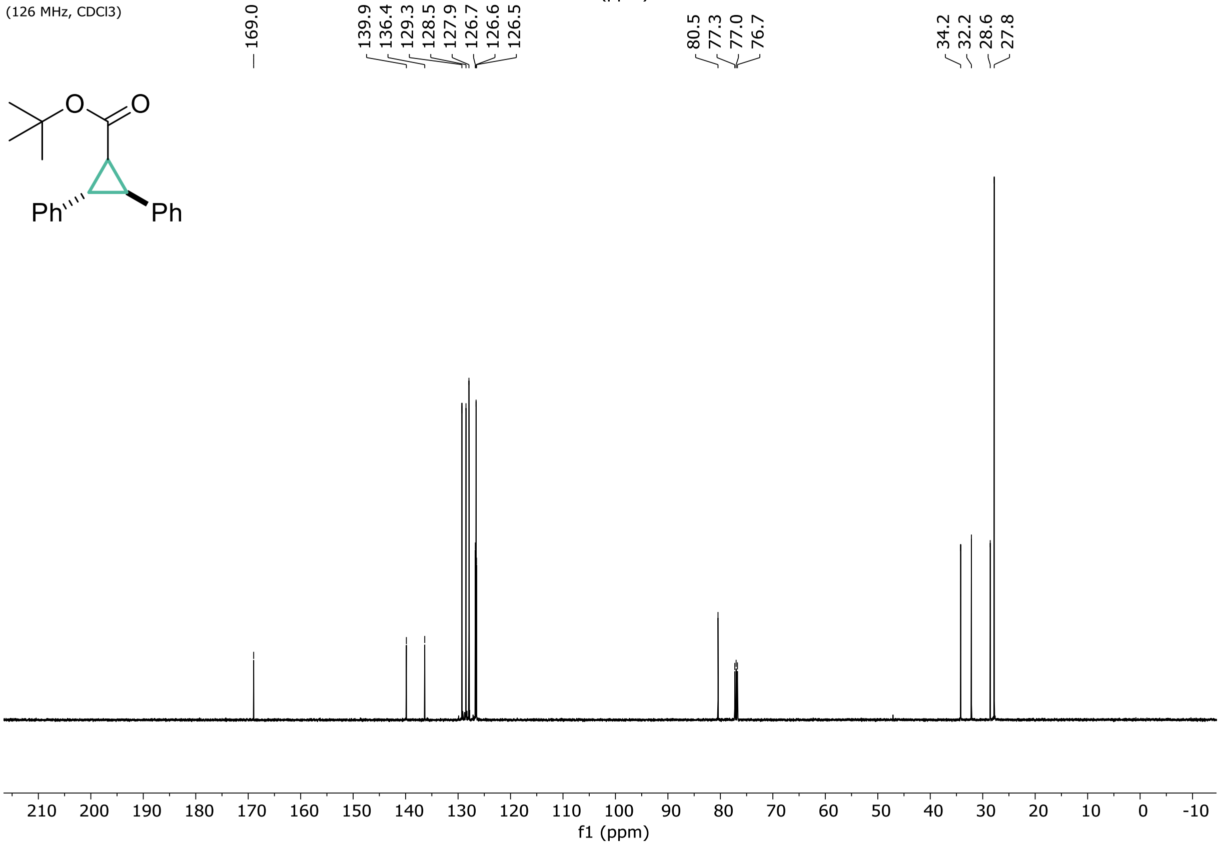

3-phenylpropyl 2,3-diphenylcyclopropane-1-carboxylate (18)

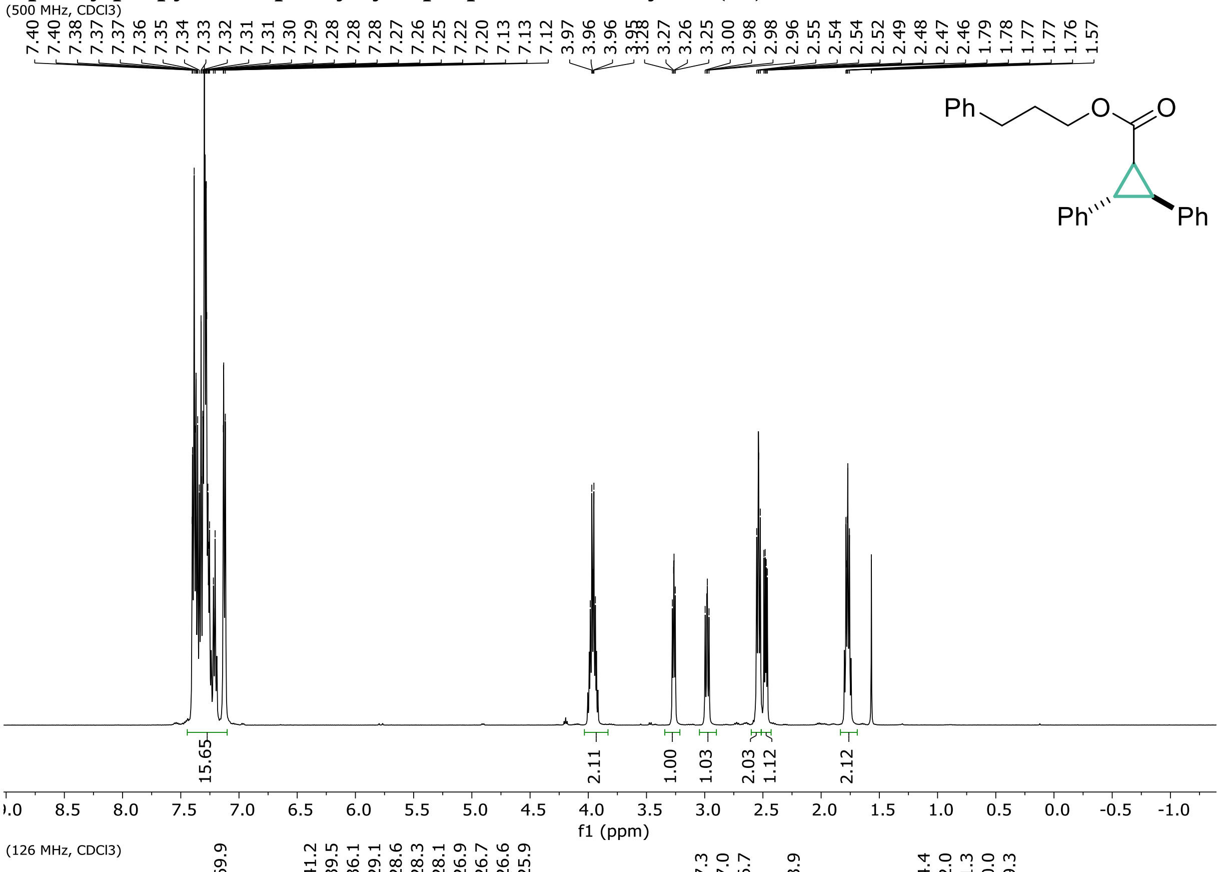

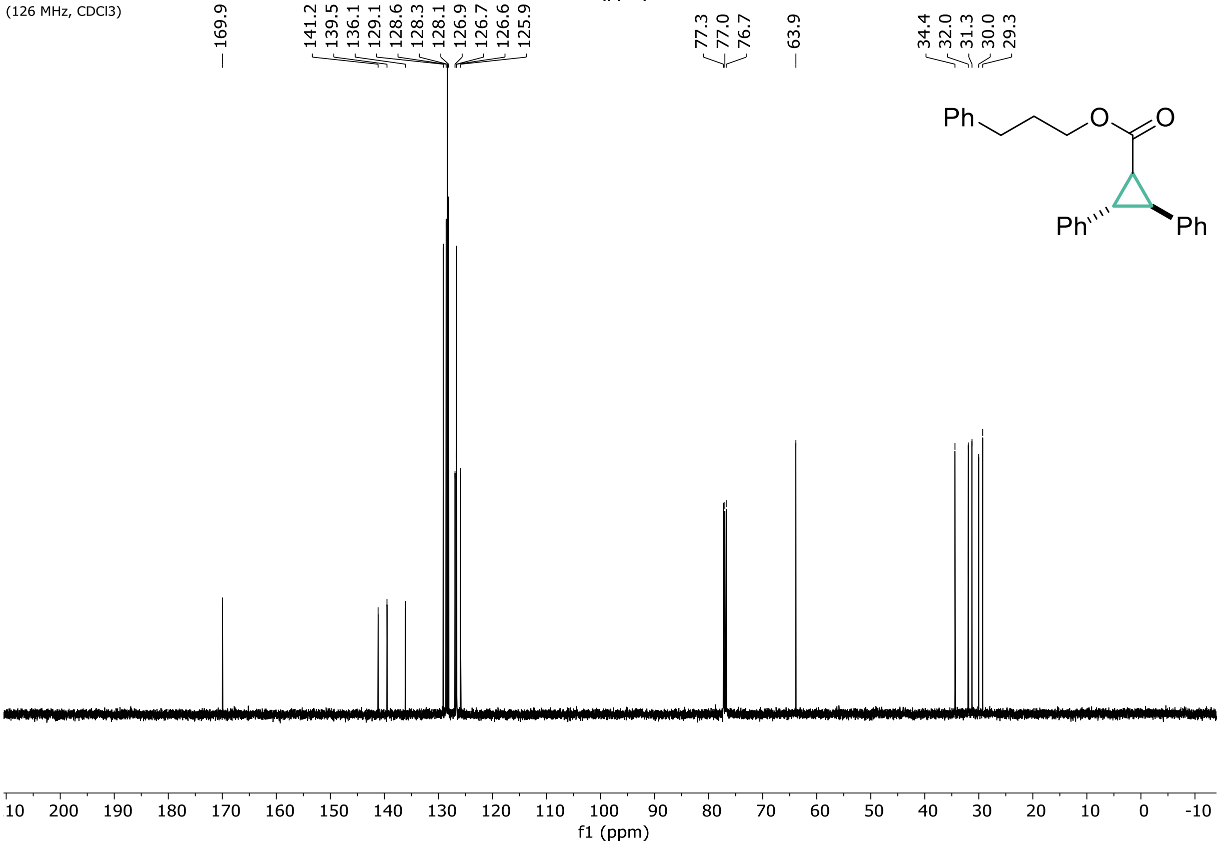

***tert*-Butyl 2,3-diphenylcyclopropane-1-carbonyl)oxy)methyl)pyrrolidine-1-carboxylate (19)**

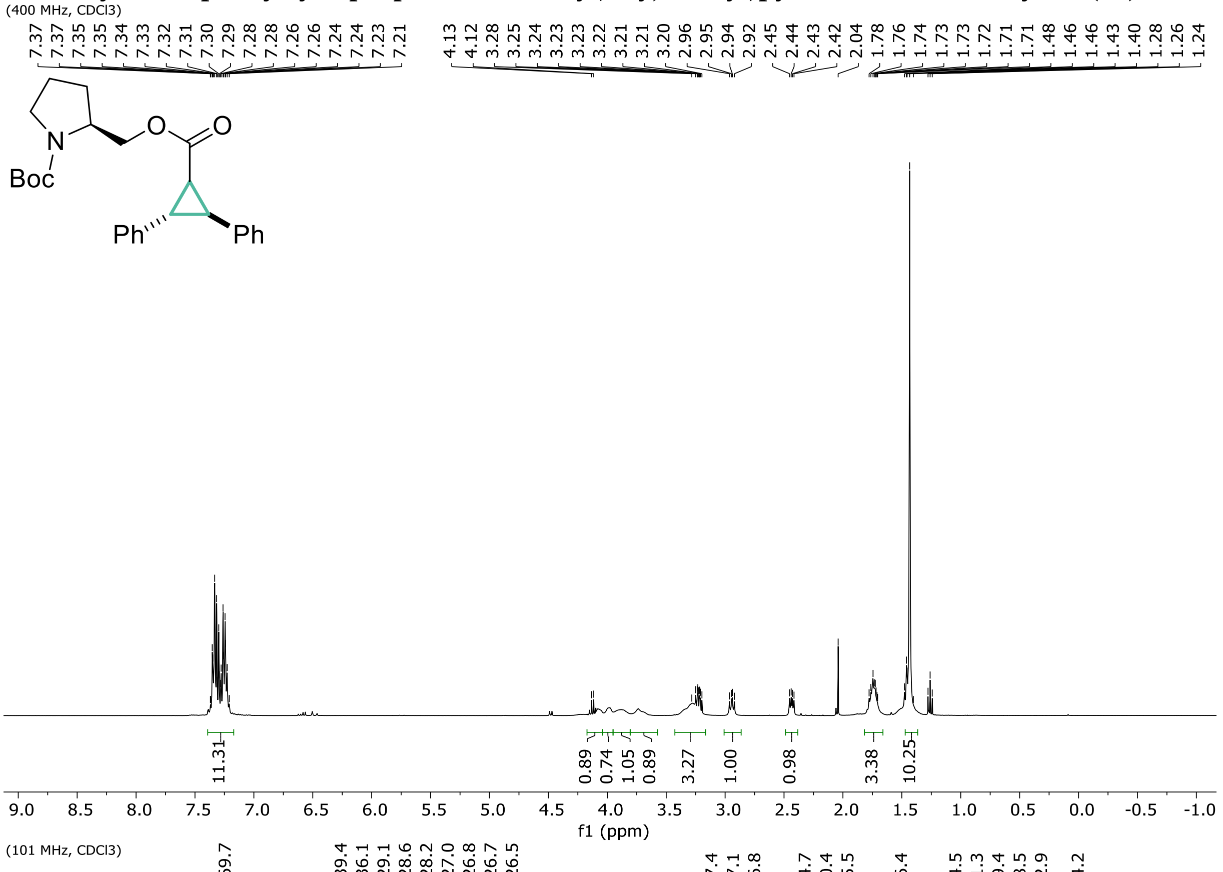

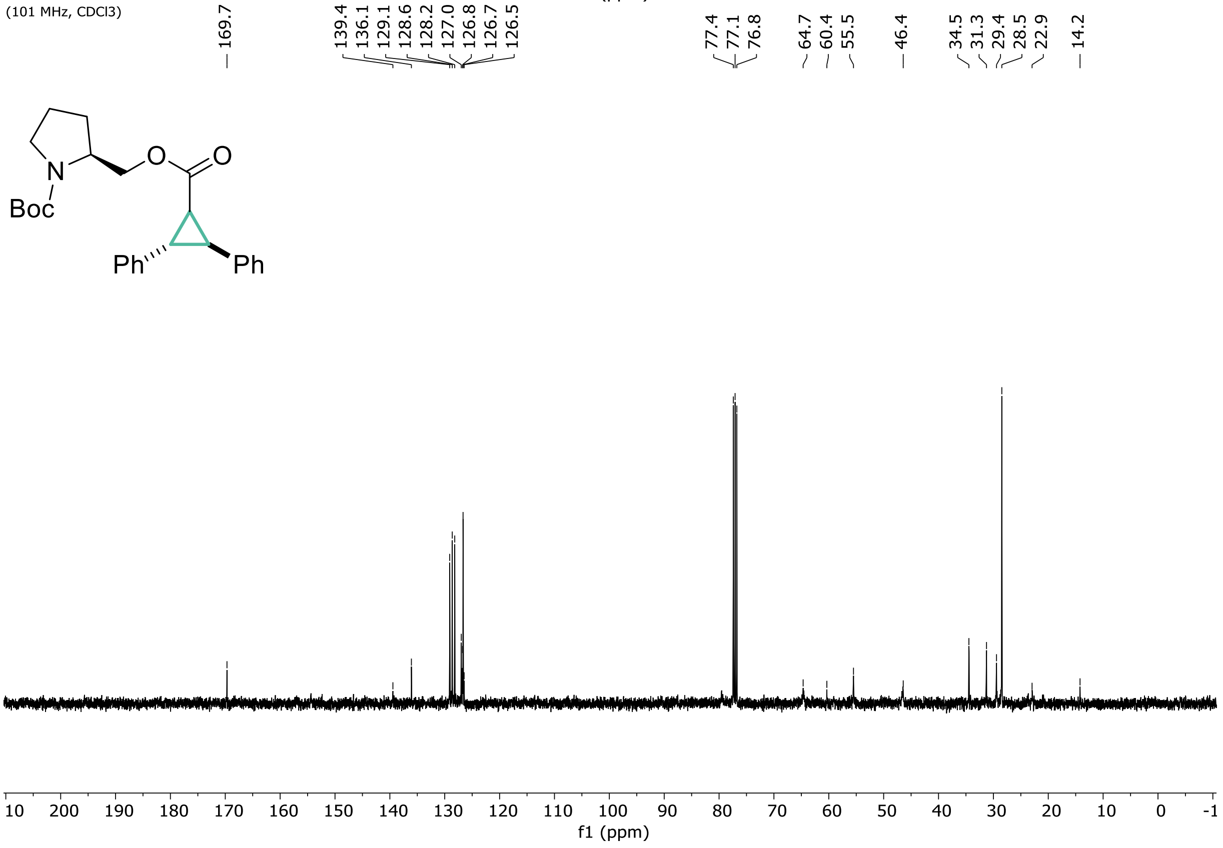

2-((*tert*-butoxycarbonyl)amino)-3-phenylpropyl-2,3-diphenylcyclopropane-1-carboxylate (20)

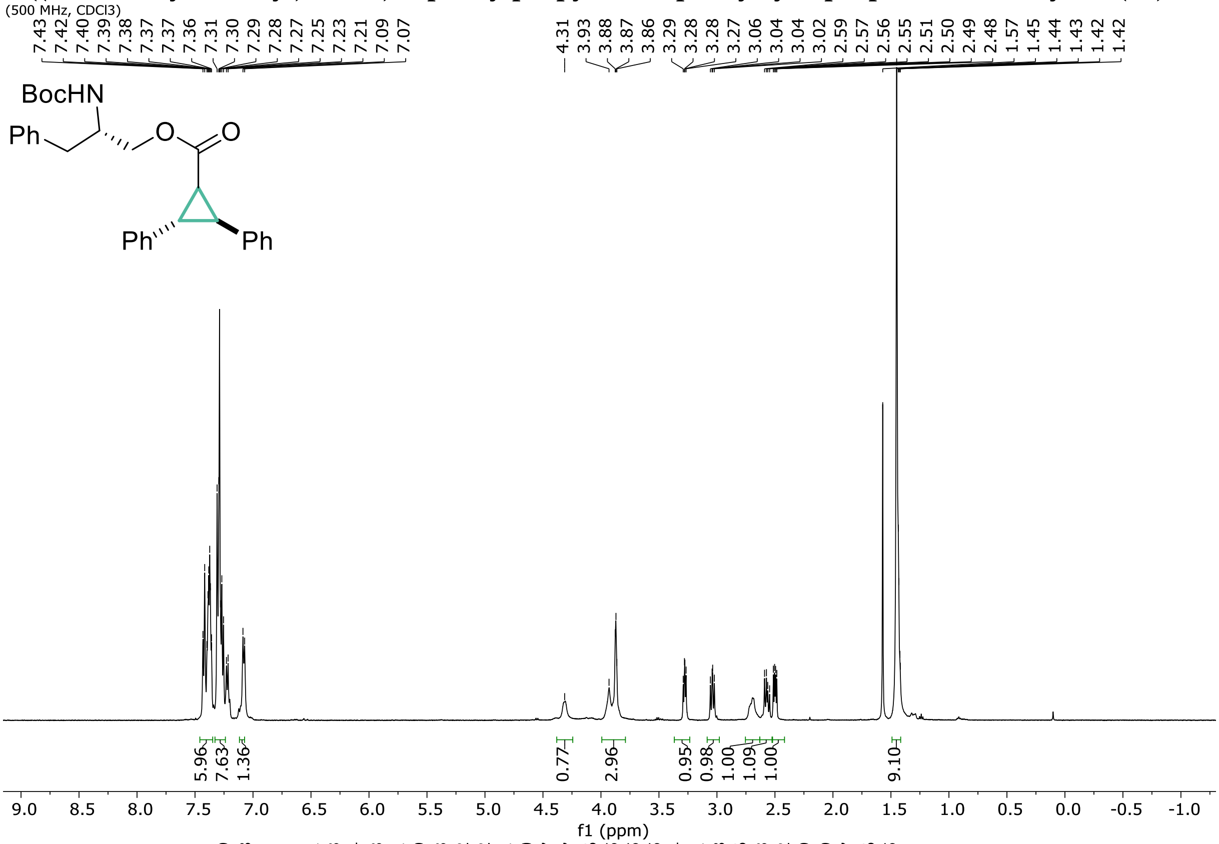

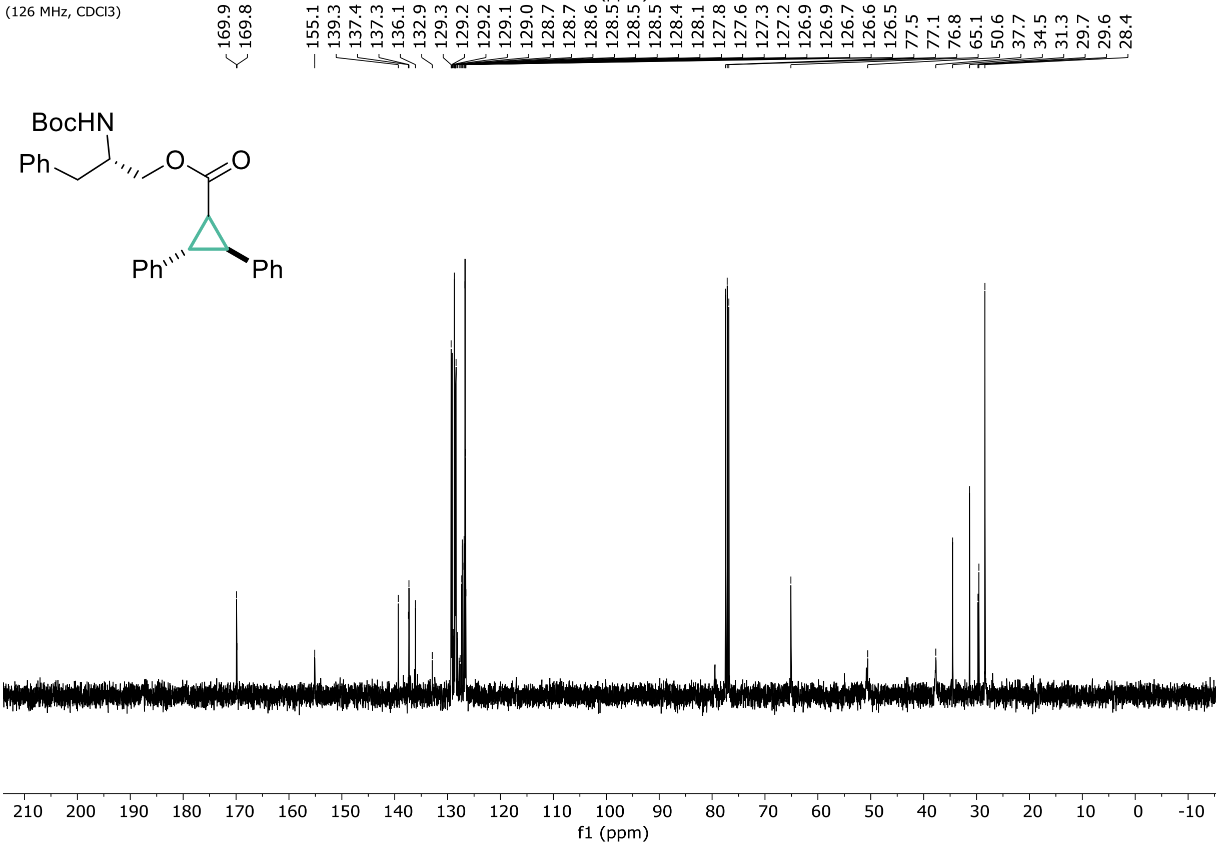

1-2,3-diphenylcyclopropyl)-2,2-dimethylpropan-1-one (21)

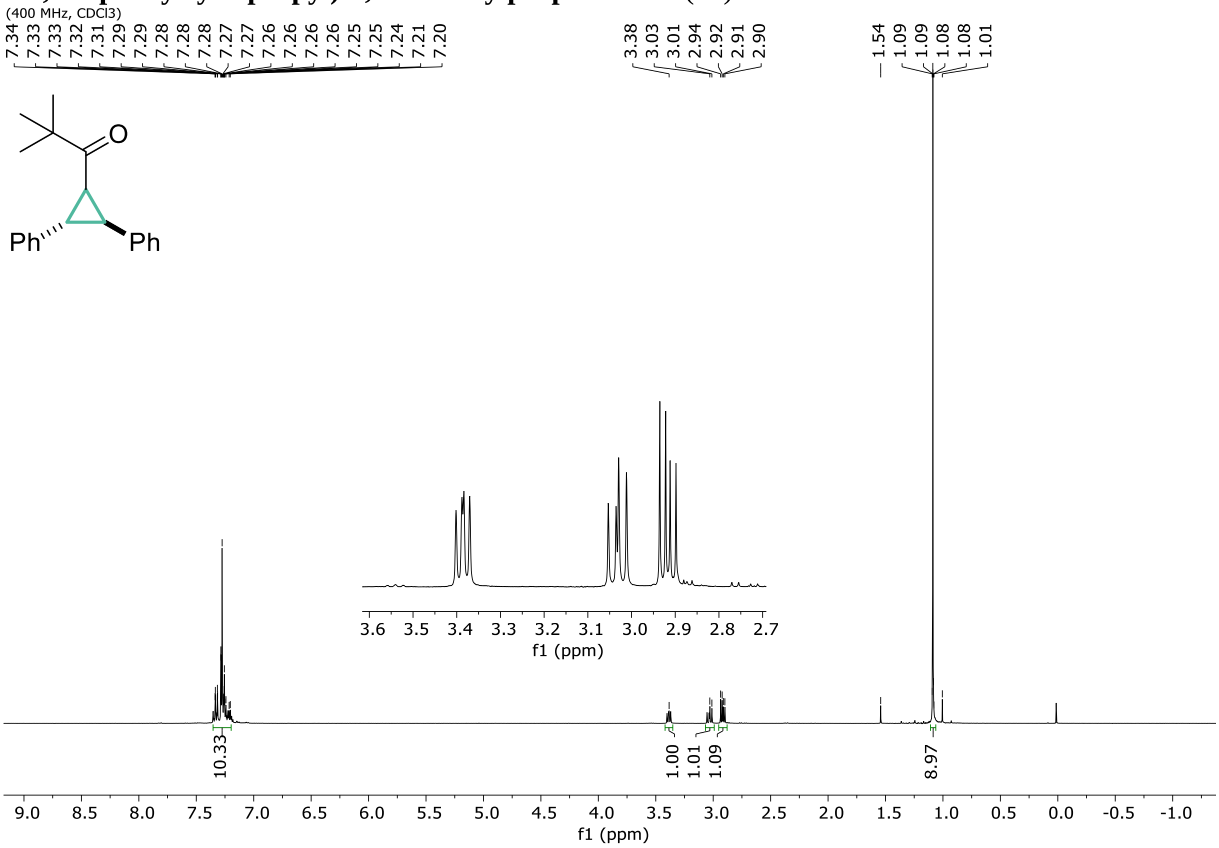

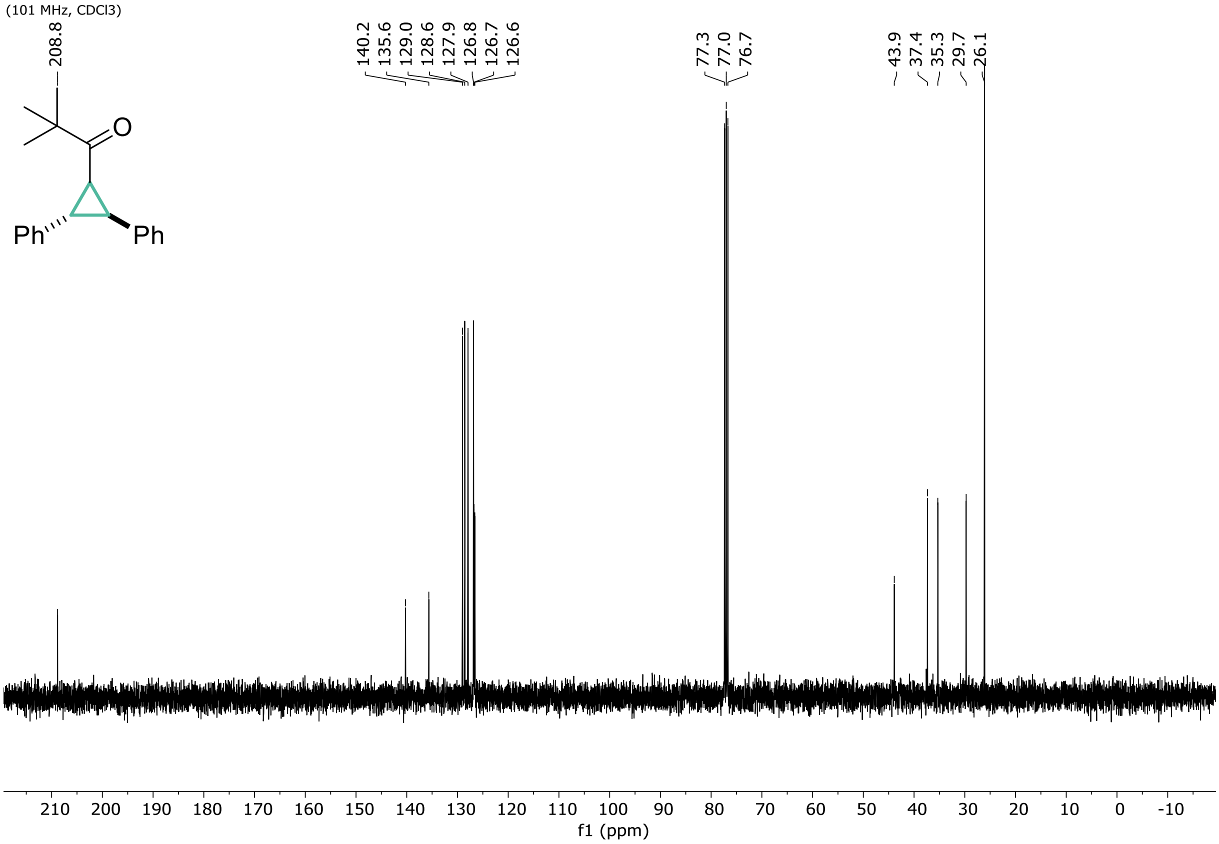

2,3-diphenylcyclopropyl)(phenyl)methanone (22)

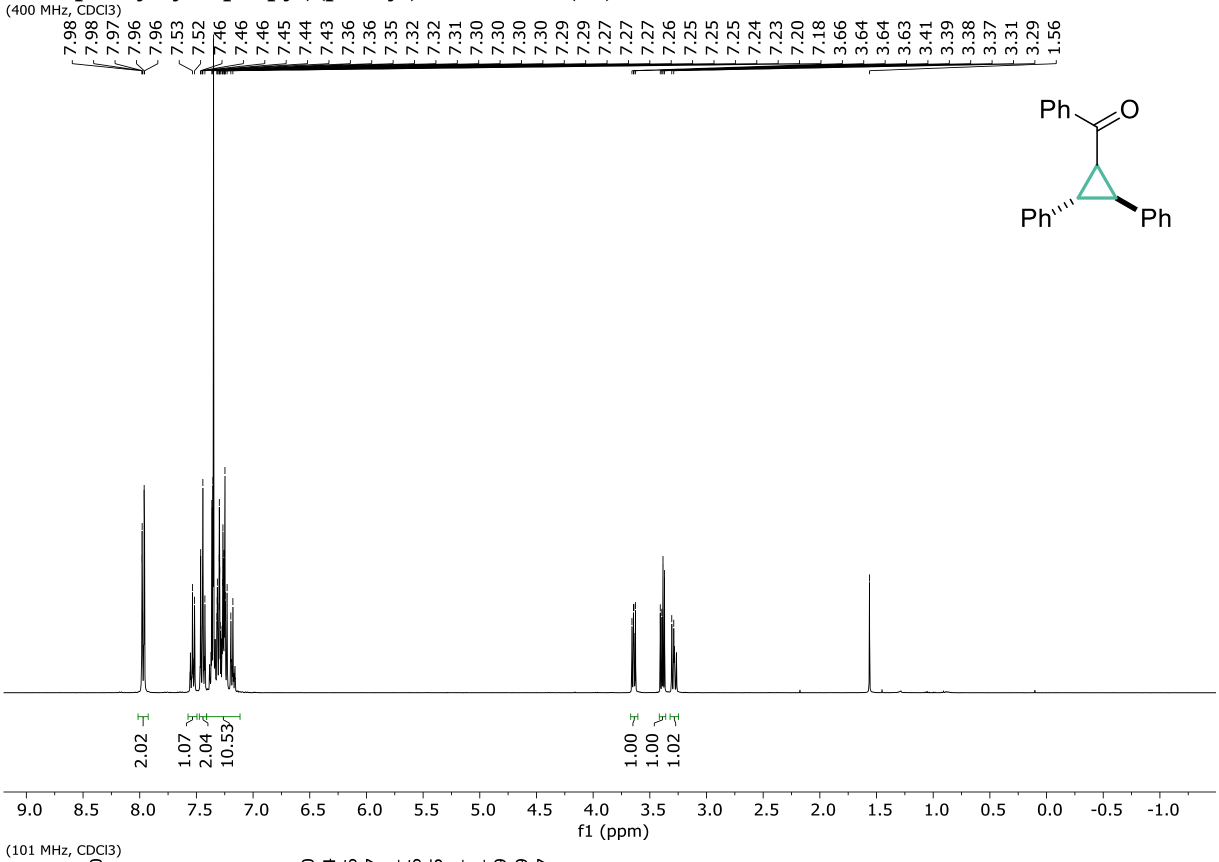

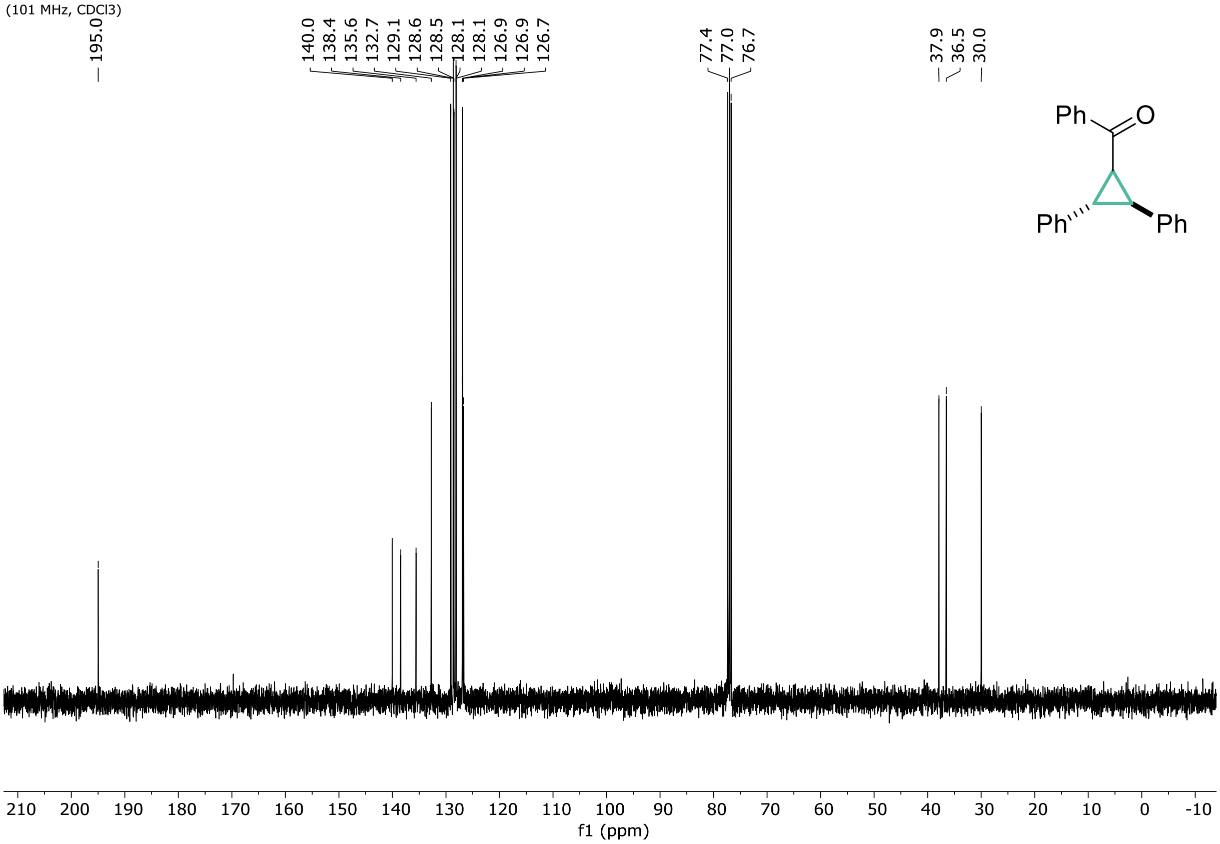

2,3-diphenylcyclopropyl)(4-methoxyphenyl)methanone (23)

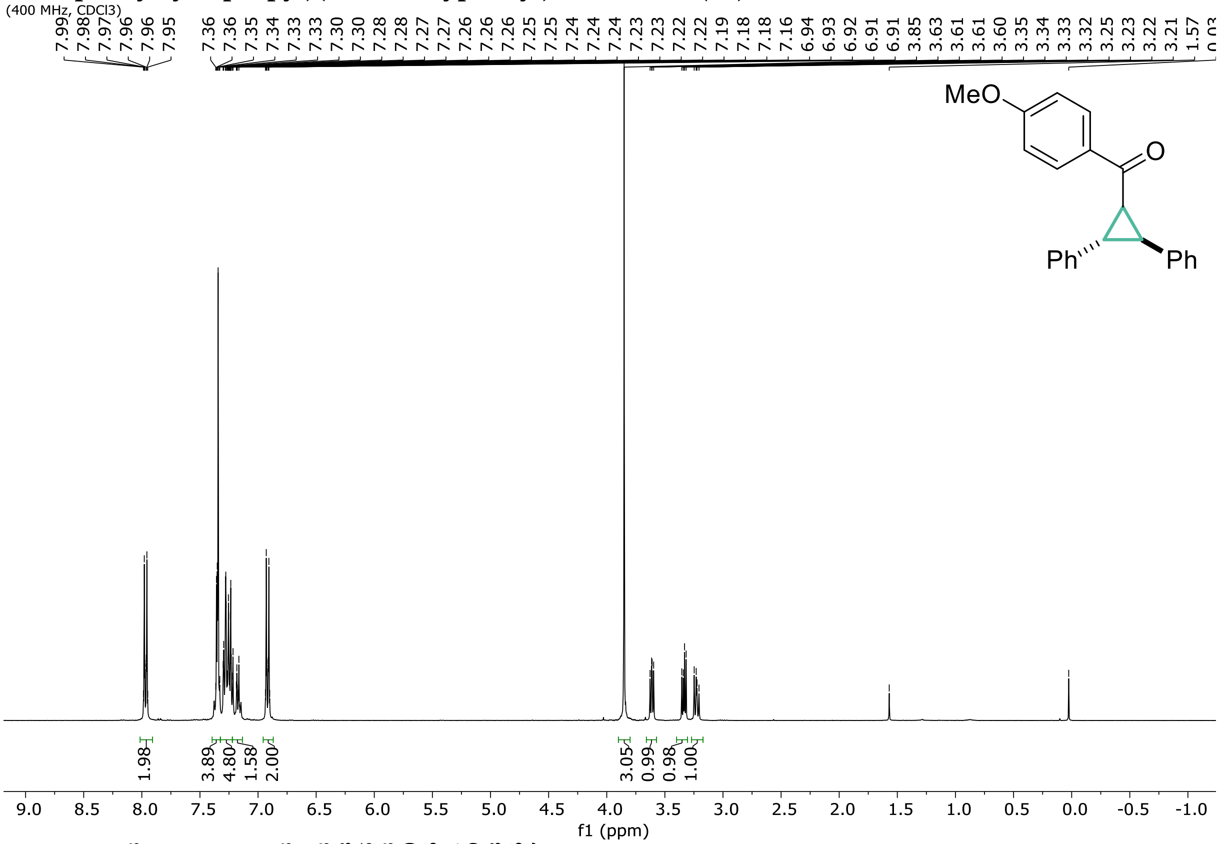

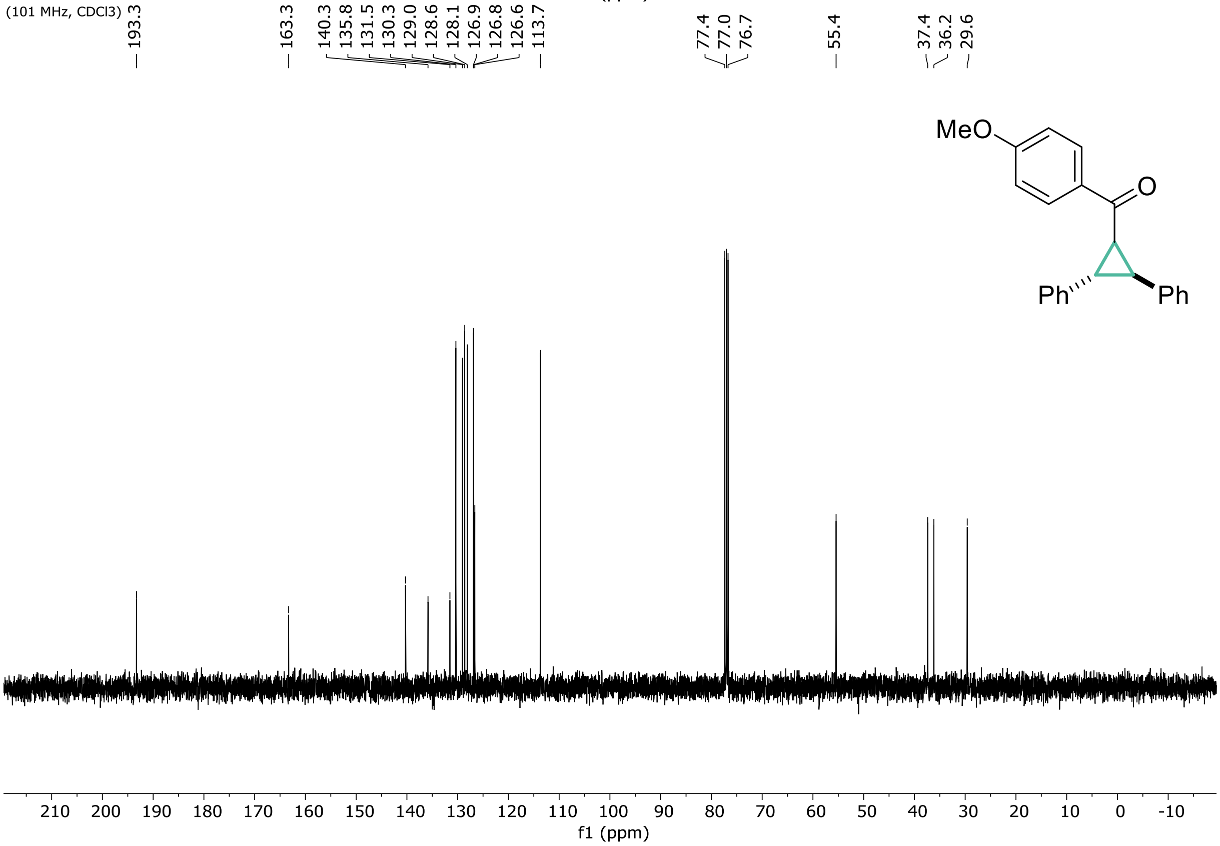

2,3-diphenylcyclopropyl)(4-nitrophenyl)methanone (**24**)

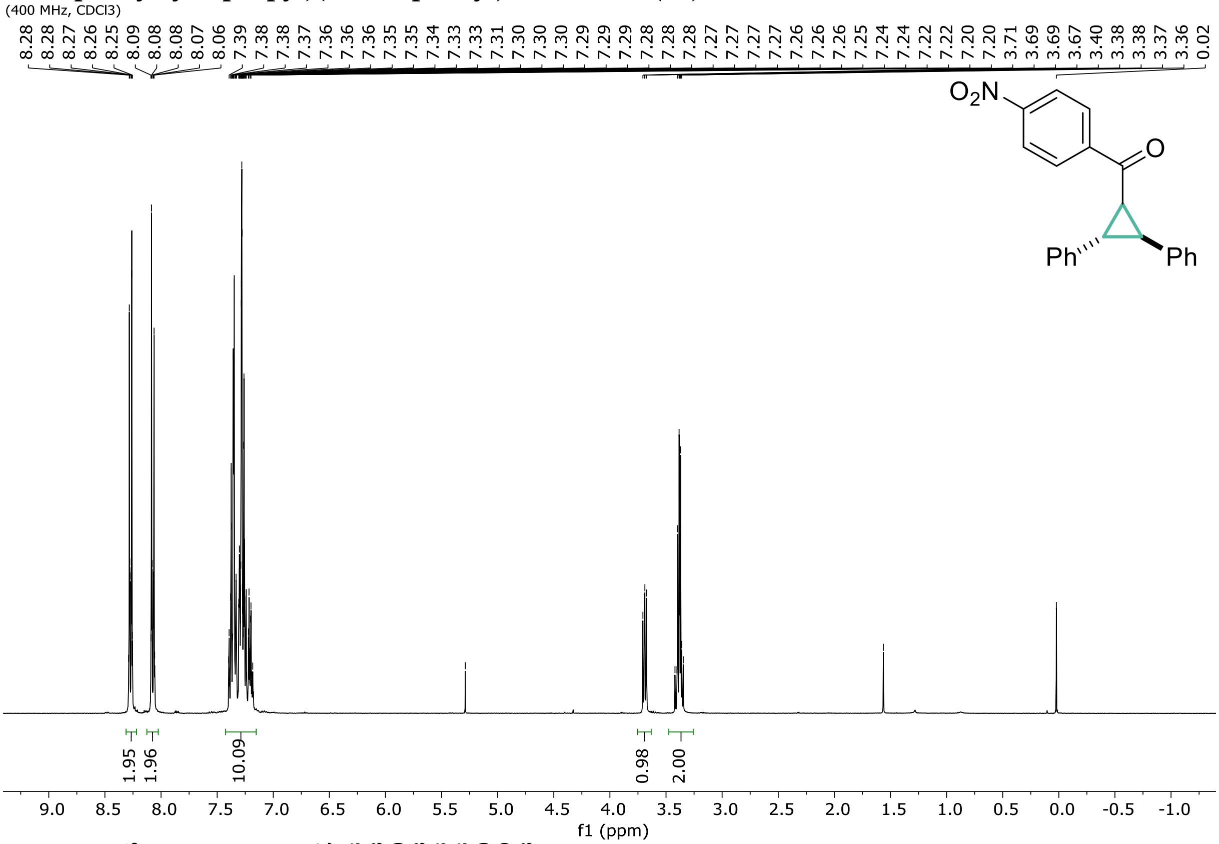

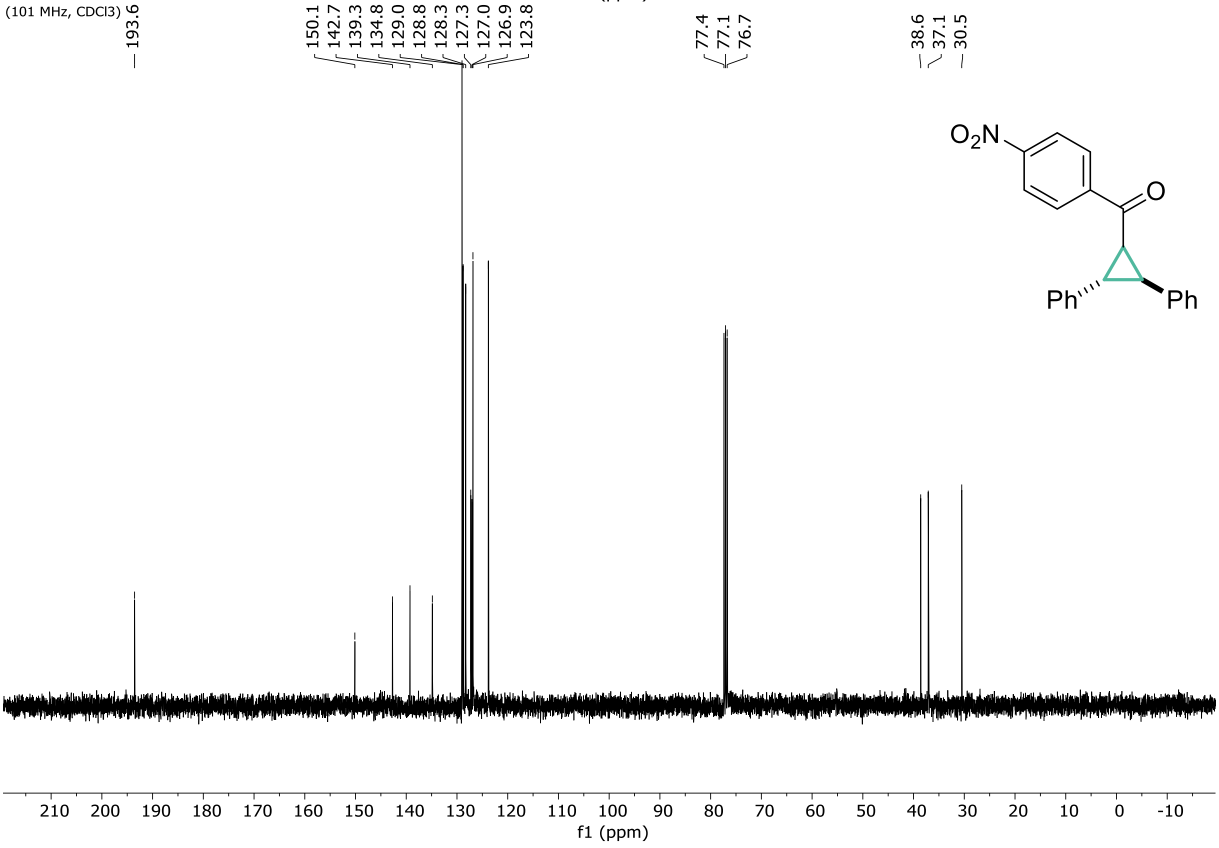

1-methoxy-4-(2-methyl-4-phenylcyclobutyl)benzene (26)

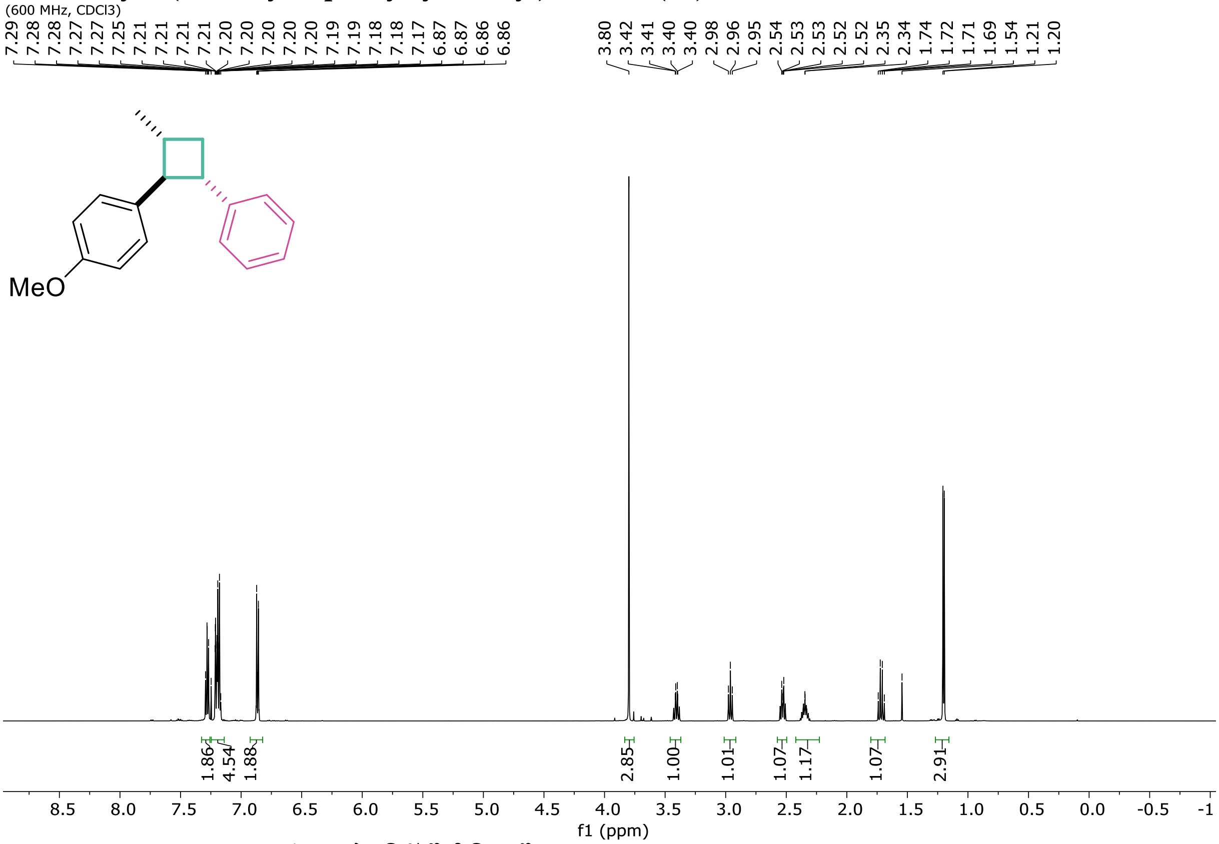

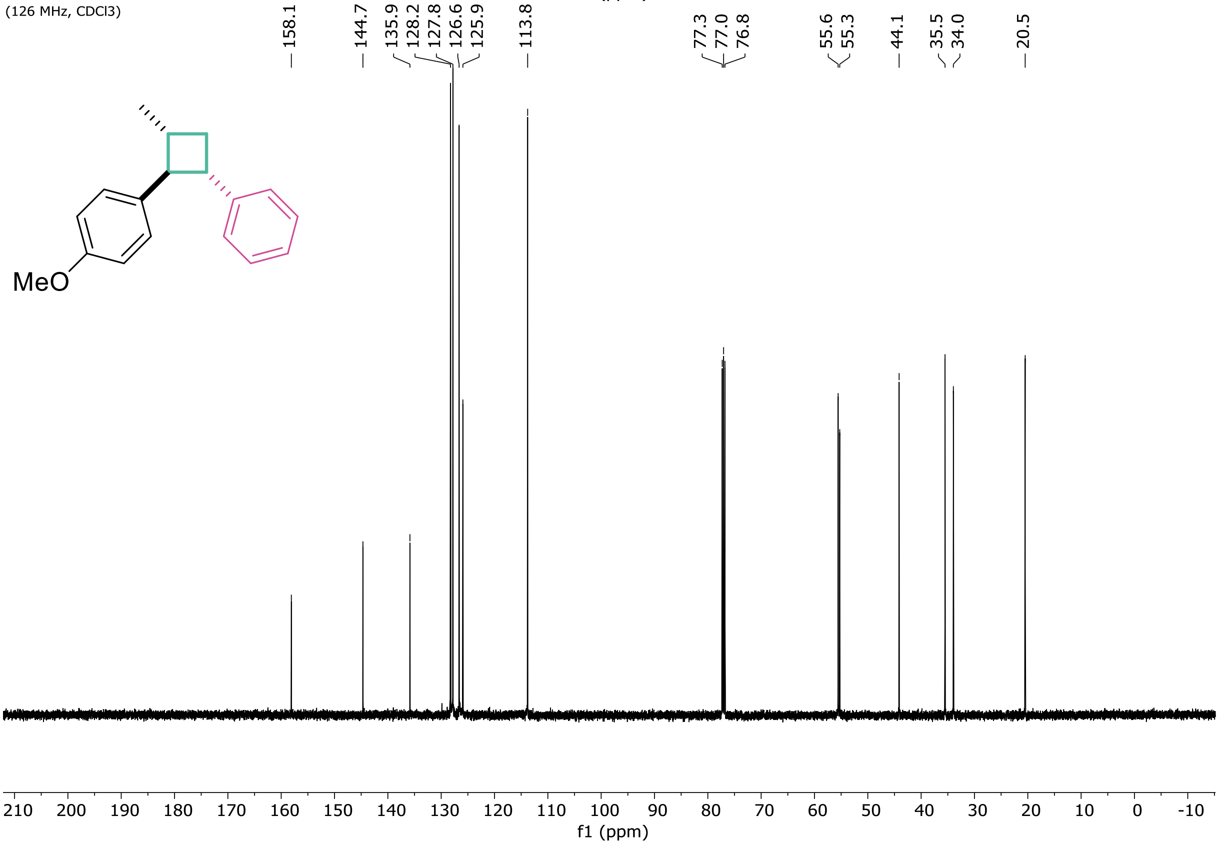

1-methoxy-4-(2-methyl-4-(p-tolyl)cyclobutyl)benzene (27)

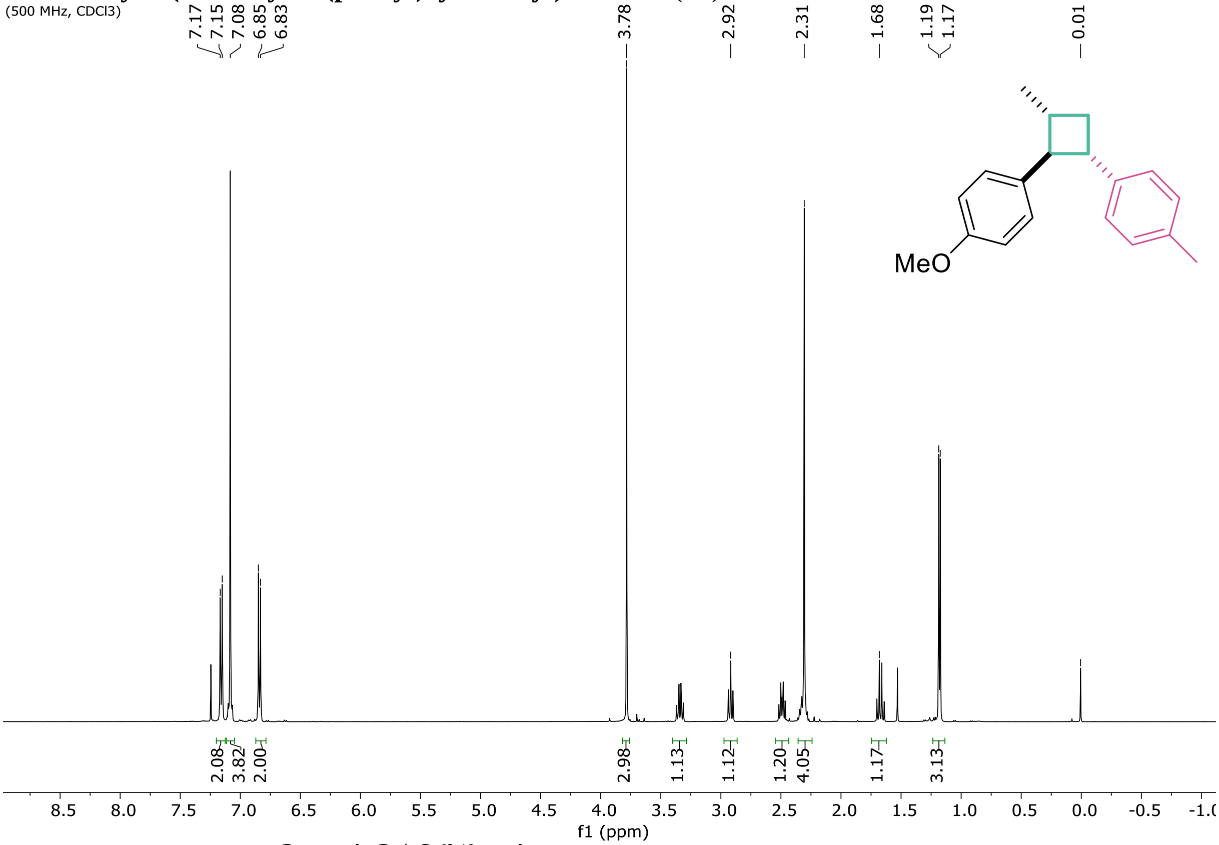

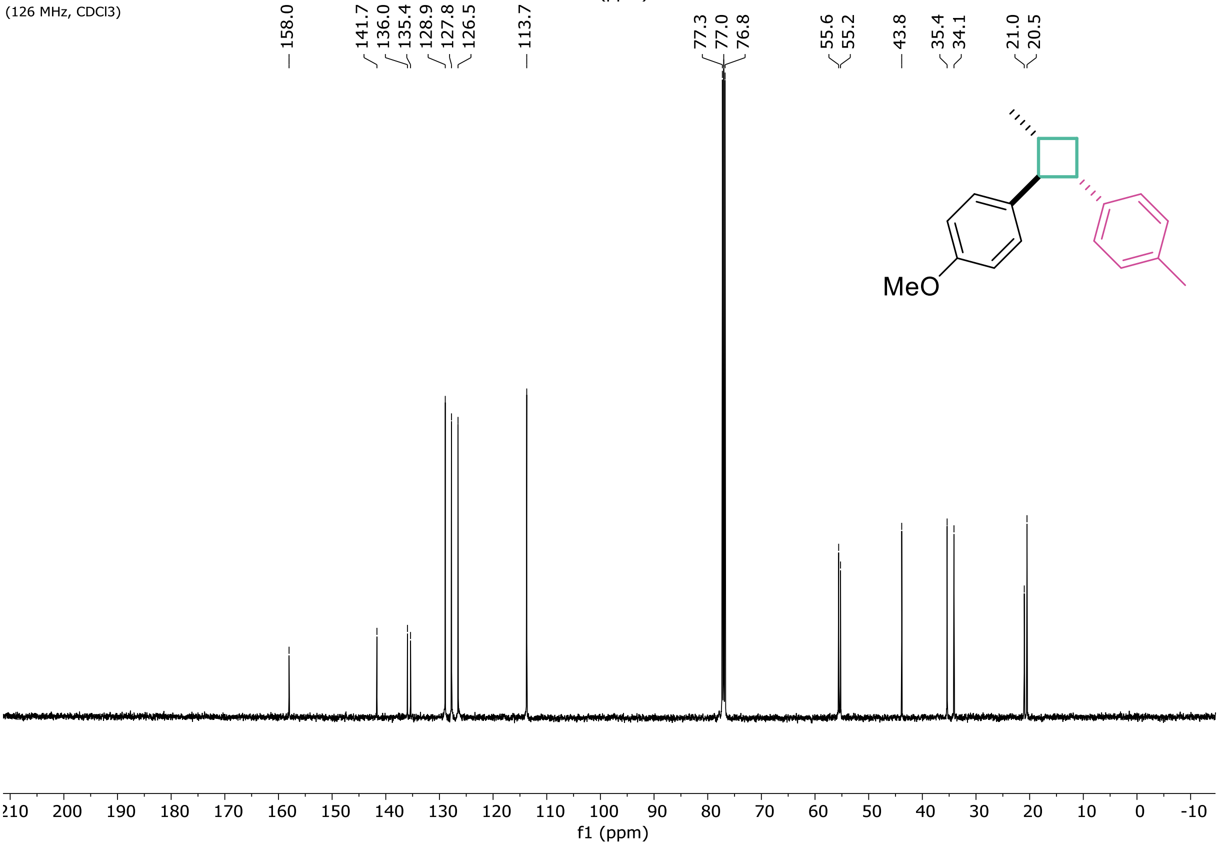

1-chloro-4-(2-(4-methoxyphenyl)-3-methylcyclobutyl)benzene (28)

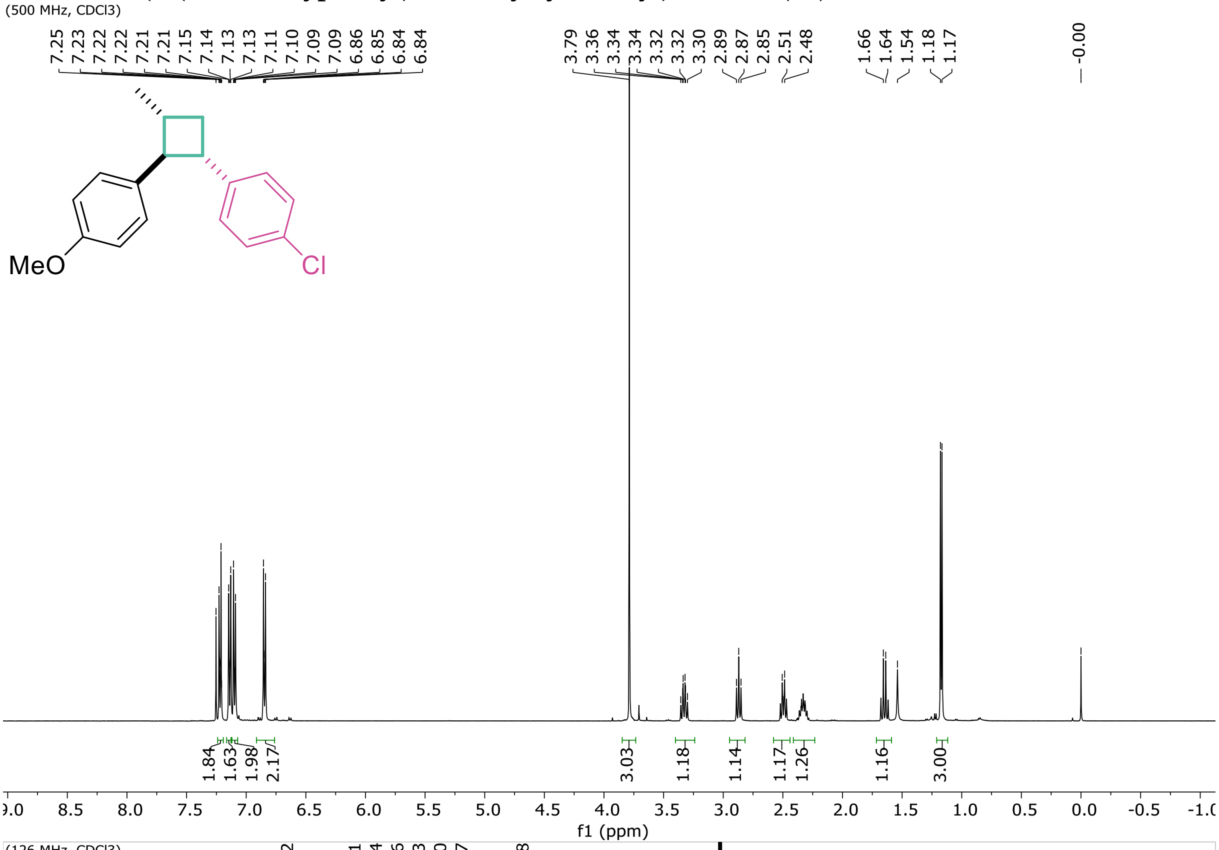

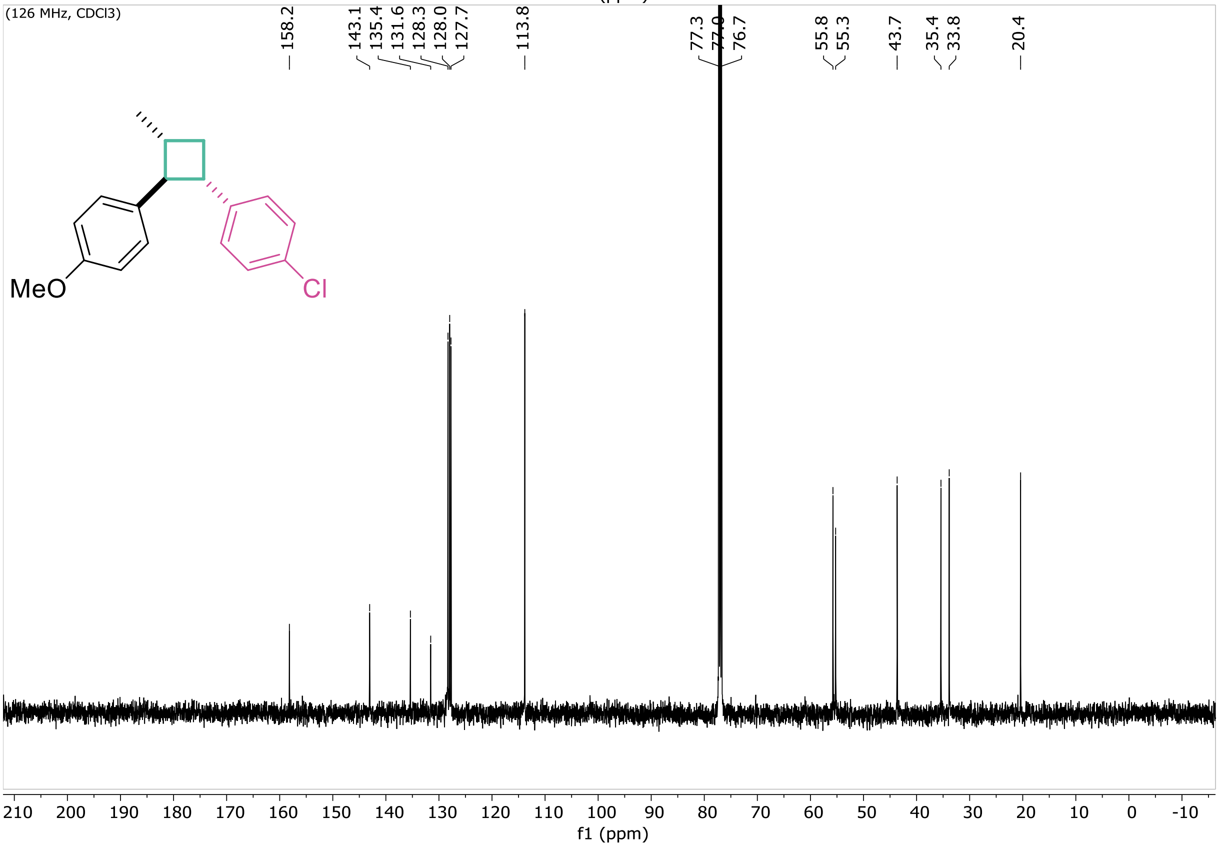

1-bromo-4-(2-(4-methoxyphenyl)-3-methylcyclobutyl)benzene (29)

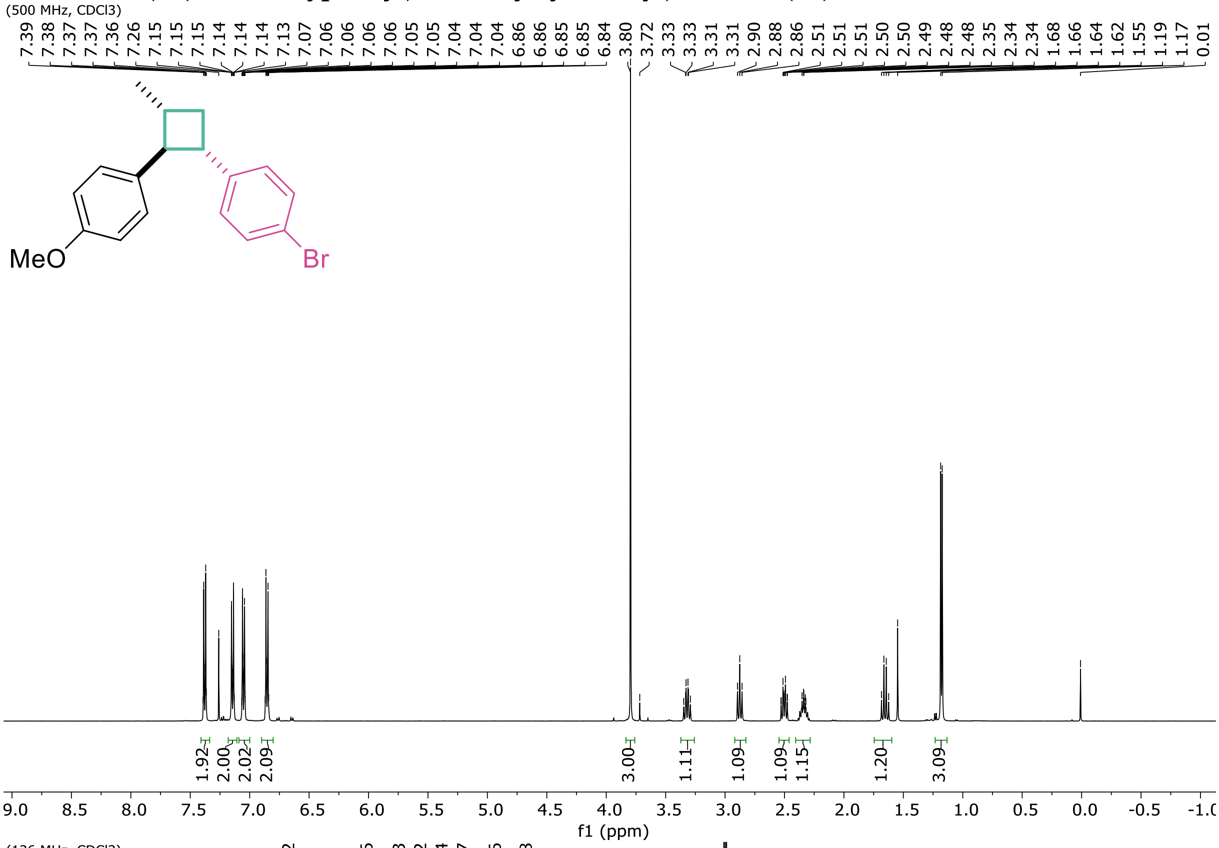

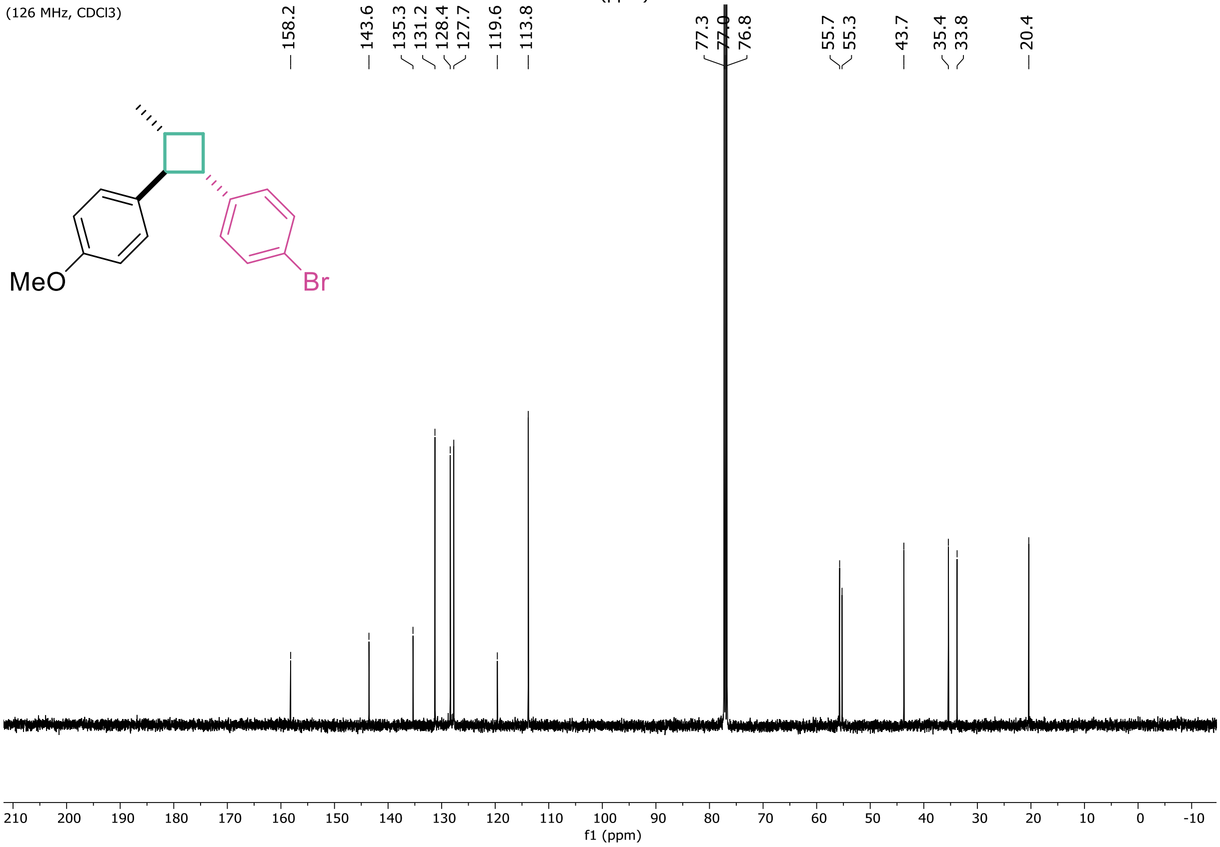

1-fluoro-4-(2-(4-methoxyphenyl)-3-methylcyclobutyl)benzene (30)

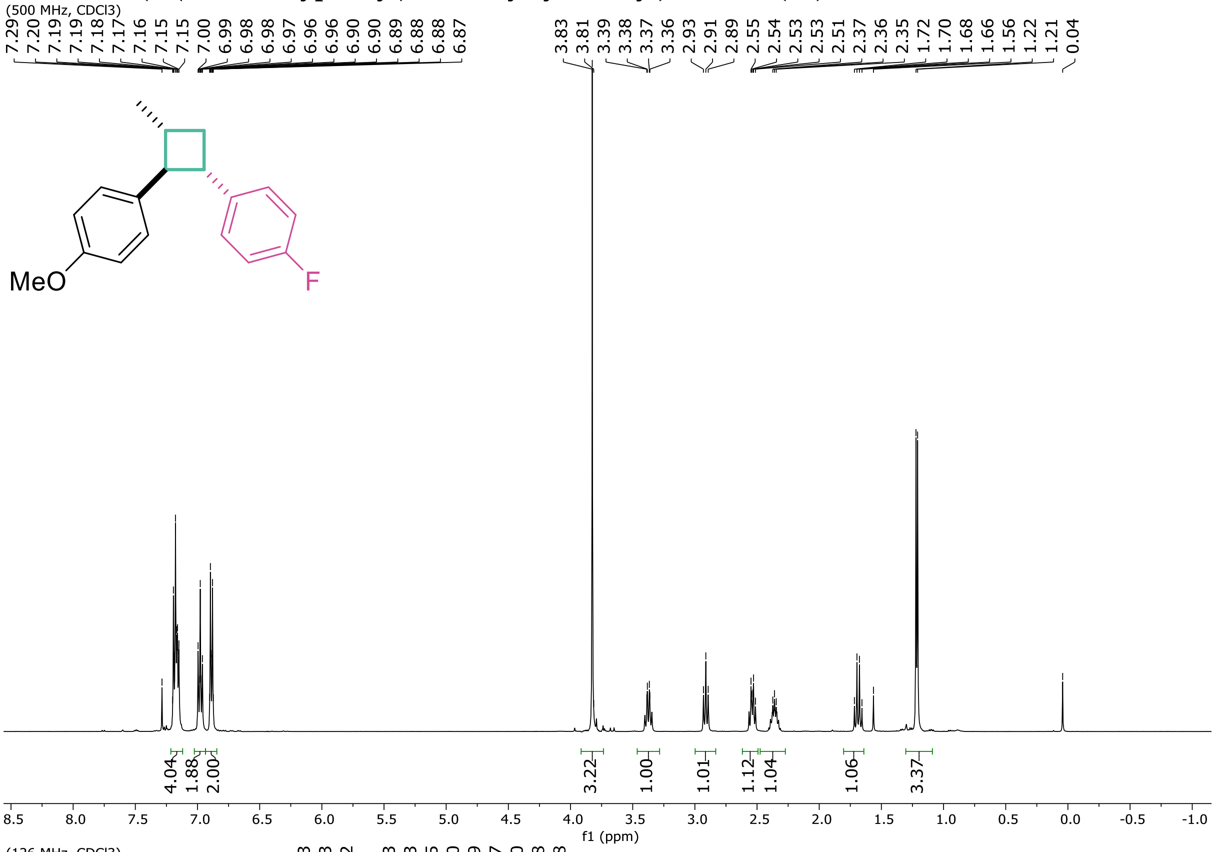

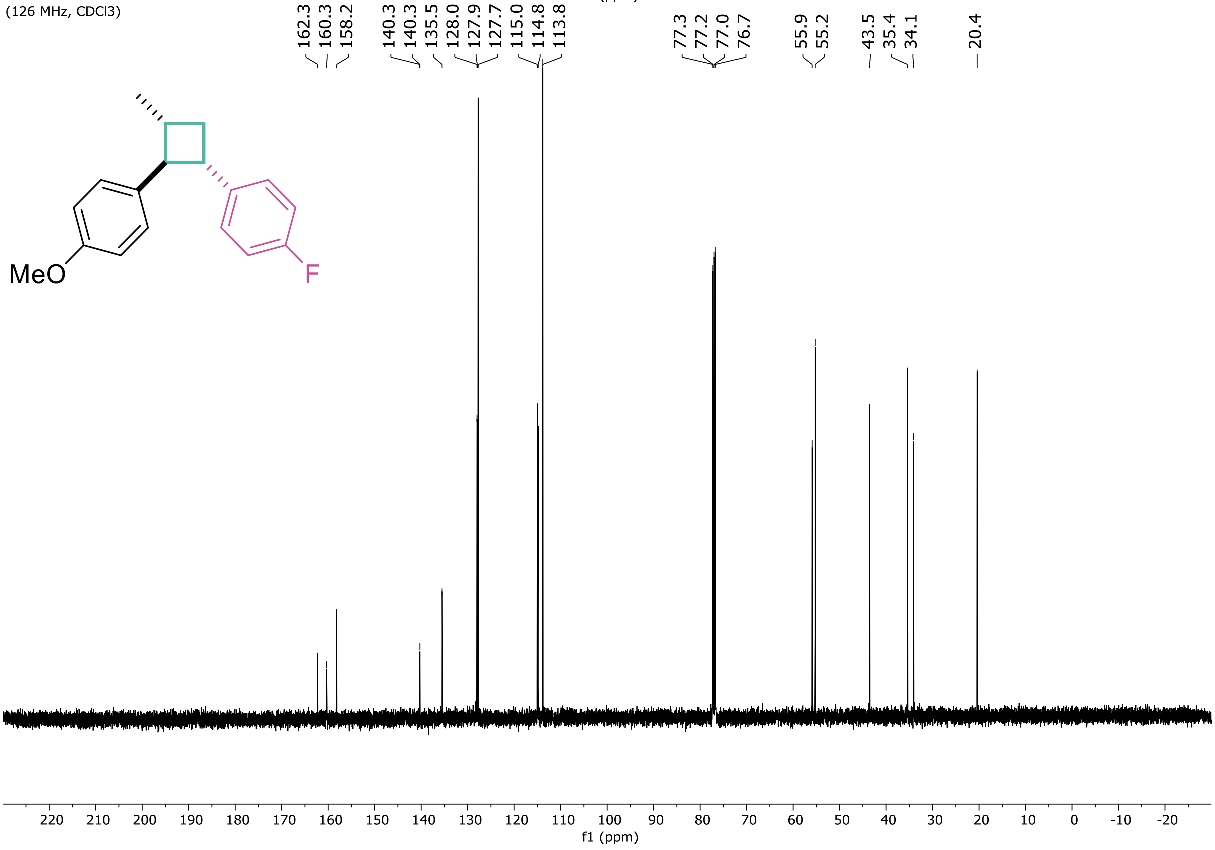

[1] K. Rybicka-Jasińska, Ł. W. Ciszewski, D. Gryko Photocatalytic Reaction of Diazo Compounds with Aldehydes. *Adv. Synth. Catal.* **2016**, *358*, 10, 1671-1678.

[2] A. C. de Mello, P.B. Momo, A. C. B. Burtoloso, G. W. Amarante Metal-Free Insertion Reactions of Diazo Carbonyls to Azlactones *J. Org. Chem.* **2018**, *83*, 18, 11399-11406.

[3] Y. H. Cho, J. H. Kim, H. An, K.-H. Ahn, E. J. Kang Cycloaddition Reactions of Alkene Radical Cations using Iron(III)-Phenanthroline Complex *Adv. Synth. Catal.* **2020**, *362*, 11, 2183-2188.

[4] Current Patent Assignee: PI INDUSTRIES LTD - WO2022/58878, 2022, A1

[5] Wang Yueh, Nathan L. Bauld Mechanistic Criteria for Cation Radical Reactions: Aminium Salt-Catalyzed Cyclopropanation *J. Am. Chem. Soc.* **1995**, *117*, 5671-5676.

[6] Johannes Schulz-Fincke at al, Structure-activity studies on N-Substituted tranylcypromine derivatives lead to selective inhibitors of lysine specific demethylase 1 (LSD1) and potent inducers of leukemic cell differentiation*European Journal of Medicinal Chemistry*, **2018**, *144*, 52-67.

[7] L.-W. Ye, X.-L. Sun, C.-Y. Li, Y. Tang Tetrahydrothiophene-Catalyzed Synthesis of Benzo[n.1.0] Bicycloalkanes *J. Org. Chem.* **2007**, *72*, 4, 1335-1340.

[8] Fransisco J. Sarabia, E. M. Ferreira Radical Cation Cyclopropanations via Chromium Photooxidative Catalysis *Org. Lett.* **2017**, *19*, 11, 2865-2868.

[9] Tanaka, K., Iwama, Y., Kishimoto, M., Ohtsuka, N., Hoshimo, Y., Honda, K. Redox Potential Controlled Selective Oxidation of Styrenes for Regio- and Stereoselective Crossed Intermolecular [2 + 2] Cycloaddition via Organophotoredox Catalysis. *Org. Lett.* **2020**, *22*, 13, 5207-5211.
